# Proteomic study of medicinal mushroom extracts reveals antitumor mechanisms in an advanced colon cancer animal model via ribosomal biogenesis, translation, and metabolic pathways

**DOI:** 10.3389/fphar.2024.1475102

**Published:** 2024-10-18

**Authors:** Boris Jakopovic, Anita Horvatić, Jurica Baranasic, Iris Car, Nada Oršolić, Ivan Jakopovich, Mirela Sedić, Sandra Kraljević Pavelić

**Affiliations:** ^1^ Dr Myko San – Health from Mushrooms Co., Zagreb, Croatia; ^2^ Faculty of Food Technology and Biotechnology, University of Zagreb, Zagreb, Croatia; ^3^ Laboratory for Cell Biology and Signalling, Division of Molecular Biology, Ruđer Bošković Institute, Zagreb, Croatia; ^4^ Centre for Applied Bioanthropology, Institute for Anthropological Research, Zagreb, Croatia; ^5^ Division of Animal Physiology, Faculty of Science, University of Zagreb, Zagreb, Croatia; ^6^ Faculty of Health Studies, University of Rijeka, Rijeka, Croatia

**Keywords:** colorectal cancer, medicinal mushrooms, proteomics, translation, cancer metabolism, unfolded protein response

## Abstract

**Introduction:**

Colorectal cancer ranks as the third most common cancer in both men and women, with approximately 35% of cases being stage IV metastatic at diagnosis. Even with treatment advancements, the survival rates for these patients remain suboptimal. There is a significant focus on developing multi-targeted therapies due to the common issue of drug resistance in standard and targeted cancer treatments. Medicinal mushrooms, both as single compounds and as complex extracts, have undergone extensive research. Numerous types of mushrooms have been shown to be safe, effective inhibitors of cancer pathways and strong enhancers of the immune system.

**Methods:**

In this study, we performed both qualitative and quantitative proteomic analyses using tandem mass tags (TMT) on CT26 wild type (CT26. WT) colon cancer tissues from Balb/c mice, which were treated with a special blend of medicinal mushroom extracts, either alone or in combination with the chemotherapy drug 5-fluorouracil.

**Results:**

The results showed a notable increase in survival rates and indicated that medicinal mushroom preparation Agarikon Plus, both alone and combined with 5-fluorouracil or another medicinal mushroom preparation Agarikon.1, impedes multiple key processes in colorectal cancer progression. The analysis of differentially expressed proteins in treated groups was done by use of bioinformatics tools and a decrease in ribosomal biogenesis (e.g., RPS3) and translation processes (e.g., RPL14) as well as an increase in unfolded protein response (e.g., DNAJC3), lipid metabolism (e.g., ACOT7), and the tricarboxylic acid cycle (e.g., FH) were observed.

**Conclusion:**

The treatment induced various alterations of known biomarkers and protein clusters critical to the progression and prognosis of colorectal cancer, laying a promising foundation for further translational research on this treatment modality.

## 1 Introduction

Historically, mushrooms have been an integral part of the diet and traditional healing practices for thousands of years across civilizations, prominently in East Asian countries like China, Japan, India, and Korea, serving as both a nutritional source and medicine (Badalyan and Rapior, 2021; Li et al., 2021). Among the known edible mushroom species, approximately 800 are recognized for their medicinal properties, often referred to as medicinal mushrooms (Wu et al., 2013). Scientific exploration into these fungi commenced around the 1960s in Japan and has since resulted in an extensive literature of over 50,000 publications.

Investigations have concentrated on the medicinal properties of mushroom extracts and their components, notably polysaccharides and polysaccharide-protein complexes. Certain extracts like lentinan from the Shiitake mushroom and polysaccharopeptide from the turkey tail mushroom (*Trametes versicolor*) have been used in clinical settings in East Asia since the 1970s and 1980s (Mizuno, 1999; Smith et al., 2002). Over 1,000 clinical studies have documented the various health benefits of mushroom preparations, revealing more than 130 therapeutic effects ranging from antioxidant and anti-inflammatory to antiviral and immunomodulatory activities (Poucheret et al., 2006; Ferreira et al., 2010; Zmitrovich et al., 2019).

These benefits are attributed to the mushrooms’ rich content of both high and low molecular weight compounds, including polysaccharides, proteins, lipids, and a diverse array of secondary metabolites like lectins, terpenoids, and alkaloids (Suay et al., 2000; Grienke et al., 2014). These secondary metabolites, of which abundance is often seen as a result of ecological adaptations, are derivatives of primary metabolite pathways and contribute to the fungi’s complex bioactivity. Notably, specific fungal metabolites, such as the antibiotics penicillin and cephalosporins, have become critical tools in modern medicine (Lewis, 2013).

Cancer designates several hundred diseases characterized by aberrant molecular pathways that regulate cell biology and coordination. These pathways, including crucial ones like nuclear factor-kappa B (NF-κB), mitogen-activated protein kinase (MAPK), protein kinase B (AKT), WNT, NOTCH, and tumor protein P53 (P53), are known to be influenced by bioactive compounds from medicinal mushrooms (Zaidman et al., 2005). Medicinal mushrooms target various cancer hallmarks; for instance, the *Trametes versicolor* extract significantly reduces proliferation in various breast cancer cells through B-cell lymphoma 2 (BCL-2) and P53 dependent apoptosis induction (Ho et al., 2005). Chronic inflammation, a known cancer risk factor, particularly in colorectal cancer, involves enzymes like cyclooxygenase-2 (COX-2), which is inhibited by constituents found in *Grifola frondosa* (Zhang et al., 2002).

Cancer’s ability to metastasize and form new blood vessels (angiogenesis) are key characteristics involving various molecules from both tumor cells and microenvironment. *Ganoderma lucidum* extracts have been shown to suppress these processes by modulating signaling pathways and downregulating molecules like transforming growth factor β1 (TGF-β1) and vascular endothelial growth factor (VEGF) (Stanley et al., 2005). These effects are attributed to its polysaccharides and triterpenoids, including a range of ganoderic and lucidenic acids (Weng and Yen, 2010). Additionally, *Ganoderma lucidum* impacts the WNT signaling pathway, crucial in colorectal cancer, by inhibiting axis inhibition protein 2 (AXIN2) expression and other downstream targets (Zhang, 2017).

Positive regulation of translation is another cancer characteristic which is crucial for cancer progression and metastasis, with pathways like mitogen-activated protein kinase (MAPK) and phosphoinositide 3-kinase/protein kinase B/mammalian target of rapamycin (PI3K/AKT/mTOR) converging on this process. Alterations in protein synthesis, particularly through the modulation of eukaryotic initiation factors (eIFs) and their regulators, are critical during cancer development (Silvera et al., 2010). *Ganoderma lucidum* has demonstrated the ability to reduce expression of mTOR and other related proteins important in protein synthesis, thus inhibiting tumor growth in experimental models (Suarez-Arroyo et al., 2013). Apoptosis induction is a primary target in cancer therapy, with substances like the D-fraction from *Grifola frondosa* showing promise by triggering cell death pathways in hepatocellular carcinoma cells (Zhao et al., 2017).

Colorectal cancer (CRC) is among the first three carcinomas by incidence and mortality in both genders (Siegel et al., 2023). In 2022, there were approximately 1.9 million new cases of colorectal cancer worldwide, and more than 930,000 deaths attributed to the disease. Colorectal cancer is the third most common cancer globally and the second leading cause of cancer-related deaths. By 2040, the burden is expected to rise significantly, with projections of over 3.2 million new cases and 1.6 million deaths annually (World Health Organization, 2023). A significant portion of these cases occur randomly, yet 3%–5% can be traced to genetic predispositions like Lynch syndrome, with less than 1% attributed to familial adenomatous polyposis (FAP) (Goudarzi et al., 2024). Approximately 20% of colorectal cancer (CRC) patients are diagnosed with stage IV metastatic disease at the time of their initial diagnosis. Moreover, a significant portion of patients who are diagnosed at stages II or III will eventually progress to stage IV metastatic disease, either during or after treatment. Even with improvements in treatment modalities, the five-year survival rate for metastatic CRC patients is a mere 15.1% (Feria and Times, 2023). This disease exemplifies the multi-stage progression of cancer, marked by a series of genetic and epigenetic alterations (Fearon and Vogelstein, 1990). Present-day management of CRC encompasses surgical intervention, radiation, chemotherapy, and precision medicine. Predominant chemotherapy agents include 5-fluorouracil (5-FU), leucovorin, irinotecan, oxaliplatin, and capecitabine, while common targeted treatments comprise monoclonal antibodies like bevacizumab and cetuximab, often administered alongside chemotherapy (Meyerhardt and Mayer, 2005; Douillard et al., 2013). 5-fluorouracil is a cytotoxic chemotherapeutic drug that belongs to the group of antimetabolites or pyrimidine base analogues. It is a thymidylate synthase (TS) inhibitor, which blocks synthesis of the pyrimidine thymidylate (dTMP), which is a nucleotide required for DNA replication. 5-FU is one of the most widely used cytostatics, and is used primarily in the treatment of breast cancer and gastrointestinal tumors. It is administered intravenously because its bioavailability is low after oral administration (de Oliveira et al., 2021). Fluorouracil (5-FU) is an essential component of systemic chemotherapy for colorectal cancer (CRC) in the palliative and adjuvant settings (Vodenkova et al., 2020). Adjuvant (post-operative) chemotherapy is recommended after curative tumor resection for all fit patients with stage III colon cancer, as well as for patients having stage II colon cancer with high-risk features (T4 tumor, tumor perforation, fewer than 12 removed lymph nodes (Stintzing, 2014). Although integrating targeted therapies with chemotherapy has shown efficacy in metastatic stages, several studies have not observed improved outcomes in adjuvant settings (Van Loon and Venook, 2011). The complexity of cancer biology often renders targeted treatments with limited overall benefits. Other challenges include resistance to therapies and toxicity issues, particularly with long-term chemotherapy. Various natural compounds and extracts, such as isoflavones, curcumin, EGCG, resveratrol, lycopene, and those derived from medicinal mushrooms, continue to be investigated as potential multi-targeted agents in cancer care (Chimento et al., 2023; Liu et al., 2024). Given the vast potential of bioactive compounds, and in the light of the fact that of about ∼3,000 druggable proteins only 5%–10% are currently targeted by current pharmaceuticals, the search for new therapeutic targets is of great importance (Oprea et al., 2018).

Numerous investigations have confirmed the anticancer potential of various medicinal mushrooms, attributing their effects to disruptions in tumor signaling and enhancement of the host’s antitumor immunity. Of recent, large scale proteomic studies have begun to emerge in the medicinal mushroom field, mostly on single mushroom species and their isolates (Jakopovic et al., 2023). However, large scale proteomic studies, as well as examining their impact on colorectal cancer (CRC) biomarkers which are commonly used in clinical settings are scarce, especially concerning blended multiple-species mushroom extracts which are theorized to have enhanced biological effects compared to singular extracts (Shamtsyan et al., 2004; Jakopovich, 2011). The scarcity extends to comprehensive data on the proteomic research of antitumor effects of standardized blended mushroom extracts, both individually and when used alongside conventional chemotherapy. Our previous work has outlined several anticancer properties of Agarikon.1 and Agarikon Plus, used alone or with 5-fluorouracil, demonstrating various immune responses including macrophage polarization, evidenced through nitric oxide and arginase measurements, and cytokine profiles, as well as its ability to inhibit angiogenesis, indicated by a significant reduction in vascular endothelial growth factor (VEGF) levels. Furthermore, Agarikon.1 has shown potent apoptosis induction in the SW620 metastatic human colorectal adenocarcinoma cell line (Jakopovic et al., 2020b). Further high-definition qualitative and quantitative proteomic research coupled with bioinformatic analysis on Agarikon.1 used alone or in combination with 5-fluorouracil identified significant processes, such as protein translation and glucose and lipid metabolism, important in colorectal cancer development and progression to be perturbed as a result of treatment (Jakopovic et al., 2020a). This follow-up research aims to reveal the antitumor mechanisms of blended medicinal mushroom preparation Agarikon Plus, alone and in combination with 5-fluorouracil, as well as in combination with Agarikon.1, on a proteomic level in this late-stage colorectal cancer model.

## 2 Materials and methods

### 2.1 Animals

The experiments were conducted on male Balb/c mice, approximately 2 months old, weighing 20–25 g from the Rudjer Boskovic Institute, Zagreb according to standard housing conditions. Experimental groups comprised 8–10 mice each. Animal studies were approved by the University’s of Zagreb, Department of Biology Ethics Committee (approval code: 251-58-10617-16-14) and performed in compliance with the guidelines in force in the Republic of Croatia (the Croatian Animal Welfare Law [NN, 135/2006 and 37/2013]) and according to the European Directive 2010/63/EU.

### 2.2 Tumor cells

Animals were inoculated with CT26. WT (ATCC^®^ CRL-2638™) which is a N-nitroso-N-methylurethane-(NNMU) induced murine colorectal cell line syngeneic with Balb/c mice. The cells were propagated and subcultured in accordance to the distributor’s protocol, i.e., they were grown in RPMI-1640 medium with 10% FBS, penicillin (100 U/mL) and streptomycin (100 μg/mL) and maintained at 37°C with 5% CO_2_ in a humidified atmosphere. After harvesting and preparation of cells, their total number and viability were determined by counting in a Neubauer chamber using Trypan Blue Dye, and was always found to be at least 95%. *Mycoplasma* contamination was assessed prior to the experimentation (MycoAlert™ PLUS *mycoplasma* detection kit, Lonza Walkersville, Walkersville, MD).

### 2.3 Tested substances

Medicinal mushroom extract mixtures Agarikon.1 (LOT:1100517) and Agarikon Plus (LOT: BXSM0901) were provided by Dr Myko San–Health from Mushrooms Co., Croatia. Agarikon.1 (AG) is registered by the Ministry of Health and Social Welfare of the Republic of Croatia as a dietary supplement (registration number MZ0813411210) (Jakopovich, 2011). It is produced from a hot water extract which is precipitated with ethanol and subsequently freeze-dried. This tablet preparation contains a mixture of *Lentinus edodes*, *Ganoderma lucidum*, *Agaricus brasiliensis* (=*blazei* ss. Heinem.), *Grifola frondosa*, *Pleurotus ostreatus*, and *Trametes versicolor* medicinal mushroom species, in equal amounts i.e. 125 mg each per tablet. One 1,000 mg tablet therefore contains 750 mg of mushroom polysaccharides per tablet, combined with excipients such as inulin, talc, magnesium stearate, and silica. Agarikon Plus (AP) (LOT: BXSM0901) is a proprietary liquid extract mixture of 10 mushroom species, including *Lentinus edodes*, *Ganoderma lucidum*, *Grifola frondosa*, *Trametes versicolor*, *Agaricus brasiliensis* (=*blazei* ss. Heinem.), *Meripilus giganteus*, *Pleurotus ostreatus*, *Fomes fomentarius*, *Phellinus linteus*, and *Tricholoma matsutake* in equal amounts. As described before (Durgo et al., 2013), 50 g of dried mushroom fruiting bodies are extracted in 1 L of boiling water for 24 h. Insoluble matter is removed by forcing the solution (in suspension form) through a filter press, and then concentrated 4-fold.

5-fluorouracil (Sandoz) was supplied at a concentration of 50 mg/mL in sterile aqueous solution, pH 8.6–9.0, and stored at 4°C in aluminum covered containers. Immediately prior to use, it was diluted in sterile distilled water.

### 2.4 Experimental design

Study groups were grown prior to treatment. The mice in the group study 1 (curative subsection) were injected subcutaneously in the right flank with 1 × 10^6^ viable CT26. WT cells in 100 μL of sterile phosphate-buffered saline (PBS) on day 0. Treatment of animals with tumors was started when the tumor was developed in 100% of the animals with a palpable solid tumor mass (≥700 mm^3^, 14 days post implantation), which is a highly advanced tumor stage. On day 14, mice were randomly divided into 5 groups (n = 8–10 mice/group) and treated with 10,400 mg/kg of Agarikon Plus (AP) diluted in PBS, 1,200 mg/kg of Agarikon.1 (AG) diluted in PBS by oral gavage for 14 days continuously, and with 5-fluorouracil (5-FU) intraperitoneally (30 mg/kg on days 1–4. and 15 mg/kg on 6, 8, 10 and 12 day of treatment). The treatment groups were treated with the following combinations of tested substances: AP, AP + 5-FU, AP + AG, and 5-FU. The control was given only saline by oral gavage. 5-FU was administered metronomically, and for both preparations the doses were calculated by interspecies allometric scaling (Nair and Jacob, 2016). The basis for the dose calculation of Agarikon.1 and Agarikon Plus was the recommended daily dose of these dietary supplements used by patients. The survival was monitored until day 55 after tumor inoculation, after which the remaining animals were euthanized. In order to analyze the inhibitory effects of tested substances on tumor growth, tumor length (L) and width (W) was measured, and tumor volume (mm^3^) was calculated as [V= (L × W^2^)/2]. Data was analyzed by Kruskal–Wallis ANOVA. Differences between the groups were assessed by multiple comparisons of mean ranks for all groups. Statistical analyses were done in STATISTICA 12 software (StatSoft, Tulsa, OK, United States) at significance *p* < 0.05.

In the second study, the same treatment was applied 14 days post tumor inoculation, i.e. 10,400 mg/kg of AP, 1,200 mg/kg of AG for the duration of 14 days, with or without 5-FU (30 mg/kg on days 1–4. and 15 mg/kg on 6, 8, 10 and 12 day of treatment), but the animals (n = 3 per group) were euthanized on the 28th day after tumor cell inoculation, after which the tumor tissues were collected and stored in liquid nitrogen immediately until proteomic analysis.

During euthanasia (study 1) or tumor collection (study 2), all animals were adequately anesthetized by intraperitoneal administration of a combination of Narketan^®^ Vetoquinol S.A., BP 189 Lure Cedex, France (active substance Ketamin) at a dose of 100 mg/kg and analgesic Xylapan^®^ (Vetoquinol Biowet, Gorzow, Poland) at a dose 5 mg/kg.

### 2.5 Survival analysis

Animal life span was evaluated by daily surveillance of spontaneous death or by selective euthanasia of animals showing signs of pain and suffering according to established criteria. Kaplan-Meier statistical analysis (log rank statistics) as well as overall survival were utilized to compare survival. Analyses were performed using MedCalc (Version 19.1.3) and the significance level was 5% (*p* < 0.05).

### 2.6 Preparation of tissue homogenates

Tumor tissue was mechanically grinded with liquid nitrogen and dissolved in 1 mL of a lysis buffer [7 M urea/2 M thiourea (Sigma-Aldrich, United States), 4% (w/v) CHAPS (Sigma-Aldrich, United States), 1% (w/v) dithiothreitol (DTT, United States) (Sigma-Aldrich), and 1 × protease inhibitor cocktail (Roche, Switzerland). The obtained lysate was sonicated (4 mm probe, power of 6 W; MicrosonTM, PGC Scientifics, United States), four times for 10 s. After sonication, the samples were incubated for 1 h at room temperature with gentle agitation (Eppendorf, Germany). Samples were centrifuged for 45 min at 14,000 rpm and 4°C (Eppendorf, Germany) and the supernatant was collected for further analyses. Protein concentrations were determined using the Qubit™ fluorometric (Invitrogen, United States) quantitation platform.

### 2.7 Preparation of samples and mass spectrometry analysis

Internal standard was prepared by mixing equal amounts of each sample used in this study. From each sample, an amount of 35 µg total proteins was acetone precipitated overnight. The pellets were collected by centrifugation (8,000 × g, 4°C), dissolved in 100 μL 0.1 M triethyl ammonium bicarbonate (TEAB, Thermo Scientific, Rockford, United States), and subjected to reduction and alkylation (200 mM DTT and 375 mM IAA, respectively, reagents obtained from Sigma Aldrich, St. Louis, MO, United States). Protein digestion was performed using trypsin (Promega, Madison, United States; trypsin-to-protein ratio 1:35) at 37°C overnight. Tryptic peptides were dried, dissolved in 100 µL of 0.1 M TEAB and labelled using 10-plex Tandem Mass Tag reagents (Thermo Scientific, Rockford, United States) according to manufacturer instructions. In short, Tandem Mass Tags (TMT) label reagents were equilibrated at room temperature, resuspended in anhydrous acetonitrile LC-MS grade (Thermo Scientific, Rockford, United States) and added to each sample. Labelling was performed for 1 h at room temperature and then quenched by adding 5% hydroxylamine (Thermo Scientific, Rockford, United States) for 15 min. Internal standard was labelled with TMT 126. Samples were then combined at equal amounts (each TMT experiment containing randomized TMT-labelled samples and internal standard) and 5 μg of each mixed sample set was vacuum dried and stored at −80°C for LC-MS/MS analysis. High resolution LC-MS/MS analysis of TMT-labelled peptides was carried out using an Ultimate 3,000 RSLCnano system (Dionex, Germering, Germany) coupled to a Q Exactive Plus mass spectrometer (Thermo Fisher Scientific, Bremen, Germany) as described elsewhere (Horvatić et al., 2019).

### 2.8 Protein identification and quantification

For peptide identification and relative quantification, the SEQUEST algorithm implemented into Proteome Discoverer (version 2.3, Thermo Fisher Scientific) was used. Database search against *Mus musculus* FASTA files downloaded from NCBI database (11/4/2019, 185,475 entries) was performed according to the following parameters: two trypsin missed cleavage sites, precursor and fragment mass tolerances of 10 ppm and 0.02 Da, respectively; carbamidomethyl (C) fixed peptide modification, oxidation (M), and TMT sixplex (K, peptide N-terminus) dynamic modifications. The false discovery rate (FDR) for peptide identification was calculated using the Percolator algorithm within the Proteome Discoverer workflow and was set at 1% FDR. Only proteins with at least two unique peptides and 5% FDR were reported as identified. Protein quantification was accomplished by correlating the relative intensities of reporter ions extracted from tandem mass spectra to that of the peptides selected for MS/MS fragmentation. The internal standard was used to compare relative quantification results for each protein between the experiments. The mass spectrometry proteomics data have been deposited to the ProteomeXchange Consortium via the PRIDE partner repository with the dataset identifier PXD049261.

### 2.9 Western blot analysis

For Western blot analysis, a total of 50 µg proteins was resolved on 12% SDS polyacrylamide gels and transferred onto PVDF membranes (Bio-Rad, Hercules, CA, United States). Membranes were blocked in 5% bovine serum albumin (PAN-Biotech, Aidenbach, Germany) or 5% non-fat milk (Bio-Rad, United States) prepared in TBST buffer, and probed with primary antibodies against APOA2 (Cat# STJ118035, St. John’s Laboratory, London, United Kingdom), DNAJC-3, FH, RPS3 (ab154714 DNAJC-3, ab233394 FH, ab181992 RPS3, Abcam, Cambridge, United Kingdom) and ACOT7 (Cat# ABIN7111284 Antibodies-online, Aachen, Germany) overnight at 4°C. All antibodies were diluted at 1:1,000. The next day, membranes were washed with TBST buffer and probed with secondary antibody goat anti-mouse or goat anti-rabbit (Cat# 7074 for goat anti-rabbit secondary antibody, Cat# 7076S for goat anti-mouse secondary antibody dilution 1:2,000, Cell Signaling Technologies, Danvers, MA, United States) for 1 h at room temperature. Afterwards, protein bands were visualized using Amersham™ ECL™ Prime Western blotting Detection Reagent (Cytiva, Little Chalfont, United Kingdom) and Amersham Imager 600 (GE Healthcare, Chicago, IL, United States). Relative protein expression was analyzed by Quantity One 1-D Analysis Software version 4.6.6 (Bio-Rad, United States). Briefly, prior to antibody incubation, membranes were stained with Coomassie and visualized using Amersham Imager 600. Obtained images were densitometrically analyzed to obtain the total protein load per lane for each sample. Stained membranes were then thoroughly washed with TBST to remove Coomassie stain followed by blocking and probing with antibodies as described above. Densitometry data of protein bands was normalized against total protein load per lane. All experiments were performed in two biological replicates run in technical duplicates. Statistical analysis was performed by ANOVA (*p* < 0.05).

### 2.10 Statistical analysis

Protein abundances data was normalized by use of the internal standard. After exclusion of outliers using Dixon test and proteins with less than two unique peptides, Kruskal–Wallis test was performed to test the difference in protein abundance between the groups. For proteins that were significantly different, Conover *post hoc* test was performed for pairwise multiple comparisons. In all cases, values of *p* < 0.05 were considered significant. Fold changes (FC) have been calculated as follows: FC = log2 [mean (Treatment)/mean (Control)]. Statistics were performed using Rstudio (v3.2.2). Differences in relative expression status of proteins obtained by Western blot analysis were analyzed by ANOVA in Microsoft Excel. Statistical significance was set at *p* < 0.05.

### 2.11 Bioinformatic analysis

Proteins GI accession numbers were converted into official gene symbol by DAVID conversion tool (https://david.ncifcrf.gov/conversion.jsp). If any protein GI accession number was not identified, it was run through SmartBLAST to identify highly similar proteins (https://blast.ncbi.nlm.nih.gov/smartblast/). In order to assign biological functions, identified proteins were further subjected to UniProt (http://uniprot.org/) and PANTHER (http://pantherdb.org) database searches. Enrichment analysis of protein-protein interaction networks was performed using STRING database v12.0 (https://stringdb.org/), with the selection of appropriate organism and default settings with the exception of no more than 5 interactors to show in 1st shell and the minimum required interaction score set to high (0.700). Pathway analyses for up- or down-accumulated clusters of proteins were performed for each treatment group separately using REACTOME software (http://www.reactome.org/) and STRING database. REACTOME performs an enrichment test to determine whether any REACTOME pathways are enriched in the submitted data. A binomial test was used to calculate the probability. The *p*-values are corrected for multiple testing (Benjamini-Hochberg procedure) that arises from evaluating the submitted list of identifiers against every pathway. The pathway with the corrected *p*-value less than 0.05 was considered to be significantly enriched.

## 3 Results

### 3.1 Survival and tumor volume analysis

**FIGURE 1:**
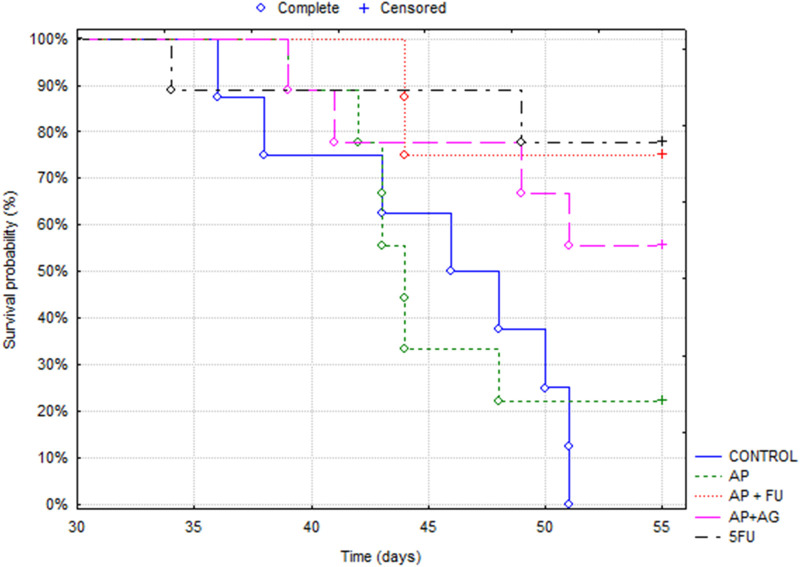
Kaplan-Meier survival curves of mice bearing CT26 WT tumors in different treatment groups.

**FIGURE 2:**
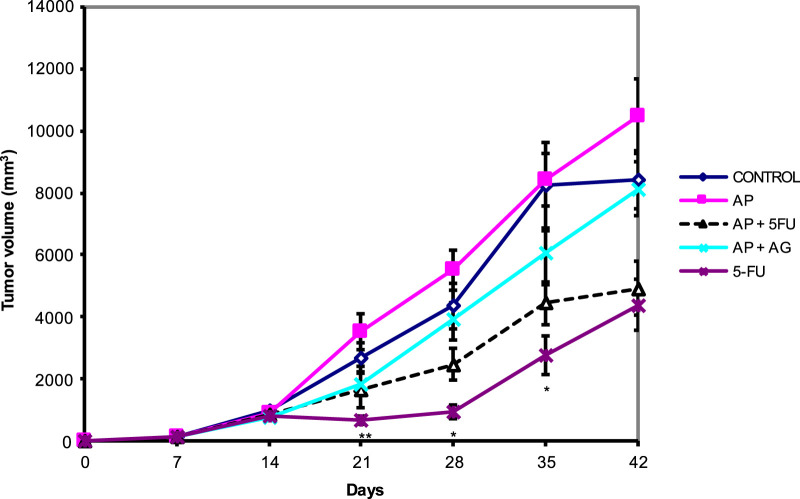
Effect of Agarikon Plus (AP), Agarikon.1 (AG), 5-fluorouracil (5-FU), and their combination on tumor volume. Treatment started 14 days after s.c. inoculation of CT26 WT (1 × 10^6^/mouse) cells with 1,200 mg/kg of Agarikon 1 by oral gavage for 14 days continuously, or with 5-FU intraperitoneally at dose of 30 mg/kg on days 1–4. and 15 mg/kg on 6, 8, 10, and 12 day of treatment. Volumes were determined once weekly for 6 weeks. *p <; 0.05; **p < 0.01; ***p < 0.001, versus control group. Bars show means ± SEM.

Mice bearing CT26 wild type (CT26. WT) tumors administered with Agarikon.1 (AG), Agarikon Plus (AP) or 5-fluorouracil for 2 weeks after tumor inoculation showed a significant increase in life span in most curative groups (Figure 1; Table 1). In most of the treated groups, a statistically significant overall survival (OS) was noted (*p* < 0.05; log-rank test), with the best effect observed in group treated with Agarikon Plus (AP) and 5-fluorouracil (5-FU) (OS = 87.5%), followed by the group treated with 5-FU (OS = 77.7%) and the group treated with a mixture of Agarikon.1 and Agarikon Plus (OS = 55.5%), compared to the control group, where there were no more surviving animals 55 days post tumor inoculation. The weakest effect on survival was recorded in the group treated with AP alone (OS = 22.2%). During the monitoring of tumor volume, smaller tumor volume was noted in most treated groups in comparison with control, with the exception of group treated with Agarikon Plus. In addition to that group, the groups treated with Agarikon Plus and 5-fluorouracil had the smallest increase in tumor mass. Statistical significances are shown in Figure 2. The first two measurements were taken during the treatment. It was noted that in the groups where 5-fluorouracil was used, there was an acceleration of the growth of the tumor mass after the end of treatment. Accordingly, 5-fluorouracil has the strongest cytotoxic effect on tumor cells, the effect of which weakens after the end of the application, which is evident from the subsequent acceleration of the growth kinetics of the tumor volume. In the groups treated with a mixture of Agarikon.1 and Agarikon Plus extracts only, the tumor volume is smaller compared to the control until the last measurement, and there is no acceleration of tumor growth after the end of the therapy.

**TABLE 1:** Survival analysis of Balb/c mice bearing CT26.WT after treatment.

Experimental group analysis	Overall survival rates	Kaplan-Meier	
Curative subsection	% Of survivors on day 55. after tumor inoculation	*p*	*χ* ^2^
Control	0	NA	NA
AP	22.2	0.7773	0.08001
AP + 5-FU	87.5	0.0008	11.2060
AP + AG	55.5	0.0224	5.2170
5-FU	77.7	0.0028	8.9481

AP, Agarikon Plus; AG, Agarikon.1; 5-FU, 5-fluorouracil.

### 3.2 Proteomics

After exclusion of isomers and unnamed protein products, tandem mass tag proteomic analysis of tumor tissue revealed a total of 98 up- or downregulated proteins between the treatment groups and control mice, out of which 55 were up- and 43 were downregulated (Tables 2–5). These proteins have various molecular and biological functions, determined by the Uniprot database search, as well as by use of PANTHER GO analysis (Supplementary Figure S1). In the AP group (Table 2; Supplementary Figure S1A, B), differential proteins in abundance (upregulated) are classified into five categories according to molecular function: ATP-dependent activity, binding, catalytic activity, molecular function regulatory activity, and transporter activity. These are involved in various biological processes, such as biological regulation, cellular process, homeostatic process, localization, metabolic process (i.e., carboxylesterase 1C), multicellular organismal process, pigmentation, response to stimulus. Downregulated proteins in this group are involved in seven molecular functions: ATP-dependent activity, binding, catalytic activity, molecular transducer activity, structural molecule activity, translation regulator activity, transporter activity and are implicated in the following biological processes: biological process involved in interspecies interaction between organisms, biological regulation, cellular process, developmental process, immune system process, localization, metabolic process (i.e., ATP synthase-coupling factor 6, mitochondrial), multicellular organismal process, reproduction, reproductive process, and response to stimulus. Upregulated proteins in the group treated with both AP and 5-FU are classified into seven categories according to molecular function (ATP-dependent activity, binding, catalytic activity, cytoskeleton motor activity, molecular adaptor activity, structural molecule activity and transcription regulator activity) and eight according to biological process [biological regulation, cellular process such as protein folding (DnaJ homolog subfamily C member 3), detoxification, developmental process, homeostatic process, metabolic process (fumarate hydratase, mitochondrial), multicellular organismal process and response to stimulus]. Downregulated proteins in the same group are assigned eight molecular functions (ATP-dependent activity, binding, catalytic activity, cytoskeletal motor activity, molecular function regulatory activity, molecular transducer activity, structural molecule activity and transporter activity) and thirteen biological processes [biological regulation, cellular process (large ribosomal subunit protein L3), developmental process, growth, homeostatic process, immune system process (annexin A1), localization, locomotion, metabolic process, multicellular organismal process, reproduction, reproductive process and response to stimulus] (Table 3; Supplementary Figure S1C, D). Upregulated proteins in the group treated with both AG.1 and AP (Table 4; Supplementary Figure S1E, F) are classified into nine categories with regards to molecular function (ATP-dependent activity, antioxidant activity, binding, catalytic activity, molecular function regulator activity, molecular transducer activity, structural molecule activity, transcription regulator activity) and are implicated in the following biological processes: biological process involved in interspecies interaction between organisms, biological regulation, cellular process (i.e., nucleophosmin), developmental process, homeostatic process, immune system process (i.e., complement C3), localization, locomotion, metabolic process (i.e., DNA replication licensing factor MCM6), multicellular organismal process, pigmentation, and response to stimulus. Downregulated proteins of the same group possess following molecular functions (ATP-dependent activity, binding, catalytic activity, cytoskeletal motor activity, molecular function regulator activity, molecular transducer activity, structural molecule activity and transporter activity) and are involved in following biological processes [biological regulation, cellular process (i.e., cytochrome c oxidase subunit 4 isoform 1, mitochondrial), developmental process, growth, homeostatic process, immune system process (i.e., annexin A1), localization, locomotion, metabolic process, multicellular organismal process, reproduction, reproductive process and response to stimulus]. Finally, molecular processes affected by the upregulated proteins in the 5-FU group (Table 5; Supplementary Figure S1G, H) include binding, catalytic activity, molecular function regulator activity, structural molecule activity, and transcription regulator activity, and are involved in the following biological processes: biological regulation, cellular process, detoxification, developmental process, homeostatic process, localization, metabolic process (i.e. 60S ribosomal protein L29, carboxylesterase 1C) and response to stimulus. Downregulated proteins in the same group possess the following molecular functions (binding, catalytic activity, molecular transducer activity) and are involved in the following biological processes [biological process involved in interspecies interaction between organisms, biological regulation, cellular process, developmental process, homeostatic process, immune system process, metabolic process (i.e., protein S100-A9), multicellular organismal process and response to stimulus].

**TABLE 2:** List of differentially regulated proteins in AP treated group vs*.* control group. Positive fold-change value denotes up-accumulation and negative fold change value denotes down-accumulation in comparison with control.

Gene symbol	Protein name	*p*-value (FDR)	Fold change (log2 vs*.* control)	Biological process
Apoa2	Apolipoprotein A-II	0.00415844	1.43	Lipid transport and metabolism, retinoid transport, chylomicron remodeling
Acot7	Cytosolic acyl coenzyme A thioester hydrolase	0.028980935	1.18	Fatty acid metabolism, lipid metabolism
Ces1c	Carboxylesterase 1C	0.023659294	1.07	Lipid catabolism, response to metal ions
Rab11b	Ras-related protein Rab-11B	0.035000507	1.03	Intracellular transport of proteins, organization of vesicles
Rab11a	Ras-related protein Rab-11A	0.028005962	0.98	Cell cycle, protein transport
Dnajc3	DnaJ homolog subfamily C member 3	0.013974559	0.81	Reaction to stress, regulation of translation, unfolded protein stress
Fh1	Fumarate hydratase, mitochondrial	0.034636547	0.77	DNA damage, DNA repair, tricarboxylic acid cycle
Ighm	Immunoglobulin heavy constant mu	0.034548178	0.53	Immune response
Mcm6	DNA replication licensing factor MCM6	0.0491043	0.41	Cell cycle, DNA replication
P4hb	Protein disulfide-isomerase	0.041677687	0.25	Protein folding, ER stress response
Hspa5	Endoplasmic reticulum chaperone BiP	0.044074358	0.18	Unfolded protein response (UPR)
Rplp2	Large ribosomal subunit protein P2	0.042595638	0.14	Ribosome biogenesis, translation
Gm6525	Predicted pseudogene 6525	0.007133671	−0.30	Ribosome biogenesis, translation
Atp5j	ATP synthase-coupling factor 6, mitochondrial	0.008585462	−0.32	Ion transport, ATP metabolism
Rps3	Small ribosomal subunit protein S3	0.005865099	−0.47	Regulation of transcription and translation, cell cycle
Hnrnpk	Heterogeneous nuclear ribonucleoprotein K	0.010241351	−0.57	mRNA processing, mRNA splicing, transcription, regulation of transcription
Hmgb1	High mobility group protein B1	0.004082575	−0.72	Innate and acquired immunity, autophagy, chemotaxis, DNA damage, recombination and repair, immunity, inflammatory response
Anxa7	Annexin A7	0.027257018	−0.84	Cell signaling, cell proliferation, integrin binding, ECM interactions
Hnrnpd	Heterogeneous nuclear ribonucleoprotein D0	0.004487521	−1.07	Biological rhythms, regulation of transcription
Rpl36a	Ribosomal protein L36A	0.012994618	−1.08	Translation, ribosome biogenesis
Cdc42	Cell division control protein 42 homolog	0.032738061	−1.11	Actin cytoskeleton organization, intercellular adhesion, regulation of cell growth and division, DNA replication, angiogenesis
Ddx3y	ATP-dependent RNA helicase DDX3Y	0.026644555	−1.22	Initiation of translation
Syncrip	Heterogeneous nuclear ribonucleoprotein Q	0.021065728	−1.76	RNA processing, translation

**TABLE 3:** List of differentially regulated proteins in AP and 5-FU treated group vs. control group. Positive fold-change value denotes up-accumulation and negative fold change value denotes down-accumulation in comparison with control.

Gene symbol	Protein name	*p-*value (FDR)	Fold change (log2 vs*.* control)	Biological process
Apoa2	Apolipoprotein A-II	0.00415844	1.60	Lipid transport and metabolism, retinoid transport, chylomicron remodeling
Dnajc3	DnaJ homolog subfamily C member 3	0.013974559	1.41	Reaction to stress, regulation of translation, unfolded protein stress
Myh1	Myosin-1	0.018684407	1.29	Muscle contraction
Myl1	Myosin light chain 1/3, skeletal muscle isoform	0.039277054	1.06	Muscle contraction
Eno3	Beta-enolase	0.027647663	0.99	Glycolytic process, drug response, hypoxia tolerance
Tpm2	Tropomyosin beta chain	0.035409508	0.96	Muscle contraction, organization of actin filaments
Ces1c	Carboxylesterase 1C	0.023659294	0.89	Lipid catabolism, response to metal ions
Etfb	Electron transfer flavoprotein subunit beta	0.045959108	0.87	Beta-oxidation of fatty acids, electronic transport chain, ECM organization
Mt1	Metallothionein-1	0.012014847	0.87	Cellular homeostasis of metal ions, signal transduction mediated by nitric oxide
Ttn	Titin	0.040679239	0.70	Muscle contraction, organization of actin filaments
Acot7	Cytosolic acyl coenzyme A thioester hydrolase	0.023926388	0.59	Fatty acid metabolism, lipid metabolism
Fh1	Fumarate hydratase, mitochondrial	0.034636547	0.58	DNA damage, DNA repair, tricarboxylic acid cycle
Hpx	Hemopexin	0.042065903	0.44	Iron homeostasis, immune response
Gm6525	Predicted pseudogene 6525	0.007133671	−0.26	Ribosome biogenesis, translation
Rps18-ps3	Ribosomal protein S18, pseudogene 3	0.004987548	−0.31	Translation
Tagln2	Transgelin-2	0.020642272	−0.34	ECM organization, muscle contraction
Pdia3	Protein disulfide-isomerase A3	0.037303139	−0.36	Cellular redox homeostasis, immune response, protein folding
Cap1	Adenylyl cyclase-associated protein 1	0.021324412	−0.43	Polymerization or depolymerization of actin, amoeboid cell migration, organization of the actin cytoskeleton
Myh9	Myosin-9	0.027072282	−0.44	Organization of the actin cytoskeleton
Hspa9	Stress-70 protein, mitochondrial	0.032419724	−0.45	Chaperone cofactor-dependent protein folding, unfolded protein cellular response, negative regulation of cell death
Hmgb2	High mobility group protein B2	0.002879702	−0.46	Chemotaxis, DNA recombination, immunity, inflammatory response, innate immunity, regulation of transcription
Anxa1	Annexin A1	0.005184115	−0.47	Immune response, inflammation
Hsp90b1	Endoplasmin	0.010649298	−0.51	Response to ER stress and hypoxia
Rplp0	Large ribosomal subunit protein L10	0.033915975	−0.51	Translation, rRNA processing
Hnrnpf	Heterogeneous nuclear ribonucleoprotein F	0.012251364	−0.53	mRNA processing
Rpl12	Large ribosomal subunit protein L11	0.022757333	−0.53	Translation, ribosome biosynthesis
Rps3	Small ribosomal subunit protein S3	0.005865099	−0.57	Regulation of transcription and translation, cell cycle
H4c1	Histone H4	0.036585545	−0.77	Regulation of transcription, DNA repair and replication, regulation of chromosomal stability
Anxa5	Annexin A5	0.031302789	−0.82	Apoptosis, blood coagulation
Ppia	Peptidyl-prolyl cis-trans isomerase A	0.011540154	−0.80	Transcription, inflammation, apoptosis, protein folding
Capg	Macrophage-capping protein	0.016945742	−0.90	Capping of actin filaments, cell cycle
Cdk1	Cyclin-dependent kinase 1	0.027870605	−0.95	Cell cycle
Hmgb1	High mobility group protein B1	0.004082575	−0.97	Innate and acquired immunity, autophagy, chemotaxis, DNA damage, recombination and repair, immunity, inflammatory response
Arpc2	Actin-related protein 2/3 complex subunit 2	0.037879126	−0.97	Organization of actin filaments, filopodia and invadopodia
Rpl14	Large ribosomal subunit protein L14	0.032050689	−1.01	Translation, ribosome biogenesis
Pcna	Proliferating cell nuclear antigen	0.021224045	−1.02	DNA damage, repair and replication
Rpl3	Large ribosomal subunit protein L3	0.045588983	−1.11	Translation, ribosome biogenesis
Hnrnpd	Heterogeneous nuclear ribonucleoprotein D0	0.004487521	−1.23	Biological rhythms, transcription regulation

**TABLE 4:** List of differentially regulated proteins in AP and AG treated group vs. control group. Positive fold-change value denotes up-accumulation and negative fold change value denotes down-accumulation in comparison with control.

Gene symbol	Protein name	*p-*value (FDR)	Fold change (log2 vs*.* control)	Biological process
Map4	Microtubule-associated protein 4	0.04437854	0.64	Cell division, cytoskeleton organization
Ptma	Prothymosin alpha	0.016023377	0.52	Histone modification, negative regulation of apoptosis, immune response
Ipo7	Importin-7	0.048261411	0.46	Innate immune response, protein transport
Nolc1	Nucleolar and coiled-body phosphoprotein 1	0.011411515	0.46	Positive regulation of cell population proliferation, positive regulation of transcription, regulation of translation
Iqgap1	Ras GTPase-activating-like protein IQGAP1	0.040058222	0.45	Cytoskeleton organization, cell signaling
C3	Complement C3	0.020893975	0.40	Immune response, lipid metabolism
Npm1	Nucleophosmin	0.032210375	0.40	Ribosome biogenesis, cell proliferation
Sdpr	Caveolae-associated protein 2	0.021712332	0.38	Regulation of transcription, lipid metabolism
Hnrpf	Heterogeneous nuclear ribonucleoprotein F	0.012251364	0.37	mRNA processing, mRNA splicing
Mcm6	DNA replication licensing factor MCM6	0.0491043	0.35	Cell cycle, DNA replication
Hsp90b1	Endoplasmin	0.02374526	0.34	Response to ER stress and hypoxia
Cnn3	Calponin-3	0.010244988	0.34	Organization of the actin cytoskeleton
Pdia6	Protein disulfide-isomerase A6	0.036767114	0.30	Cellular redox homeostasis, stress response of unfolded proteins
Prdx6	Peroxiredoxin-6	0.014926228	0.29	Lipid catabolism, lipid metabolism, response to oxidative stress
Atp5pf	ATP synthase-coupling factor 6, mitochondrial	0.008585462	0.24	Mitochondrial proton transport associated with ATP synthesis
Cse1l	Exportin-2	0.011327294	0.24	Protein transport
P4hb	Protein disulfide-isomerase	0.011435107	0.23	Cellular redox homeostasis, cellular response to hypoxia, response to ER stress
Hspa5	Endoplasmic reticulum chaperone BiP	0.0285765	0.21	Protein folding, cellular response to stress, unfolded protein cellular response
Hspa8	Heat shock cognate 71 kDa protein	0.026478336	0.10	Stress response, mRNA processing
Fh1	Fumarate hydratase, mitochondrial	0.034636547	0.20	DNA damage, DNA repair, tricarboxylic acid cycle
Rab11b	Ras-related protein Rab-11B	0.027040956	0.19	Intracellular transport of proteins, organization of vesicles
Myl6	Myosin light polypeptide 6	0.011280272	−0.28	Muscle contraction, GTPase signaling
Actc1	Actin, alpha cardiac muscle 1	0.008895707	−0.84	Muscle contraction, negative regulation of apoptosis
Cox4i1	Cytochrome c oxidase subunit 4 isoform 1, mitochondrial	0.014751284	−0.94	Oxidative phosphorylation
Anxa1	Annexin A1	0.005184115	−1.01	Innate and acquired immunity, inflammatory response, proliferation, differentiation, apoptosis
Anxa5	Annexin A5	0.022359964	−1.22	Apoptosis, blood coagulation

**TABLE 5:** List of differentially regulated proteins in 5-FU treated group vs. control group. Positive fold-change value denotes up-accumulation and negative fold change value denotes down-accumulation in comparison with control.

Gene symbol	Protein name	*p-*value (FDR)	Fold change (log2 vs*.* control)	Biological process
Apoa2	Apolipoprotein A-II	0.004158	0.77	Lipid transport and metabolism, retinoid transport, chylomicron remodeling
Mt-1	Metallothionein-1	0.012015	0.67	Cellular homeostasis of metal ions, signal transduction mediated by nitric oxide
Ptma	Prothymosin alpha	0.016023	0.51	Histone modification, negative regulation of apoptosis, immune response
Ces1c	Carboxylesterase 1C	0.023659	0.45	Lipid catabolism, response to metal ions
Rpl29	Large ribosomal subunit protein L29	0.006359	0.42	Translation
Cavin2	Caveolae-associated protein 2	0.021712	0.40	Regulation of transcription, lipid metabolism
Cdk11b	Cyclin-dependent kinase 11B	0.013975	0.37	Cell cycle
Dnajc3	DnaJ homolog subfamily C member 3	0.013975	0.37	Reaction to stress, regulation of translation, stress of unfolded proteins
Ipo7	Importin-7	0.048261	0.31	Innate immune response, protein import into nucleus
Anxa5	Annexin A5	0.02236	−0.74	Calcium ion transmembrane transport, apoptosis
S100a9	Protein S100-A9	0.037099	−1.70	Apoptosis, autophagy, chemotaxis, immunity, inflammatory response, innate immunity

**FIGURE 3:**
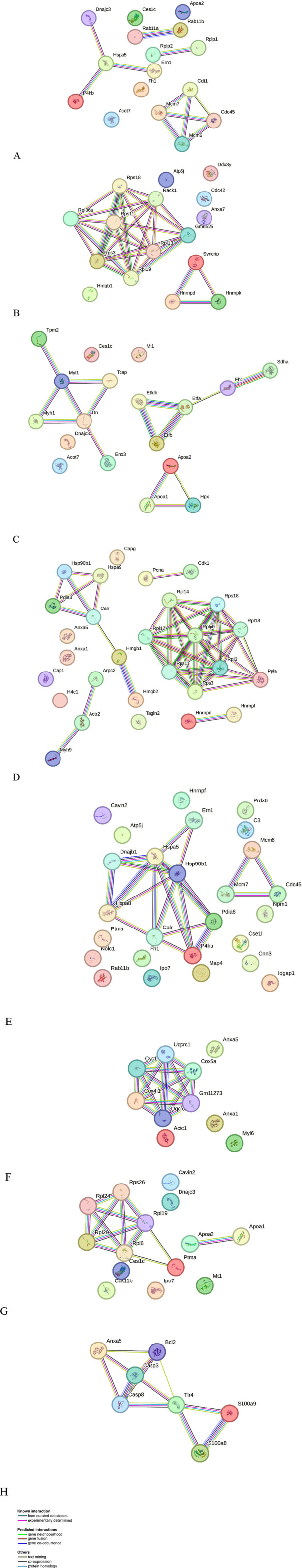
Protein-protein interaction networks identified using STRING for differential proteins in abundance. Different line colors represent the types of evidence for association. Gene names are shown. **(A)** AP group up-accumulated proteins, **(B)** AP group down-accumulated proteins, **(C)** combinatorial group (AP with 5-FU) group up-accumulated proteins, **(D)** combinatorial group (AP with 5-FU) down-accumulated proteins, **(E)** combinatorial group (AP with AG) up-accumulated proteins, and **(F)** combinatorial group (AP with AG) down-accumulated proteins, **(G)** 5-FU group up-accumulated proteins, **(H)** 5-FU group down-accumulated proteins.

In order to explore the likely mechanisms in more detail, protein-protein interaction network analysis was performed on the differentially regulated proteins using STRING, in accordance with previously set parameters. For each group, the up- and downregulated proteins were mapped to a different interaction network (Figure 3).

For the group treated with AP, important upregulated subnetworks include 4 proteins (DnaJ homolog subfamily C member 3 - DNAJC3, protein disulfide-isomerase - P4HB, endoplasmic reticulum chaperone BiP - HSPA5 and serine/threonine-protein kinase/endoribonuclease IRE1 - ERN1), of which DNAJC3, HSPA5 and ERN1 are involved in endoplasmic reticulum unfolded protein response (UPR). Proteins important in mitotic and nuclear DNA replication feature in a different subnetwork (DNA replication factor Cdt1 - CDT1, DNA replication licensing factor MCM7 - MCM7, DNA replication licensing factor MCM6 - MCM6 and cell division control protein 45 homolog - CDC45), cytosolic acyl coenzyme A thioester hydrolase (ACOT7) and apolipoprotein A-II (APOA2) are involved in lipid metabolism, while fumarate hydratase (FH1) is involved in citric acid cycle (TCA). The largest cluster of downregulated proteins in this group includes a number of ribosomal proteins (such as small ribosomal subunit protein uS3 - RPS3), important in ribosome biogenesis and eukaryotic translation. Also notable is a cluster of proteins involved in mRNA processing (heterogeneous nuclear ribonucleoprotein K - HNRNPK, heterogeneous nuclear ribonucleoprotein D0 - HNRNPD), while other downregulated proteins include cell division control protein 42 homolog (CDC42) (signal transduction and actin cytoskeleton reorganization), high mobility group protein B1 (HMGB1) (matrix metalloproteinase and immune system regulation) and ATP synthase-coupling factor 6, mitochondrial (ATP5J) (mitochondrial ATP synthesis). Upregulated proteins found in a group treated with AP + 5-FU have roles in lipid metabolism, such as fatty acid beta-oxidation (e.g., electron transfer flavoprotein subunit beta - ETFB, ACOT7, APOA2), citric acid cycle (FH1, succinate dehydrogenase [ubiquinone] flavoprotein subunit, mitochondrial - SDHA), and striated muscle contraction (tropomyosin beta chain - TPM2, myosin light chain 1/3 - MYL1). Similarly to the AP treated group, the largest cluster of downregulated proteins in the AP + 5-FU treated group is that of ribosomal proteins thereby indicating a role in eukaryotic translation, while other proteins are involved in functions such as positive regulation of DNA repair (high mobility group protein B1 - HMGB1, proliferating cell nuclear antigen - PCNA, actin-related protein 2 - ACTR2, RPS3), protein folding (HSPA90B1, protein disulfide-isomerase A3 - PDIA3, stress-70 protein - HSPA9, calreticulin - CALR, peptidyl-prolyl cis-trans isomerase A- PPIA) cytoskeleton organization (annexin A1 - ANXA1, adenylyl cyclase-associated protein 1 - CAP1, macrophage-capping protein - CAPG, ACTR2, myosin-9 - MYH9, RPS3), and mRNA stability (heterogeneous nuclear ribonucleoprotein F - HNRNPF, HNRNPD). The largest cluster of upregulated proteins in the AG + AP treated group includes proteins involved in protein folding and response to endoplasmic reticulum stress (DnaJ homolog subfamily B member 1 - DNAJB1, heat shock cognate 71 kDa protein - HSPA8, endoplasmic reticulum chaperone BiP - HSPA5, protein disulfide-isomerase A6 - PDIA6), while other important clusters feature proteins involved in double strand break repair (MCM6, MCM7, CDC45); both clusters are involved in cellular response to stress. Further upregulated proteins have functions in regulation of RNA splicing (HNRNPF, nucleophosmin - NPM1). In the AG + AP treated group, downregulated proteins form a cluster with functional significance in the process of respiratory electron transport (cytochrome c oxidase subunit 4 isoform 1, mitochondrial - COX4I1, cytochrome c1, heme protein, mitochondrial - CYC1), inflammatory and immune response (ANXA1) and apoptosis and regulated necrosis (annexin A5 **-** ANXA5). The upregulated proteins in the 5-FU treated groups are involved in cytoplasmic translation (a cluster of ribosomal proteins which includes large ribosomal subunit protein eL29- RPL29 and small ribosomal subunit protein eS26 - RPS26), response to endoplasmic reticulum stress (DNAJC3) and cholesterol homeostasis i.e., chylomicron remodeling (APOA2, carboxylesterase 1C- CES1C). Downregulated proteins in this group comprise a network of proteins involved in apoptosis and immune response (ANXA5, protein S100-A9 - S100A9).

In order to gain further insight into the biological significance of differentially regulated proteins, REACTOME pathway analysis was performed separately for upregulated and downregulated proteins in each group, according to previously established criteria (Supplementary Tables S1-S8).

### 3.3 Western blot

**FIGURE 4:**
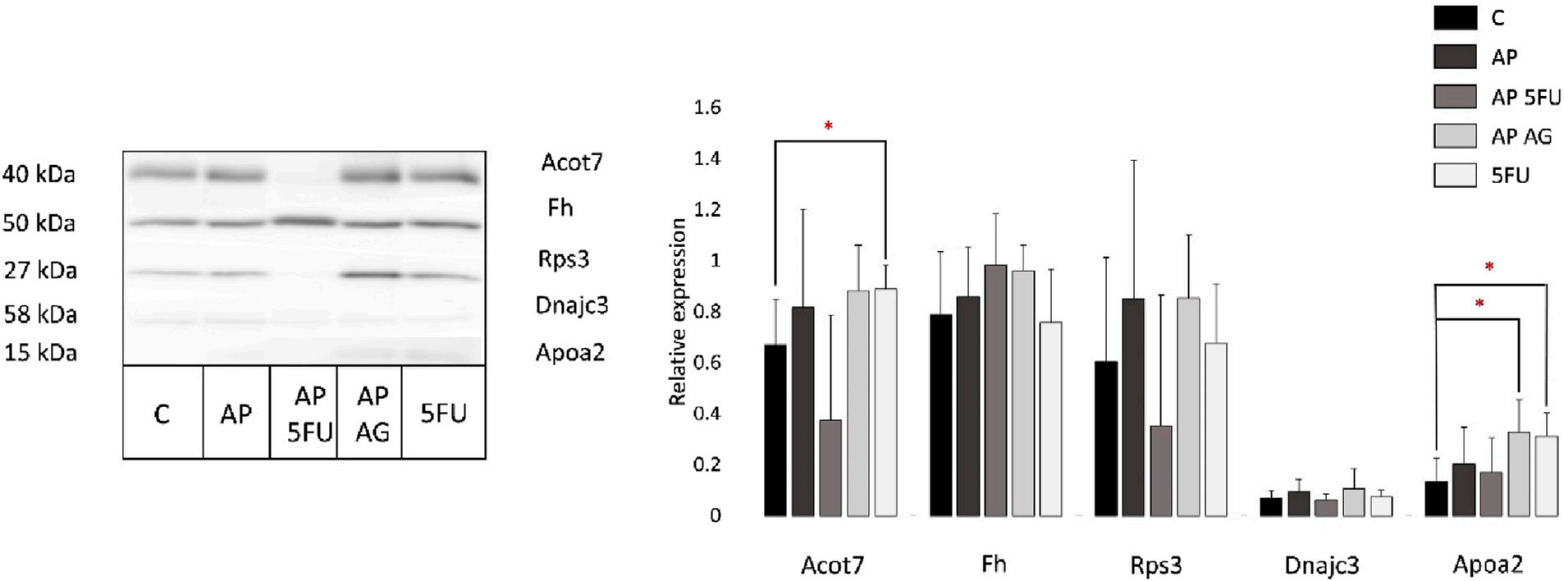
Representative Western blot and relative expression of Acot7, Fh, Rps3, Dnajc3, and Apoa2 in tumor tissues from control and four treated groups of animals (each group included tumors obtained from two mice). Results are presented as relative expression ±SEM measured in two biological replicates run in technical duplicates. Statistically significant changes (ANOVA, *p* < 0.05) are marked with an asterisk. C, control group of animals; AP, Agarikon Plus; AP + 5-FU, Agarikon Plus and 5-fluorouracil, AP + AG, Agarikon Plus and Agarikon.1; 5-FU, 5-fluorouracil.

As mass spectrometry results accompanied by the bioinformatics enrichment of identified proteins in analyzed tumor tissues revealed several deregulated molecular processes associated with disease progression (including lipid metabolism, tricarboxylic acid cycle, unfolded protein response [UPR] and the process of translation), in this study the relative expression levels of apolipoprotein A2 (APOA2), 40S ribosomal protein S3 (RPS3), acyl-CoA thioesterase 7 (ACOT7), fumarate hydratase (FH) and DnaJ homolog subfamily C member 3 (DNAJC3) were additionally validated by Western blot (Figure 4). APOA2 is known to be involved in high density lipoprotein (HDL) metabolism (Wang et al., 1997), while acyl-CoA thioesterase 7 (ACOT7) is important in unsaturated fatty acid biosynthesis which is involved in the initiation and progression of colon adenocarcinoma (COAD) (Chen et al., 2022). In accordance with the results of mass spectrometric profiling of tumor tissues, these proteins were shown to be mostly increased in comparison with control, while reaching statistical significance in certain instances (AP + AG and 5-FU vs. Control for APOA2; 5-FU vs. Control for ACOT7). Fumarate hydratase (FH) is a metabolic enzyme whose loss leads to tumorigenesis by various mechanisms (Schmidt et al., 2020), has also shown a tendency of increase in the experimental groups with comparison to control which is in line with proteomic data, but without statistical significance. DnaJ homolog subfamily C member 3 (DNAJC3) has an important role in the process of proteasomal degradation during UPR. Namely, it has been shown that P58IPK/DNAJC3 recruits Hsp70 to the cytosolic opening of the ER translocon thus stimulating its ATPase activity thereby assisting in the extraction of stalled nascent proteins into the cytoplasm for proteasomal degradation (Pearse and Hebert, 2006). Although not reaching statistical significance, Western blot analysis shows an increase in the DNAJC3 levels in the AP, AP + AG and 5-FU treated groups. Small ribosomal subunit protein S3 (RPS3) is a protein featuring in a large network of ribosomal proteins which functions in translation as well as DNA damage repair (Kim et al., 1995; Li et al., 2022). Western blot shows its levels to be elevated in AP, AP + AG, and 5-FU, while reduced in AP + 5-FU treated group, which is partially in accordance with the proteomic profiling results.

Since the results of Western blot are mostly aligned with proteomics data, but in certain instances without statistical significance where this significance was noted in the proteomics data, it is necessary to emphasize that the absence of statistical significance does not imply the absence of biological significance. This is mainly because the molecules with statistical significance and without statistical significance interact mutually in a molecular network system. Moreover, it has been shown that although hub molecules in the molecule network system play very important roles, their amount does not change much even when normal and diseased states are compared (Zhan et al., 2017).

## 4 Discussion

Translation, the process through which proteins are synthesized from an mRNA template, plays a vital role in cancer development and progression. This complex process in eukaryotes, influenced by cellular factors such as proliferation and nutrition, involves various translation factors and ribosomal proteins (Smith et al., 2021). Most targeted cancer therapies currently focus on signaling pathways. However, these pathways ultimately influence the control of translation machinery, a crucial and terminal aspect of signaling cascades, as noted in previous research (Parsyan, 2014). In particular, knowing about the fact that gene expression has a stochastic nature, regulation at the level of translation by RNA-binding proteins is highly relevant as these proteins may bear the ability to transduce small signals to cells and regulate its behavior or even fate (Swain et al., 2002; Dacheux et al., 2017; Dolcemascolo et al., 2022).

Alterations in the expression and activity of specific translation factors, particularly during cellular stresses like hypoxia and nutrient deprivation, are common in human cancers. These changes are associated with various types of cancers and different stages of disease and cellular transformation (Silvera et al., 2010). For instance, the MYC proto-oncogene protein significantly impacts translational control, reducing internal ribosome entry site (IRES)-dependent cyclin-dependent kinase 11 (CDK11) translation during mitosis and increasing chromosome instability (Barna et al., 2008). Additionally, hypoxia inhibits protein synthesis through a pathway involving eukaryotic translation initiation factor 4E-binding protein 1 (4E-BP1) and elongation factor 2 kinase, controlled by mTOR and uncoupled in breast cancer cells (Connolly et al., 2006). The RAS pathway is known to mediate the overexpression of the eukaryotic translation initiation factor eIF4E, an essential partner in tumorigenesis through increased translation (Lazaris-Karatzas et al., 1992). The translation factor eIF4E also cooperates with c-Myc in lymphomagenesis, and the interaction between AKT and eIF4E is significant in oncogenesis and cancer therapy (Bordeleau et al., 2008).

Beyond eukaryotic initiation factors (eIFs), the role of ribosomal proteins in tumor growth has been increasingly acknowledged. Ribosomal proteins are crucial for protein biosynthesis, with cytosolic ribosomes (cytoribosomes) and mitochondrial ribosomes (mitoribosomes) playing essential roles in cellular functions and stress responses. The biogenesis of these ribosomes includes ribosomal RNA processing and binding to ribosomal proteins, with alterations in this process occurring during tumorigenesis. Changes in the expression levels of cytosolic and mitochondrial ribosomal proteins in different types of tumors have been documented, and these proteins also exhibit extra-ribosomal functions related to cancer. Their role as potential biomarkers and molecular targets in cancer treatment is also being explored (Pecoraro et al., 2021).

Our data shows decreased levels of ribosomal proteins and heterogeneous ribonucleoprotein (hnRNP) clusters, associated with the processes of translation or mRNA processing, respectively, in the groups treated with AP and AP + 5-FU (Tables 2, 3; Figures 3A–D; Supplementary Tables S2, S4). Namely, downregulated pathways such as ribosome assembly (GO:0042255), translation (GO:0006412) and ribosome biogenesis (GO:0042254) indicate that these treatments counter the usual dynamics of the processes observed during CRC progression. It has been shown that the translation, influenza life cycle (R-HSA-168255) and mRNA splicing (GO:0008380) are the three most relevant pathways in a cluster for which the steady rise in protein abundance has been confirmed during CRC progression (Peng et al., 2016). This is in agreement with the processes reported in our previous research, which were likewise downregulated as a result of treatment with individual preparations from medicinal mushrooms (Agarikon.1) and its combinations with 5-fluorouracil (Jakopovic et al., 2020a). In particular, our result are in line with previous data of other authors on the role of RPs in colorectal cancer (El Khoury and Nasr, 2021).

We noted decreased levels of HMGB1 in the groups treated with AP and AP + 5-FU. HMGB1 is a nuclear protein that binds DNA, while in the extracellular environment it binds to the inflammatory receptor RAGE (receptor for Advanced Glycation End-products) and TLRs (Toll-like receptors). Binding to TLR4 leads to NF-κB upregulation and increased release of pro-inflammatory cytokines (Hreggvidsdóttir et al., 2012). We believe that this may indicate a potential reduction of the pro-inflammatory phenotype in the tumor microenvironment as a result of treatment. This possibility is further corroborated by its chemokine-like role and pro-inflammatory properties (Degryse and de Virgilio, 2003) that may be inhibited by cationic ribosomal proteins RPL9 in LPS + HMGB1-stimulated TNF-α expression in macrophage-like RAW264.7 cells (Watanabe et al., 2023) or in viral infections (Hu et al., 2023).

In the combination treatment AP + AG, however, we did not observe downregulation of the process of translation and/or mRNA processing, although they were recorded in individual treatments. This might be due to either different temporal activation of the aforementioned processes, where it is possible that in the group treated with AP + AG these processes occurred earlier, or the combination exerts a different mechanism of action. In both cases, better survival and smaller tumor mass was observed for combined AP + AG treatment in comparison to individual AG or AP treatments (Figures 1, 2; Table 1). Among downregulated proteins in the group treated with AP protein DDX3Y (ATP-dependent RNA helicase DDX3Y) and its interactors such as eukaryotic translation initiation factor 1A, X-chromosomal (EIF1AX) have roles in translation initiation (Supplementary Figure S2). CDC42 (cell division control protein 42 homolog), a small GTPase from the RHO family of small G proteins, which is also downregulated in this group, has important roles in the regulation of the cell cycle as well as cell morphology and migration by influencing actin cytoskeleton (Qadir et al., 2015). Its increased expression has been demonstrated in non-small cell lung cancer, colorectal cancer, melanoma, and breast and testicular cancer, which correlate with poorer survival (Stengel and Zheng, 2011). CDC42 has important roles in metastasis as it promotes β1 integrin expression (Wilson et al., 1987). This protein is an important therapeutic target in the treatment of CRC since it affects the transcription of a large number of genes important for disease progression (Valdés-Mora et al., 2017). ATP5J (ATP synthase-coupling factor 6, mitochondrial), which is among the downregulated proteins in the group treated with AP, is one of the components of the ATP synthase of the mitochondrial membrane, which participates in the process of oxidative phosphorylation (OXPHOS) (Figure 3B; Supplementary Figure S3). Also, in the group treated with AP + AG, we observed downregulation of proteins that are components of cytochrome c oxidase, that is, complex IV, the last enzyme necessary in the electron transport chain that initiates oxidative phosphorylation (Figure 3F). It has been demonstrated that the level of cytochrome oxidase, subunit IV, is often increased in CRC, and has possible effects in carcinogenesis, but not in CRC progression (Zhang et al., 2016). Metabolic reprogramming is one of the key features of tumors. In the research of potential therapeutics that affect this aspect of carcinogenesis, the process of glycolysis is mainly investigated. Since tumor cells have an increased level of glycolysis, this led to the assumption that OXPHOS is necessarily less active in tumors. Among the more recent findings, however, is that, for example, in tumors of the pancreas, endometrium, leukemia and lymphoma, OXPHOS can be a highly active process, even with an active process of glycolysis. It was recently proven that the small inhibitor of complex I of the mitochondrial electron transport chain IACS-010759, led to a strong inhibition of proliferation and induced apoptosis in a model of brain tumors and acute myeloid leukemia. This is believed to be the result of a lack of energy and reduced production of aspartate, which leads to impaired nucleotide synthesis (Molina et al., 2018).

Since the group treated with AP + 5-FU had the best survival compared to all other groups (OS = 87.5%, Table 1; Figure 1), it was interesting to determine potential differences in the proteomic profile compared to the other groups. Apart from the fact that in this group, as stated earlier, the level of ribosomal and heterogeneous ribonucleoproteins (hnRNP) was decreased, we noted a decreased level of cyclin-dependent kinase 1 (CDK1) in this group only (Figure 3D). CDK1 plays a key role in the progression of the cell cycle, specifically in the regulation of the G2-M checkpoint. With its serine/threonine kinase activity, CDK1 phosphorylates many different substrates, thereby promoting cell cycle progression (Enserink and Kolodner, 2010). As expected, increased expression of this protein correlates with worse prognosis in CRC patients, and therefore CDK1 is considered an important biomarker of this disease (Curtis et al., 2020; Li et al., 2020). The interaction of CDK1 with the proliferating cell nuclear antigen (PCNA), which is involved in the processes of DNA replication and repair, chromatin maintenance, chromosome separation and the cell cycle, is significant (Stoimenov and Helleday, 2009). Its significance as a potential biomarker is emphasized by the fact that its immunohistochemical expression progressively increases in the sequence of CRC progression (control-adenoma-carcinoma) (Qasim et al., 2012). Given that in this group, as well as in other treated groups, after treatment with AP + 5-FU, we observed decreased levels of arginase-1 (ARG1) and VEGF (Jakopovic et al., 2020b), a further bioinformatic analysis was performed in order to confirm functional interactions between CDK1 and VEGF, as well as ARG1 and other reduced proteins in this group, and the results are shown graphically in Supplementary Figure S4. We believe that the positive therapeutic effects recorded in this treatment (AP + 5-FU) are also a consequence of the processes associated with the upregulated proteins of this group. These processes include increased lipid metabolism, Krebs cycle (citric acid cycle or tricarboxylic acid cycle) and the unfolded protein response (UPR), which was also noted in some other therapeutic groups and will be described later (Figure 3C; Supplementary Table S3).

In the groups treated with AP as well as AP + AG, we observed increased levels of RAB11A and RAB11B (Ras-related protein RAB-11B), which belong to the family of small GTPases related to RAS, and have the function of regulating intracellular vesicular transport through the process of creation, binding and fusion of vesicles, as well as endo- and exocytosis (Tables 2, 4) (Wilcke et al., 2000). It was found that RAB11 proteins are often increased in CRC, where they correlate with a more pronounced metastasizing process in the lymph nodes in patients (Dong and Wu, 2018). RAB11B is recognized as a functional mediator of metastatic adaptation, because it participates in the recycling of proteins such as integrin β1 necessary for the interaction of tumor cells with the microenvironment (Howe et al., 2020). In the groups treated with AP and AP + AG, we also found an increased level of MCM6 (DNA replication licensing factor MCM6). The MCM group (mini-chromosome maintenance proteins) are DNA helicases that play significant roles in DNA replication and cell cycle progression, and due to their role in proliferation, they are primarily considered negative prognostic biomarkers (Gou et al., 2018). However, histochemical analysis of CRC in 619 patients found that MCM6 is actually a positive prognostic factor (Hendricks et al., 2019). Importin-7, noted to be upregulated in the AP + AG treated group, is involved in the nuclear import of ribosomal proteins and its elevation in cancer indicates a disruption in ribosome formation, leading to the activation of the tumor suppressor protein P53 (Golomb et al., 2012).

Besides its established role as a drug that primarily impacts replication and DNA integrity, 5-fluorouracil is also known to affect the protein composition of colorectal cancer cells. It reduces the presence of several ribosomal proteins and diminishes the cells’ ability to create proteins (Marin-Vicente et al., 2013). Contradictorily, our findings indicate an increase in protein synthesis pathways (GO:0006412) in cells treated with 5-fluorouracil (Figure 3G; Supplementary Table S7). This paradox may be explained by the drug’s swift impact on protein-making machinery, potentially triggering other functions outside of ribosomes, like the DNA-damage response (Yang et al., 2019). Earlier research also found that 5-FU treatment boosts the levels of certain ribosomal proteins not attached to ribosomes (L5, L11, L23) and their interaction with E3 ubiquitin-protein ligase Mdm2 (MDM2), which activates P53 and causes cell cycle arrest (Sun et al., 2007). Clinically, higher levels of certain ribosomal proteins (RPS and RPLs) were more common in patients who did not respond well to 5-fluorouracil treatment. These patients also had elevated levels of proteins like dihydropyrimidine dehydrogenase [NADP (+)] (DPYD) and thymidine phosphorylase (TYMP), involved in liver metabolism and the metabolic conversion of 5-FU, respectively. It is suggested that an increased presence of ribosomal and some mitochondrial proteins in the tumor proteomes of non-responding patients may lead to reduced effectiveness of 5-FU treatment and a possible new mechanism of resistance to the drug (Chauvin et al., 2018).

Prothymosin-α (PTMA), showing increased levels in patients treated with 5-fluorouracil, is associated with a negative prognosis in colorectal cancer. This protein is mainly involved in gene regulation through modifying chromatin structure (Zhang et al., 2014). Caveolins, known for their dual role in both hindering and promoting cancer depending on the cancer type and stage, are also notable. Their high expression is linked to the suppression of cancer-related pathways, including those related to growth factors (Gupta et al., 2014). In our previous study, CAVIN-2, also known as caveolae-associated protein, was found to be elevated as a result of treatment with both mushroom extract alone (Agarikon.1) and 5-fluorouracil treated groups (Jakopovic et al., 2020a). This protein, along with CAVIN-1, plays a role in inhibiting colorectal cancer progression and metastasis, with lower levels of CAVIN-1 indicating advanced disease stages.

Metallothionein-1 (MT1) is usually downregulated in CRC, so its upregulation as a result of 5-fluorouracil treatment could result in improved survival rates in colorectal cancer, potentially due to its role in promoting cancer cell differentiation (Arriaga et al., 2017). This protein was likewise upregulated in the AP + 5-FU treated group. Conversely, proteins like protein S100-A9 (S100A9), which is usually co-expressed with S100A8, were reduced in this group. These proteins are implicated in cancer development through the activation of MAPK and NF-κB pathways and are linked to various cancers, including colorectal cancer (Duan et al., 2013).

The analysis also showed that pathways associated with downregulated proteins are primarily involved in promoting apoptosis and regulating the immune response (Supplementary Table S8), suggesting an immunosuppressive effect of 5-fluorouracil in advanced cancer stages. This immunosuppressive impact, often seen as a severe side effect of 5-fluorouracil, especially in prolonged treatments, is primarily due to bone marrow suppression (Macdonald, 1999).

The unfolded protein response (UPR) is a fundamental cellular stress response mechanism associated with endoplasmic reticulum stress, playing a complex role in cancer by potentially both impeding and facilitating tumorigenesis. Short-term UPR signaling is known to reduce protein synthesis, increase chaperone production, and activate proteolysis (the ER-associated degradation system that removes misfolded proteins from the ER), whereas prolonged UPR signaling can lead to apoptosis (Jain, 2017). In the context of colorectal cancer, recent studies have shown that UPR activation leads to the differentiation of colon cancer stem cells, enhancing their response to chemotherapy both *in vitro* and *in vivo*. Activation of any of the primary UPR pathways [protein kinase R (PKR)-like endoplasmic reticulum kinase - PERK, activating transcription factor 6 - ATF6, or X-box binding protein 1 - XBP1] results in decreased cellular proliferation and reduced markers of intestinal epithelial stemness. Moreover, activation of IRE1-XBP1 and ATF6 pathways also reduces overall protein synthesis (Spaan et al., 2019). Research indicates that during colorectal cancer progression, the unfolded protein response tends to be progressively downregulated, aligning with these findings (Peng et al., 2016). Contrary to the trends observed during CRC progression, we observed increased levels of DnaJ homolog subfamily C member 3 (DNAJC3) and its associated proteins in all treatment groups, indicative of an elevated unfolded protein stress response (Supplementary Tables S1, S3, S5, S7). DNAJC3 upregulation is initiated by the inositol-requiring enzyme 1 (IRE1) arm of the UPR, with heat shock protein 90 (HSP90) necessary for maintaining the stability of IRE1α and PERK (Pearse and Hebert, 2006).

In all upregulated treatment groups in comparison to control, there was a notable enrichment in the regulation of lipid metabolism through peroxisome proliferator-activated receptor alpha (PPAR-α) (Supplementary Tables S1, S3, S5, S7). PPAR-α, a nuclear receptor, is pivotal in managing systemic lipid balance, cell growth and differentiation, energy metabolism, oxidative stress, inflammation, circadian rhythms, immune response, and cell differentiation (Qian et al., 2023). Fenofibrate, a PPAR-α agonist, is commonly used for hyperlipidemia treatment and has demonstrated potential anticancer effects, as it influences apoptosis, cell-cycle arrest, invasion, and migration in various cancer cell lines, including those of breast, liver, glioma, prostate, pancreas, and lung cancer (Lian et al., 2018). Furthermore, epidemiological studies have shown that naturally occurring dietary flavonoids such as quercetin (3,5,7,3,4-pentahydroxyflavone), kaempferol (3,5,7,4-tetrahydroxyflavone) and apigenin (4,5,7-trihydroxyflavone) also contribute in the activation process of PPAR-γ, thereby indicating that these compounds or their metabolites are also natural PPAR ligands (Ballav et al., 2022). 5-Fluorouracil (5-FU), a chemotherapeutic drug, also affects lipid metabolism in colorectal cancer cells. The resistance to 5-FU in colorectal cancer has been linked with alterations in sphingomyelin (SM) and ceramide (Cer) levels, controlled by acid sphingomyelinase (SMPD1). These changes in lipid composition are significant in the drug resistance mechanism, indicating the importance of lipid metabolism in the efficacy of 5-FU (Jung et al., 2020). Furthermore, research on mice colon tumors has shown that levels of PPAR-α mRNA are reduced in colon tumors compared to control tissues. Loss of PPAR-α enhances colon carcinogenesis, indicated by increases in DNA methyltransferase 1 (DNMT1) and protein arginine methyltransferase 6 (PRMT6) in colon tumors from PPAR-α-deficient mice. This finding emphasizes the role of PPAR-α in colon cancer prevention and treatment (Luo et al., 2019).

Activation of PPAR-α has been shown to promote fatty acid uptake, utilization, and catabolism through the upregulation of genes involved in fatty acid transport, binding, and activation, as well as peroxisomal and mitochondrial fatty acid β-oxidation. In a study involving KK mice 3T3-L1 adipocytes, PPARα activation by bezafibrate and GW7647, specific PPARα agonists, resulted in enhanced fatty acid oxidation and changes in gene expression related to both adipogenesis and fatty acid oxidation, highlighting its role in energy metabolism and potential application in cancer therapies (Goto et al., 2011).

In comparison to the control, a significant pathway enriched in all up-accumulated groups is the tricarboxylic acid (TCA) cycle, key for oxidative phosphorylation in cells (Supplementary Tables S1, S3, S5, S7). Normally, glucose is the primary pyruvate source for the TCA cycle in healthy cells, but cancer cells often divert glucose for anaerobic glycolysis, relying more on glutamine and fatty acids to supplement TCA cycle intermediates (Eagle, 1955). Fatty acid β-oxidation, generating acetyl-CoA for the TCA cycle, produces more ATP than carbohydrate oxidation (Koundouros and Poulogiannis, 2020). This shift, known as the Warburg effect, supports cancer cell proliferation and growth (Hanahan and Weinberg, 2011). Hypoxia-inducible factors HIFs activation in tumor environments also promotes anaerobic glycolysis, steering glucose away from the TCA cycle (Semenza, 2012). Wild-type TP53 in metabolism reduces glycolysis rates, favoring oxidative phosphorylation (Anderson et al., 2018). Additionally, indicators of TCA cycle activation include increased levels of fumarate hydratase (FH) in AP, AP + 5-FU, and AP + AG treated groups, and the electron transfer flavoprotein (ETF) cluster in the AP + 5-FU treated group, which is essential for oxidative phosphorylation (Figure 3C). FH deficiency, linked to increased cancer incidence, disrupts the TCA cycle, elevating glycolysis and glucose shunting into alternative pathways like lactate production and the pentose phosphate pathway. FH-deficient cells also enhance fatty acid synthesis and upregulate protein synthesis through mTOR activation. Increased fumarate and reduced iron in FH-deficient cells stabilize HIF-1α, boosting VEGF and glucose transporter 1 (GLUT1) expression. FH loss also triggers oncogenic processes such as EMT and epigenetic reprogramming, underscoring its role as a tumor suppressor (Schmidt et al., 2020). The TCA cycle’s involvement in colorectal cancer (CRC) progression is evidenced by the declining abundance of associated proteins through the adenoma-carcinoma sequence (Peng et al., 2016).

This research indicates that Agarikon Plus, a complex combination of safe medicinal mushroom species, along with the antimetabolite drug 5-FU, as well as Agarikon Plus and Agarikon.1, induce changes that counter colorectal cancer progression, and significantly improve survival rate in this cancer model. These antitumor effects seem to be linked to a shift in energy production pathways, notably increasing lipid metabolism and the tricarboxylic acid cycle (TCA), as well as enhancing the unfolded protein response (UPR), which are proven to be progressively downregulated during colorectal cancer progression. Through bioinformatic analysis, it was discovered that various downregulated proteins (DEPs) are associated with functions like ribosomal biogenesis, translation, influenza life cycle, and mRNA processing/splicing, all typically elevated in colorectal cancer (CRC) development. Given that the genes responsible for these processes are evolutionarily conserved, these findings encourage more focused research to develop new, scientifically substantiated biotherapies for cancer.

## Data Availability

The data presented in the study are deposited in the PRIDE repository, accession number PXD049261.
