# Supplementary material for: Proteomic study of medicinal mushroom extracts reveals antitumor mechanisms in an advanced colon cancer animal model via ribosomal biogenesis, translation, and metabolic pathways
Source: Front Pharmacol. 2024 Oct 18;15:1475102. doi: 10.3389/fphar.2024.1475102 (PMC11528127; doi:10.3389/fphar.2024.1475102)
Supplement: Supplementary file 1 [file Table1.docx]

**Supplementary tables**

**Supplementary table 1.** Enriched REACTOME pathways for up-accumulated proteins of the Agarikon Plus treated group

| Pathway identifier | Pathway name | #Entities found | #Entities total | #Interactors found | #Interactors total | Entities ratio | Entities pValue | Entities FDR |
| --- | --- | --- | --- | --- | --- | --- | --- | --- |
| R-HSA-8963888 | Chylomicron assembly | 7 | 14 | 2 | 51 | 9.12E-04 | 5.48E-10 | 3.52E-07 |
| R-HSA-8963898 | Plasma lipoprotein assembly | 7 | 30 | 2 | 89 | 0.001954 | 3.69E-08 | 1.17E-05 |
| R-HSA-8963899 | Plasma lipoprotein remodeling | 8 | 56 | 2 | 138 | 0.003647 | 5.48E-08 | 1.17E-05 |
| R-HSA-8963901 | Chylomicron remodeling | 6 | 17 | 1 | 66 | 0.001107 | 1.26E-07 | 2.02E-05 |
| R-HSA-8957275 | Post-translational protein phosphorylation | 6 | 109 | 0 | 0 | 0.007098 | 6.58E-07 | 8.42E-05 |
| R-HSA-975634 | Retinoid metabolism and transport | 6 | 79 | 2 | 72 | 0.005144 | 3.51E-06 | 3.19E-04 |
| R-HSA-174824 | Plasma lipoprotein assembly, remodeling, and clearance | 8 | 102 | 4 | 251 | 0.006642 | 4.31E-06 | 3.45E-04 |
| R-HSA-71403 | Citric acid cycle (TCA cycle) | 4 | 50 | 0 | 6 | 0.003256 | 1.70E-05 | 0.001195 |
| R-HSA-2187338 | Visual phototransduction | 7 | 169 | 2 | 133 | 0.011005 | 1.87E-05 | 0.001195 |
| R-HSA-68962 | Activation of the pre-replicative complex | 4 | 36 | 2 | 40 | 0.002344 | 3.93E-05 | 0.002082 |
| R-HSA-6806667 | Metabolism of fat-soluble vitamins | 6 | 89 | 2 | 165 | 0.005795 | 6.62E-05 | 0.003243 |
| R-HSA-381426 | Regulation of Insulin-like Growth Factor (IGF) transport and uptake by Insulin-like Growth Factor Binding Proteins (IGFBPs) | 6 | 127 | 0 | 134 | 0.00827 | 8.22E-05 | 0.003699 |
| R-HSA-176974 | Unwinding of DNA | 3 | 12 | 1 | 41 | 7.81E-04 | 3.21E-04 | 0.012836 |
| R-HSA-3000480 | Scavenging by Class A Receptors | 3 | 49 | 0 | 0 | 0.003191 | 3.62E-04 | 0.013401 |
| R-HSA-8964041 | LDL remodeling | 2 | 10 | 0 | 2 | 6.51E-04 | 5.34E-04 | 0.018685 |
| R-HSA-176187 | Activation of ATR in response to replication stress | 3 | 39 | 1 | 27 | 0.00254 | 7.15E-04 | 0.023601 |
| R-HSA-71406 | Pyruvate metabolism and Citric Acid (TCA) cycle | 4 | 100 | 1 | 65 | 0.006512 | 0.001054418 | 0.033741 |
| R-HSA-68949 | Orc1 removal from chromatin | 3 | 73 | 0 | 10 | 0.004754 | 0.001330101 | 0.034583 |
| R-HSA-69190 | DNA strand elongation | 3 | 38 | 1 | 47 | 0.002474 | 0.001330101 | 0.034583 |
| R-HSA-69205 | G1/S-Specific Transcription | 4 | 43 | 2 | 146 | 0.0028 | 0.00175526 | 0.043881 |
| R-HSA-8964058 | HDL remodeling | 3 | 24 | 2 | 73 | 0.001563 | 0.002270951 | 0.052938 |
| R-HSA-69206 | G1/S Transition | 6 | 150 | 5 | 381 | 0.009768 | 0.002301649 | 0.052938 |
| R-HSA-69239 | Synthesis of DNA | 4 | 132 | 4 | 100 | 0.008595 | 0.002775437 | 0.06106 |
| R-HSA-8964043 | Plasma lipoprotein clearance | 3 | 46 | 3 | 66 | 0.002995 | 0.003108033 | 0.065269 |
| R-HSA-69002 | DNA Replication Pre-Initiation | 4 | 131 | 4 | 130 | 0.00853 | 0.004397459 | 0.087949 |
| R-HSA-8964026 | Chylomicron clearance | 2 | 9 | 2 | 29 | 5.86E-04 | 0.004603457 | 0.092069 |
| R-HSA-192823 | Viral mRNA Translation | 3 | 114 | 0 | 23 | 0.007423 | 0.006528252 | 0.120339 |
| R-HSA-69052 | Switching of origins to a post-replicative state | 3 | 94 | 4 | 60 | 0.006121 | 0.006793917 | 0.120339 |
| R-HSA-3000471 | Scavenging by Class B Receptors | 2 | 21 | 1 | 24 | 0.001367 | 0.007078786 | 0.120339 |
| R-HSA-196854 | Metabolism of vitamins and cofactors | 6 | 385 | 2 | 272 | 0.02507 | 0.008030585 | 0.13652 |
| R-HSA-69306 | DNA Replication | 4 | 168 | 4 | 159 | 0.01094 | 0.009450894 | 0.151214 |
| R-HSA-453279 | Mitotic G1 phase and G1/S transition | 6 | 174 | 5 | 557 | 0.01133 | 0.010524579 | 0.168393 |
| R-HSA-8866423 | VLDL assembly | 2 | 9 | 0 | 50 | 5.86E-04 | 0.011491569 | 0.183865 |
| R-HSA-77289 | Mitochondrial Fatty Acid Beta-Oxidation | 3 | 105 | 0 | 76 | 0.006837 | 0.013687753 | 0.205316 |
| R-HSA-8874177 | ATF6B (ATF6-beta) activates chaperones | 1 | 4 | 0 | 1 | 2.60E-04 | 0.013778103 | 0.206672 |
| R-HSA-69481 | G2/M Checkpoints | 3 | 153 | 1 | 63 | 0.009963 | 0.018090841 | 0.253272 |
| R-HSA-8873719 | RAB geranylgeranylation | 2 | 68 | 0 | 15 | 0.004428 | 0.020061466 | 0.27727 |
| R-HSA-8963889 | Assembly of active LPL and LIPC lipase complexes | 2 | 30 | 0 | 52 | 0.001954 | 0.020542901 | 0.27727 |
| R-HSA-68867 | Assembly of the pre-replicative complex | 3 | 113 | 4 | 118 | 0.007358 | 0.021328491 | 0.27727 |
| R-HSA-5682113 | Defective ABCA1 causes TGD | 1 | 5 | 0 | 3 | 3.26E-04 | 0.021955109 | 0.285416 |
| R-HSA-70263 | Gluconeogenesis | 2 | 67 | 0 | 17 | 0.004363 | 0.023021793 | 0.299283 |
| R-HSA-2173782 | Binding and Uptake of Ligands by Scavenger Receptors | 3 | 168 | 1 | 63 | 0.01094 | 0.026011702 | 0.338152 |
| R-HSA-8964046 | VLDL clearance | 1 | 10 | 0 | 2 | 6.51E-04 | 0.032754075 | 0.393049 |
| R-HSA-156902 | Peptide chain elongation | 2 | 97 | 0 | 7 | 0.006316 | 0.034070476 | 0.408846 |
| R-HSA-72764 | Eukaryotic Translation Termination | 2 | 106 | 0 | 4 | 0.006902 | 0.037098562 | 0.435522 |
| R-HSA-72689 | Formation of a pool of free 40S subunits | 2 | 106 | 0 | 7 | 0.006902 | 0.038337886 | 0.435522 |
| R-HSA-9033241 | Peroxisomal protein import | 2 | 67 | 0 | 48 | 0.004363 | 0.039592949 | 0.435522 |
| R-HSA-68689 | CDC6 association with the ORC:origin complex | 0 | 8 | 1 | 7 | 5.21E-04 | 0.040776211 | 0.447665 |
| R-HSA-2408557 | Selenocysteine synthesis | 2 | 112 | 0 | 3 | 0.007293 | 0.040863545 | 0.447665 |
| R-HSA-69242 | S Phase | 4 | 179 | 5 | 344 | 0.011656 | 0.043359259 | 0.447665 |
| R-HSA-1799339 | SRP-dependent cotranslational protein targeting to membrane | 2 | 119 | 0 | 0 | 0.007749 | 0.043450519 | 0.447665 |
| R-HSA-975956 | Nonsense Mediated Decay (NMD) independent of the Exon Junction Complex (EJC) | 2 | 101 | 0 | 24 | 0.006577 | 0.044766493 | 0.447665 |
| R-HSA-3000497 | Scavenging by Class H Receptors | 1 | 15 | 0 | 2 | 9.77E-04 | 0.046087887 | 0.460879 |
| R-HSA-8854214 | TBC/RABGAPs | 2 | 51 | 1 | 81 | 0.003321 | 0.046768004 | 0.46768 |
| R-HSA-168273 | Influenza Viral RNA Transcription and Replication | 3 | 173 | 0 | 127 | 0.011265 | 0.046969779 | 0.469698 |
| R-HSA-156827 | L13a-mediated translational silencing of Ceruloplasmin expression | 2 | 120 | 0 | 5 | 0.007814 | 0.047442422 | 0.474424 |

**Supplementary table 2.** Enriched REACTOME pathways for down-accumulated proteins of the Agarikon Plus treated group

| Pathway identifier | Pathway name | #Entities found | #Entities total | #Interactors found | #Interactors total | Entities ratio | Entities pValue | Entities FDR |
| --- | --- | --- | --- | --- | --- | --- | --- | --- |
| R-HSA-72689 | Formation of a pool of free 40S subunits | 9 | 106 | 0 | 7 | 0.006902 | 2.41E-10 | 1.13E-07 |
| R-HSA-163210 | Formation of ATP by chemiosmotic coupling | 6 | 23 | 0 | 0 | 0.001498 | 2.82E-10 | 1.13E-07 |
| R-HSA-156827 | L13a-mediated translational silencing of Ceruloplasmin expression | 9 | 120 | 1 | 5 | 0.007814 | 6.75E-10 | 1.80E-07 |
| R-HSA-156902 | Peptide chain elongation | 8 | 97 | 0 | 7 | 0.006316 | 3.78E-09 | 7.57E-07 |
| R-HSA-72764 | Eukaryotic Translation Termination | 8 | 106 | 0 | 4 | 0.006902 | 5.43E-09 | 8.69E-07 |
| R-HSA-2408557 | Selenocysteine synthesis | 8 | 112 | 0 | 3 | 0.007293 | 8.20E-09 | 1.03E-06 |
| R-HSA-72706 | GTP hydrolysis and joining of the 60S ribosomal subunit | 9 | 120 | 2 | 61 | 0.007814 | 9.02E-09 | 1.03E-06 |
| R-HSA-1799339 | SRP-dependent cotranslational protein targeting to membrane | 8 | 119 | 0 | 0 | 0.007749 | 1.07E-08 | 1.07E-06 |
| R-HSA-975956 | Nonsense Mediated Decay (NMD) independent of the Exon Junction Complex (EJC) | 8 | 101 | 0 | 24 | 0.006577 | 1.21E-08 | 1.08E-06 |
| R-HSA-192823 | Viral mRNA Translation | 8 | 114 | 0 | 23 | 0.007423 | 2.96E-08 | 2.37E-06 |
| R-HSA-72737 | Cap-dependent Translation Initiation | 9 | 130 | 2 | 106 | 0.008465 | 9.07E-08 | 6.10E-06 |
| R-HSA-156842 | Eukaryotic Translation Elongation | 8 | 102 | 1 | 60 | 0.006642 | 9.24E-08 | 6.10E-06 |
| R-HSA-72613 | Eukaryotic Translation Initiation | 9 | 130 | 3 | 109 | 0.008465 | 1.01E-07 | 6.19E-06 |
| R-HSA-2408522 | Selenoamino acid metabolism | 8 | 180 | 0 | 11 | 0.011721 | 3.84E-07 | 2.19E-05 |
| R-HSA-927802 | Nonsense-Mediated Decay (NMD) | 8 | 124 | 1 | 100 | 0.008074 | 7.25E-07 | 3.63E-05 |
| R-HSA-975957 | Nonsense Mediated Decay (NMD) enhanced by the Exon Junction Complex (EJC) | 8 | 124 | 1 | 100 | 0.008074 | 7.25E-07 | 3.63E-05 |
| R-HSA-9010553 | Regulation of expression of SLITs and ROBOs | 8 | 183 | 1 | 100 | 0.011916 | 5.47E-06 | 2.57E-04 |
| R-HSA-168273 | Influenza Viral RNA Transcription and Replication | 8 | 173 | 0 | 127 | 0.011265 | 8.24E-06 | 3.63E-04 |
| R-HSA-9633012 | Response of EIF2AK4 (GCN2) to amino acid deficiency | 8 | 115 | 0 | 195 | 0.007488 | 1.16E-05 | 4.72E-04 |
| R-HSA-9013424 | RHOV GTPase cycle | 4 | 39 | 0 | 0 | 0.00254 | 1.18E-05 | 4.72E-04 |
| R-HSA-8949613 | Cristae formation | 6 | 31 | 0 | 143 | 0.002019 | 3.07E-05 | 0.001165 |
| R-HSA-72695 | Formation of the ternary complex, and subsequently, the 43S complex | 4 | 54 | 0 | 0 | 0.003516 | 4.17E-05 | 0.0015 |
| R-HSA-9711097 | Cellular response to starvation | 8 | 176 | 0 | 204 | 0.011461 | 4.99E-05 | 0.001698 |
| R-HSA-9013149 | RAC1 GTPase cycle | 6 | 191 | 0 | 0 | 0.012437 | 5.82E-05 | 0.001876 |
| R-HSA-9013409 | RHOJ GTPase cycle | 4 | 59 | 0 | 0 | 0.003842 | 5.86E-05 | 0.001876 |
| R-HSA-376176 | Signaling by ROBO receptors | 10 | 235 | 6 | 413 | 0.015302 | 6.35E-05 | 0.001904 |
| R-HSA-72702 | Ribosomal scanning and start codon recognition | 4 | 64 | 1 | 11 | 0.004167 | 1.19E-04 | 0.003454 |
| R-HSA-9013148 | CDC42 GTPase cycle | 5 | 159 | 0 | 0 | 0.010354 | 2.47E-04 | 0.006908 |
| R-HSA-5603041 | IRAK4 deficiency (TLR2/4) | 3 | 27 | 0 | 11 | 0.001758 | 2.88E-04 | 0.007607 |
| R-HSA-5663213 | RHO GTPases Activate WASPs and WAVEs | 4 | 41 | 5 | 57 | 0.00267 | 2.93E-04 | 0.007607 |
| R-HSA-72649 | Translation initiation complex formation | 4 | 62 | 0 | 45 | 0.004037 | 4.85E-04 | 0.012126 |
| R-HSA-168255 | Influenza Infection | 8 | 198 | 0 | 362 | 0.012893 | 5.74E-04 | 0.014063 |
| R-HSA-5602498 | MyD88 deficiency (TLR2/4) | 3 | 26 | 2 | 24 | 0.001693 | 5.86E-04 | 0.014063 |
| R-HSA-6791226 | Major pathway of rRNA processing in the nucleolus and cytosol | 8 | 189 | 3 | 410 | 0.012307 | 8.25E-04 | 0.018964 |
| R-HSA-8868773 | rRNA processing in the nucleus and cytosol | 8 | 207 | 3 | 412 | 0.013479 | 0.00100669 | 0.022147 |
| R-HSA-72766 | Translation | 9 | 339 | 4 | 415 | 0.022075 | 0.001009381 | 0.022206 |
| R-HSA-5686938 | Regulation of TLR by endogenous ligand | 3 | 36 | 1 | 25 | 0.002344 | 0.001255708 | 0.02637 |
| R-HSA-9754678 | SARS-CoV-2 modulates host translation machinery | 3 | 62 | 0 | 0 | 0.004037 | 0.00137863 | 0.028856 |
| R-HSA-9013406 | RHOQ GTPase cycle | 3 | 63 | 0 | 0 | 0.004102 | 0.001442824 | 0.028856 |
| R-HSA-1592230 | Mitochondrial biogenesis | 7 | 128 | 2 | 404 | 0.008335 | 0.002037643 | 0.040753 |
| R-HSA-72662 | Activation of the mRNA upon binding of the cap-binding complex and eIFs, and subsequent binding to 43S | 4 | 66 | 1 | 103 | 0.004298 | 0.002273558 | 0.043198 |
| R-HSA-163200 | Respiratory electron transport, ATP synthesis by chemiosmotic coupling, and heat production by uncoupling proteins. | 6 | 153 | 1 | 276 | 0.009963 | 0.002297127 | 0.043645 |
| R-HSA-72312 | rRNA processing | 8 | 247 | 3 | 471 | 0.016084 | 0.002642765 | 0.04757 |
| R-HSA-9735869 | SARS-CoV-1 modulates host translation machinery | 3 | 41 | 0 | 44 | 0.00267 | 0.003246127 | 0.056408 |
| R-HSA-9012999 | RHO GTPase cycle | 7 | 460 | 3 | 112 | 0.029954 | 0.003318097 | 0.056408 |
| R-HSA-2029480 | Fcgamma receptor (FCGR) dependent phagocytosis | 6 | 193 | 9 | 251 | 0.012568 | 0.003643252 | 0.061935 |
| R-HSA-2029482 | Regulation of actin dynamics for phagocytic cup formation | 5 | 158 | 8 | 173 | 0.010288 | 0.00501815 | 0.084222 |
| R-HSA-9013423 | RAC3 GTPase cycle | 3 | 100 | 0 | 0 | 0.006512 | 0.005263877 | 0.084222 |
| R-HSA-9013420 | RHOU GTPase cycle | 3 | 44 | 2 | 73 | 0.002865 | 0.00753328 | 0.120532 |
| R-HSA-9664417 | Leishmania phagocytosis | 4 | 157 | 6 | 78 | 0.010223 | 0.008035578 | 0.120534 |
| R-HSA-9664422 | FCGR3A-mediated phagocytosis | 4 | 157 | 6 | 78 | 0.010223 | 0.008035578 | 0.120534 |
| R-HSA-9664407 | Parasite infection | 4 | 157 | 6 | 78 | 0.010223 | 0.008035578 | 0.120534 |
| R-HSA-1428517 | The citric acid (TCA) cycle and respiratory electron transport | 6 | 238 | 2 | 333 | 0.015498 | 0.009831395 | 0.147471 |
| R-HSA-71291 | Metabolism of amino acids and derivatives | 8 | 667 | 0 | 271 | 0.043433 | 0.014816455 | 0.20743 |
| R-HSA-3928662 | EPHB-mediated forward signaling | 3 | 52 | 5 | 144 | 0.003386 | 0.026669672 | 0.373375 |
| R-HSA-5602358 | Diseases associated with the TLR signaling cascade | 3 | 42 | 3 | 163 | 0.002735 | 0.029715421 | 0.416016 |
| R-HSA-5260271 | Diseases of Immune System | 3 | 42 | 3 | 163 | 0.002735 | 0.029715421 | 0.416016 |
| R-HSA-109704 | PI3K Cascade | 2 | 58 | 0 | 24 | 0.003777 | 0.032706545 | 0.425185 |
| R-HSA-1268020 | Mitochondrial protein import | 2 | 69 | 0 | 15 | 0.004493 | 0.033444988 | 0.434785 |
| R-HSA-112399 | IRS-mediated signalling | 2 | 65 | 0 | 24 | 0.004233 | 0.038010747 | 0.456129 |
| R-HSA-5625970 | RHO GTPases activate KTN1 | 1 | 12 | 0 | 0 | 7.81E-04 | 0.040900328 | 0.470461 |
| R-HSA-428543 | Inactivation of CDC42 and RAC1 | 1 | 12 | 0 | 0 | 7.81E-04 | 0.040900328 | 0.470461 |
| R-HSA-2428928 | IRS-related events triggered by IGF1R | 2 | 69 | 0 | 30 | 0.004493 | 0.045275017 | 0.470461 |
| R-HSA-389359 | CD28 dependent Vav1 pathway | 2 | 17 | 3 | 83 | 0.001107 | 0.046111598 | 0.470461 |
| R-HSA-9013404 | RAC2 GTPase cycle | 2 | 92 | 1 | 7 | 0.005991 | 0.046111598 | 0.470461 |
| R-HSA-2428924 | IGF1R signaling cascade | 2 | 72 | 0 | 30 | 0.004688 | 0.047801832 | 0.470461 |
| R-HSA-2404192 | Signaling by Type 1 Insulin-like Growth Factor 1 Receptor (IGF1R) | 2 | 73 | 0 | 30 | 0.004754 | 0.048655388 | 0.470461 |

**Supplementary table 3.** Enriched REACTOME pathways for up-accumulated proteins of the group treated with Agarikon Plus and 5-fluorouracil

| Pathway identifier | Pathway name | #Entities found | #Entities total | #Interactors found | #Interactors total | Entities ratio | Entities pValue | Entities FDR |
| --- | --- | --- | --- | --- | --- | --- | --- | --- |
| R-HSA-8957275 | Post-translational protein phosphorylation | 5 | 109 | 0 | 0 | 0.007098 | 4.98E-06 | 0.001064 |
| R-HSA-390522 | Striated Muscle Contraction | 4 | 40 | 0 | 11 | 0.002605 | 5.84E-06 | 0.001064 |
| R-HSA-5660526 | Response to metal ions | 4 | 21 | 0 | 52 | 0.001367 | 2.12E-05 | 0.00257 |
| R-HSA-381426 | Regulation of Insulin-like Growth Factor (IGF) transport and uptake by Insulin-like Growth Factor Binding Proteins (IGFBPs) | 5 | 127 | 0 | 134 | 0.00827 | 2.83E-04 | 0.021973 |
| R-HSA-5661231 | Metallothioneins bind metals | 3 | 16 | 0 | 43 | 0.001042 | 3.01E-04 | 0.021973 |
| R-HSA-381183 | ATF6 (ATF6-alpha) activates chaperone genes | 4 | 15 | 4 | 210 | 9.77E-04 | 0.001531846 | 0.082321 |
| R-HSA-381033 | ATF6 (ATF6-alpha) activates chaperones | 4 | 17 | 4 | 210 | 0.001107 | 0.001583104 | 0.082321 |
| R-HSA-3371568 | Attenuation phase | 2 | 47 | 0 | 0 | 0.00306 | 0.005106504 | 0.217107 |
| R-HSA-3000480 | Scavenging by Class A Receptors | 2 | 49 | 0 | 0 | 0.003191 | 0.005534514 | 0.217107 |
| R-HSA-77289 | Mitochondrial Fatty Acid Beta-Oxidation | 3 | 105 | 0 | 76 | 0.006837 | 0.00769537 | 0.217107 |
| R-HSA-3371571 | HSF1-dependent transactivation | 2 | 59 | 0 | 0 | 0.003842 | 0.007910315 | 0.217107 |
| R-HSA-611105 | Respiratory electron transport | 4 | 118 | 0 | 276 | 0.007684 | 0.007930652 | 0.217107 |
| R-HSA-8963888 | Chylomicron assembly | 2 | 14 | 1 | 51 | 9.12E-04 | 0.008697926 | 0.217107 |
| R-HSA-1989781 | PPARA activates gene expression | 5 | 175 | 1 | 408 | 0.011395 | 0.009045941 | 0.217107 |
| R-HSA-114608 | Platelet degranulation | 3 | 141 | 3 | 50 | 0.009181 | 0.009046129 | 0.217107 |
| R-HSA-163200 | Respiratory electron transport, ATP synthesis by chemiosmotic coupling, and heat production by uncoupling proteins. | 4 | 153 | 0 | 276 | 0.009963 | 0.010966569 | 0.223368 |
| R-HSA-400206 | Regulation of lipid metabolism by PPARalpha | 5 | 177 | 1 | 443 | 0.011526 | 0.011040546 | 0.223368 |
| R-HSA-8874177 | ATF6B (ATF6-beta) activates chaperones | 1 | 4 | 0 | 1 | 2.60E-04 | 0.011168419 | 0.223368 |
| R-HSA-397014 | Muscle contraction | 4 | 232 | 1 | 188 | 0.015107 | 0.013431763 | 0.253357 |
| R-HSA-8963901 | Chylomicron remodeling | 2 | 17 | 1 | 66 | 0.001107 | 0.014787643 | 0.253357 |
| R-HSA-2173782 | Binding and Uptake of Ligands by Scavenger Receptors | 3 | 168 | 0 | 63 | 0.01094 | 0.014903351 | 0.253357 |
| R-HSA-8953897 | Cellular responses to stimuli | 15 | 1051 | 7 | 3011 | 0.068438 | 0.017249157 | 0.26716 |
| R-HSA-5682113 | Defective ABCA1 causes TGD | 1 | 5 | 0 | 3 | 3.26E-04 | 0.017810675 | 0.26716 |
| R-HSA-381119 | Unfolded Protein Response (UPR) | 8 | 156 | 5 | 1370 | 0.010158 | 0.018196334 | 0.272945 |
| R-HSA-76005 | Response to elevated platelet cytosolic Ca2+ | 3 | 148 | 3 | 113 | 0.009637 | 0.020372479 | 0.285215 |
| R-HSA-3371453 | Regulation of HSF1-mediated heat shock response | 3 | 113 | 0 | 155 | 0.007358 | 0.020785256 | 0.290994 |
| R-HSA-9033241 | Peroxisomal protein import | 2 | 67 | 0 | 48 | 0.004363 | 0.026874739 | 0.344213 |
| R-HSA-8963898 | Plasma lipoprotein assembly | 2 | 30 | 1 | 89 | 0.001954 | 0.027755881 | 0.344213 |
| R-HSA-2187338 | Visual phototransduction | 3 | 169 | 1 | 133 | 0.011005 | 0.029004207 | 0.344213 |
| R-HSA-1428517 | The citric acid (TCA) cycle and respiratory electron transport | 4 | 238 | 0 | 333 | 0.015498 | 0.029662594 | 0.344213 |
| R-HSA-3371556 | Cellular response to heat stress | 3 | 135 | 0 | 183 | 0.008791 | 0.031292125 | 0.344213 |
| R-HSA-2168880 | Scavenging of heme from plasma | 2 | 106 | 0 | 18 | 0.006902 | 0.031395062 | 0.345346 |
| R-HSA-5660489 | MTF1 activates gene expression | 1 | 6 | 0 | 10 | 3.91E-04 | 0.03531024 | 0.388413 |
| R-HSA-975634 | Retinoid metabolism and transport | 2 | 79 | 1 | 72 | 0.005144 | 0.042823347 | 0.428233 |

**Supplementary table 4.** Enriched REACTOME pathways for down-accumulated proteins of the group treated with Agarikon Plus and 5-fluorouracil

| Pathway identifier | Pathway name | #Entities found | #Entities total | #Interactors found | #Interactors total | Entities ratio | Entities pValue | Entities FDR |
| --- | --- | --- | --- | --- | --- | --- | --- | --- |
| R-HSA-156902 | Peptide chain elongation | 10 | 97 | 0 | 7 | 0.006316 | 1.05E-10 | 6.73E-08 |
| R-HSA-72764 | Eukaryotic Translation Termination | 10 | 106 | 0 | 4 | 0.006902 | 1.64E-10 | 6.73E-08 |
| R-HSA-72689 | Formation of a pool of free 40S subunits | 10 | 106 | 0 | 7 | 0.006902 | 1.95E-10 | 6.73E-08 |
| R-HSA-2408557 | Selenocysteine synthesis | 10 | 112 | 0 | 3 | 0.007293 | 2.74E-10 | 7.09E-08 |
| R-HSA-1799339 | SRP-dependent cotranslational protein targeting to membrane | 10 | 119 | 0 | 0 | 0.007749 | 3.79E-10 | 7.65E-08 |
| R-HSA-975956 | Nonsense Mediated Decay (NMD) independent of the Exon Junction Complex (EJC) | 10 | 101 | 0 | 24 | 0.006577 | 4.45E-10 | 7.65E-08 |
| R-HSA-156827 | L13a-mediated translational silencing of Ceruloplasmin expression | 10 | 120 | 0 | 5 | 0.007814 | 6.06E-10 | 8.97E-08 |
| R-HSA-192823 | Viral mRNA Translation | 10 | 114 | 0 | 23 | 0.007423 | 1.35E-09 | 1.74E-07 |
| R-HSA-156842 | Eukaryotic Translation Elongation | 10 | 102 | 1 | 60 | 0.006642 | 5.54E-09 | 6.37E-07 |
| R-HSA-72706 | GTP hydrolysis and joining of the 60S ribosomal subunit | 10 | 120 | 1 | 61 | 0.007814 | 1.04E-08 | 1.07E-06 |
| R-HSA-2408522 | Selenoamino acid metabolism | 10 | 180 | 0 | 11 | 0.011721 | 3.24E-08 | 3.05E-06 |
| R-HSA-927802 | Nonsense-Mediated Decay (NMD) | 10 | 124 | 1 | 100 | 0.008074 | 7.12E-08 | 5.62E-06 |
| R-HSA-975957 | Nonsense Mediated Decay (NMD) enhanced by the Exon Junction Complex (EJC) | 10 | 124 | 1 | 100 | 0.008074 | 7.12E-08 | 5.62E-06 |
| R-HSA-72737 | Cap-dependent Translation Initiation | 10 | 130 | 1 | 106 | 0.008465 | 1.29E-07 | 9.56E-06 |
| R-HSA-72613 | Eukaryotic Translation Initiation | 10 | 130 | 1 | 109 | 0.008465 | 1.46E-07 | 1.01E-05 |
| R-HSA-9010553 | Regulation of expression of SLITs and ROBOs | 10 | 183 | 2 | 100 | 0.011916 | 8.64E-07 | 5.53E-05 |
| R-HSA-168273 | Influenza Viral RNA Transcription and Replication | 10 | 173 | 0 | 127 | 0.011265 | 1.43E-06 | 8.59E-05 |
| R-HSA-444473 | Formyl peptide receptors bind formyl peptides and many other ligands | 4 | 11 | 0 | 7 | 7.16E-04 | 1.58E-06 | 9.02E-05 |
| R-HSA-9633012 | Response of EIF2AK4 (GCN2) to amino acid deficiency | 10 | 115 | 1 | 195 | 0.007488 | 2.17E-06 | 1.17E-04 |
| R-HSA-8950505 | Gene and protein expression by JAK-STAT signaling after Interleukin-12 stimulation | 12 | 73 | 8 | 470 | 0.004754 | 1.02E-05 | 5.21E-04 |
| R-HSA-9711097 | Cellular response to starvation | 10 | 176 | 1 | 204 | 0.011461 | 1.31E-05 | 6.42E-04 |
| R-HSA-6798695 | Neutrophil degranulation | 11 | 478 | 0 | 0 | 0.031126 | 1.90E-05 | 8.93E-04 |
| R-HSA-3214815 | HDACs deacetylate histones | 5 | 63 | 0 | 10 | 0.004102 | 2.91E-05 | 0.001308 |
| R-HSA-73728 | RNA Polymerase I Promoter Opening | 4 | 33 | 0 | 4 | 0.002149 | 3.30E-05 | 0.001419 |
| R-HSA-110330 | Recognition and association of DNA glycosylase with site containing an affected purine | 4 | 38 | 0 | 2 | 0.002474 | 4.46E-05 | 0.001774 |
| R-HSA-376176 | Signaling by ROBO receptors | 12 | 235 | 3 | 413 | 0.015302 | 4.55E-05 | 0.001774 |
| R-HSA-9020591 | Interleukin-12 signaling | 12 | 84 | 8 | 561 | 0.00547 | 5.06E-05 | 0.001922 |
| R-HSA-168255 | Influenza Infection | 11 | 198 | 2 | 362 | 0.012893 | 5.42E-05 | 0.001956 |
| R-HSA-2514853 | Condensation of Prometaphase Chromosomes | 3 | 15 | 0 | 0 | 9.77E-04 | 5.59E-05 | 0.001956 |
| R-HSA-212300 | PRC2 methylates histones and DNA | 4 | 43 | 0 | 0 | 0.0028 | 5.89E-05 | 0.002002 |
| R-HSA-110331 | Cleavage of the damaged purine | 4 | 44 | 0 | 0 | 0.002865 | 6.43E-05 | 0.002123 |
| R-HSA-140342 | Apoptosis induced DNA fragmentation | 3 | 13 | 0 | 6 | 8.47E-04 | 8.08E-05 | 0.002568 |
| R-HSA-73927 | Depurination | 4 | 45 | 0 | 2 | 0.00293 | 8.28E-05 | 0.002568 |
| R-HSA-110329 | Cleavage of the damaged pyrimidine | 4 | 50 | 0 | 0 | 0.003256 | 1.05E-04 | 0.003149 |
| R-HSA-9710421 | Defective pyroptosis | 4 | 51 | 0 | 0 | 0.003321 | 1.13E-04 | 0.003283 |
| R-HSA-606279 | Deposition of new CENPA-containing nucleosomes at the centromere | 4 | 53 | 0 | 0 | 0.003451 | 1.31E-04 | 0.00367 |
| R-HSA-774815 | Nucleosome assembly | 4 | 53 | 0 | 0 | 0.003451 | 1.31E-04 | 0.00367 |
| R-HSA-5334118 | DNA methylation | 4 | 35 | 1 | 27 | 0.002279 | 1.85E-04 | 0.004799 |
| R-HSA-68616 | Assembly of the ORC complex at the origin of replication | 4 | 39 | 0 | 25 | 0.00254 | 1.85E-04 | 0.004799 |
| R-HSA-73772 | RNA Polymerase I Promoter Escape | 4 | 60 | 0 | 0 | 0.003907 | 2.10E-04 | 0.005246 |
| R-HSA-114608 | Platelet degranulation | 6 | 141 | 1 | 50 | 0.009181 | 3.17E-04 | 0.007926 |
| R-HSA-171306 | Packaging Of Telomere Ends | 4 | 33 | 1 | 41 | 0.002149 | 3.75E-04 | 0.008998 |
| R-HSA-6791226 | Major pathway of rRNA processing in the nucleolus and cytosol | 10 | 189 | 2 | 410 | 0.012307 | 3.99E-04 | 0.009581 |
| R-HSA-447115 | Interleukin-12 family signaling | 12 | 96 | 9 | 720 | 0.006251 | 4.37E-04 | 0.010053 |
| R-HSA-9821002 | Chromatin modifications during the maternal to zygotic transition (MZT) | 4 | 48 | 0 | 25 | 0.003126 | 4.39E-04 | 0.010088 |
| R-HSA-8868773 | rRNA processing in the nucleus and cytosol | 10 | 207 | 2 | 412 | 0.013479 | 5.08E-04 | 0.01118 |
| R-HSA-110328 | Recognition and association of DNA glycosylase with site containing an affected pyrimidine | 4 | 41 | 0 | 35 | 0.00267 | 5.10E-04 | 0.011214 |
| R-HSA-73928 | Depyrimidination | 4 | 50 | 0 | 35 | 0.003256 | 7.72E-04 | 0.01621 |
| R-HSA-5663213 | RHO GTPases Activate WASPs and WAVEs | 4 | 41 | 0 | 57 | 0.00267 | 9.53E-04 | 0.020008 |
| R-HSA-5578749 | Transcriptional regulation by small RNAs | 4 | 80 | 0 | 20 | 0.005209 | 0.00130117 | 0.026023 |
| R-HSA-73929 | Base-Excision Repair, AP Site Formation | 4 | 62 | 0 | 37 | 0.004037 | 0.001350201 | 0.027004 |
| R-HSA-5689901 | Metalloprotease DUBs | 4 | 32 | 0 | 74 | 0.002084 | 0.001505025 | 0.028595 |
| R-HSA-76005 | Response to elevated platelet cytosolic Ca2+ | 6 | 148 | 1 | 113 | 0.009637 | 0.001534126 | 0.029148 |
| R-HSA-72312 | rRNA processing | 10 | 247 | 2 | 471 | 0.016084 | 0.001624429 | 0.030864 |
| R-HSA-3928662 | EPHB-mediated forward signaling | 5 | 52 | 1 | 144 | 0.003386 | 0.001952141 | 0.035139 |
| R-HSA-72695 | Formation of the ternary complex, and subsequently, the 43S complex | 3 | 54 | 0 | 0 | 0.003516 | 0.002279133 | 0.041024 |
| R-HSA-72766 | Translation | 10 | 339 | 2 | 415 | 0.022075 | 0.002652024 | 0.04644 |
| R-HSA-73884 | Base Excision Repair | 5 | 99 | 1 | 102 | 0.006447 | 0.002731755 | 0.04644 |
| R-HSA-5689603 | UCH proteinases | 5 | 98 | 1 | 155 | 0.006381 | 0.002911307 | 0.049492 |
| R-HSA-427389 | ERCC6 (CSB) and EHMT2 (G9a) positively regulate rRNA expression | 4 | 47 | 1 | 89 | 0.00306 | 0.003036085 | 0.049983 |
| R-HSA-9662360 | Sensory processing of sound by inner hair cells of the cochlea | 4 | 76 | 0 | 49 | 0.004949 | 0.003123927 | 0.049983 |
| R-HSA-2299718 | Condensation of Prophase Chromosomes | 5 | 54 | 2 | 157 | 0.003516 | 0.003229318 | 0.051669 |
| R-HSA-9754678 | SARS-CoV-2 modulates host translation machinery | 3 | 62 | 0 | 0 | 0.004037 | 0.003356259 | 0.051724 |
| R-HSA-196025 | Formation of annular gap junctions | 2 | 11 | 0 | 7 | 7.16E-04 | 0.003433627 | 0.051724 |
| R-HSA-3214847 | HATs acetylate histones | 6 | 110 | 2 | 209 | 0.007163 | 0.003448259 | 0.051724 |
| R-HSA-2559582 | Senescence-Associated Secretory Phenotype (SASP) | 4 | 90 | 0 | 45 | 0.005861 | 0.003889945 | 0.058349 |
| R-HSA-2029482 | Regulation of actin dynamics for phagocytic cup formation | 6 | 158 | 2 | 173 | 0.010288 | 0.004156703 | 0.062351 |
| R-HSA-9659379 | Sensory processing of sound | 4 | 87 | 0 | 49 | 0.005665 | 0.004207262 | 0.063109 |
| R-HSA-9664417 | Leishmania phagocytosis | 5 | 157 | 0 | 78 | 0.010223 | 0.004582935 | 0.064161 |
| R-HSA-9664422 | FCGR3A-mediated phagocytosis | 5 | 157 | 0 | 78 | 0.010223 | 0.004582935 | 0.064161 |
| R-HSA-9664407 | Parasite infection | 5 | 157 | 0 | 78 | 0.010223 | 0.004582935 | 0.064161 |
| R-HSA-72702 | Ribosomal scanning and start codon recognition | 3 | 64 | 0 | 11 | 0.004167 | 0.004887459 | 0.068424 |

**Supplementary table 5.** Enriched REACTOME pathways for up-accumulated proteins of the group treated with Agarikon Plus and Agarikon.1

| Pathway identifier | Pathway name | #Entities found | #Entities total | #Interactors found | #Interactors total | Entities ratio | Entities pValue | Entities FDR |
| --- | --- | --- | --- | --- | --- | --- | --- | --- |
| R-HSA-163210 | Formation of ATP by chemiosmotic coupling | 6 | 23 | 0 | 0 | 0.001498 | 4.45E-08 | 5.71E-05 |
| R-HSA-5626467 | RHO GTPases activate IQGAPs | 6 | 36 | 0 | 11 | 0.002344 | 2.44E-06 | 0.001568 |
| R-HSA-174403 | Glutathione synthesis and recycling | 5 | 27 | 0 | 5 | 0.001758 | 7.30E-06 | 0.003126 |
| R-HSA-140342 | Apoptosis induced DNA fragmentation | 4 | 13 | 0 | 6 | 8.47E-04 | 1.28E-05 | 0.004097 |
| R-HSA-156590 | Glutathione conjugation | 6 | 68 | 0 | 8 | 0.004428 | 4.07E-05 | 0.010422 |
| R-HSA-9753281 | Paracetamol ADME | 5 | 63 | 0 | 0 | 0.004102 | 1.77E-04 | 0.03781 |
| R-HSA-5423646 | Aflatoxin activation and detoxification | 4 | 49 | 0 | 1 | 0.003191 | 7.77E-04 | 0.14217 |
| R-HSA-71403 | Citric acid cycle (TCA cycle) | 4 | 50 | 0 | 6 | 0.003256 | 0.001030938 | 0.16495 |
| R-HSA-6798695 | Neutrophil degranulation | 11 | 478 | 0 | 0 | 0.031126 | 0.001876283 | 0.253599 |
| R-HSA-9636249 | Inhibition of nitric oxide production | 2 | 5 | 0 | 3 | 3.26E-04 | 0.001981238 | 0.253599 |
| R-HSA-8949613 | Cristae formation | 6 | 31 | 1 | 143 | 0.002019 | 0.002745245 | 0.318448 |
| R-HSA-163200 | Respiratory electron transport, ATP synthesis by chemiosmotic coupling, and heat production by uncoupling proteins. | 9 | 153 | 2 | 276 | 0.009963 | 0.004319083 | 0.353132 |
| R-HSA-5625970 | RHO GTPases activate KTN1 | 2 | 12 | 0 | 0 | 7.81E-04 | 0.00436462 | 0.353132 |
| R-HSA-9700645 | ALK mutants bind TKIs | 2 | 12 | 0 | 0 | 7.81E-04 | 0.00436462 | 0.353132 |
| R-HSA-428543 | Inactivation of CDC42 and RAC1 | 2 | 12 | 0 | 0 | 7.81E-04 | 0.00436462 | 0.353132 |
| R-HSA-6790901 | rRNA modification in the nucleus and cytosol | 4 | 71 | 0 | 10 | 0.004623 | 0.004414155 | 0.353132 |
| R-HSA-9664535 | LTC4-CYSLTR mediated IL4 production | 2 | 14 | 0 | 0 | 9.12E-04 | 0.005878445 | 0.353187 |
| R-HSA-168276 | NS1 Mediated Effects on Host Pathways | 4 | 46 | 1 | 45 | 0.002995 | 0.006364168 | 0.353187 |
| R-HSA-176974 | Unwinding of DNA | 3 | 12 | 1 | 41 | 7.81E-04 | 0.006754075 | 0.353187 |
| R-HSA-3371568 | Attenuation phase | 3 | 47 | 0 | 0 | 0.00306 | 0.006754075 | 0.353187 |
| R-HSA-9700649 | Drug resistance of ALK mutants | 1 | 1 | 0 | 0 | 6.51E-05 | 0.008026977 | 0.353187 |
| R-HSA-9717326 | crizotinib-resistant ALK mutants | 1 | 1 | 0 | 0 | 6.51E-05 | 0.008026977 | 0.353187 |
| R-HSA-9717316 | alectinib-resistant ALK mutants | 1 | 1 | 0 | 0 | 6.51E-05 | 0.008026977 | 0.353187 |
| R-HSA-9717264 | ASP-3026-resistant ALK mutants | 1 | 1 | 0 | 0 | 6.51E-05 | 0.008026977 | 0.353187 |
| R-HSA-9717301 | NVP-TAE684-resistant ALK mutants | 1 | 1 | 0 | 0 | 6.51E-05 | 0.008026977 | 0.353187 |
| R-HSA-9717329 | lorlatinib-resistant ALK mutants | 1 | 1 | 0 | 0 | 6.51E-05 | 0.008026977 | 0.353187 |
| R-HSA-9717319 | brigatinib-resistant ALK mutants | 1 | 1 | 0 | 0 | 6.51E-05 | 0.008026977 | 0.353187 |
| R-HSA-9717323 | ceritinib-resistant ALK mutants | 1 | 1 | 0 | 0 | 6.51E-05 | 0.008026977 | 0.353187 |
| R-HSA-381183 | ATF6 (ATF6-alpha) activates chaperone genes | 6 | 15 | 4 | 210 | 9.77E-04 | 0.009306525 | 0.390874 |
| R-HSA-381033 | ATF6 (ATF6-alpha) activates chaperones | 6 | 17 | 4 | 210 | 0.001107 | 0.009698979 | 0.397658 |
| R-HSA-1428517 | The citric acid (TCA) cycle and respiratory electron transport | 10 | 238 | 3 | 333 | 0.015498 | 0.010320128 | 0.412805 |
| R-HSA-977606 | Regulation of Complement cascade | 6 | 139 | 7 | 109 | 0.009051 | 0.012070837 | 0.436045 |
| R-HSA-8957275 | Post-translational protein phosphorylation | 4 | 109 | 0 | 0 | 0.007098 | 0.012181964 | 0.436045 |
| R-HSA-168255 | Influenza Infection | 10 | 198 | 10 | 362 | 0.012893 | 0.012375051 | 0.436045 |
| R-HSA-3371571 | HSF1-dependent transactivation | 3 | 59 | 0 | 0 | 0.003842 | 0.012458426 | 0.436045 |
| R-HSA-69002 | DNA Replication Pre-Initiation | 6 | 131 | 4 | 130 | 0.00853 | 0.013273569 | 0.451301 |
| R-HSA-173736 | Alternative complement activation | 2 | 6 | 2 | 16 | 3.91E-04 | 0.013918773 | 0.454636 |
| R-HSA-176187 | Activation of ATR in response to replication stress | 3 | 39 | 2 | 27 | 0.00254 | 0.014207375 | 0.454636 |
| R-HSA-9013406 | RHOQ GTPase cycle | 3 | 63 | 0 | 0 | 0.004102 | 0.01481971 | 0.474231 |
| R-HSA-9645722 | Defective Intrinsic Pathway for Apoptosis Due to p14ARF Loss of Function | 1 | 2 | 0 | 0 | 1.30E-04 | 0.01598987 | 0.495686 |
| R-HSA-2559585 | Oncogene Induced Senescence | 5 | 42 | 5 | 157 | 0.002735 | 0.017315733 | 0.505089 |
| R-HSA-68962 | Activation of the pre-replicative complex | 3 | 36 | 2 | 40 | 0.002344 | 0.017416858 | 0.505089 |
| R-HSA-166658 | Complement cascade | 6 | 156 | 7 | 120 | 0.010158 | 0.019245552 | 0.538875 |
| R-HSA-9748784 | Drug ADME | 6 | 239 | 0 | 30 | 0.015563 | 0.021937107 | 0.592302 |
| R-HSA-9630794 | Evasion of Oncogene Induced Senescence Due to Defective p16INK4A binding to CDK4 and CDK6 | 1 | 3 | 0 | 0 | 1.95E-04 | 0.02388919 | 0.621119 |
| R-HSA-9632700 | Evasion of Oxidative Stress Induced Senescence Due to Defective p16INK4A binding to CDK4 and CDK6 | 1 | 3 | 0 | 0 | 1.95E-04 | 0.02388919 | 0.621119 |
| R-HSA-69190 | DNA strand elongation | 3 | 38 | 1 | 47 | 0.002474 | 0.024952191 | 0.623805 |
| R-HSA-68867 | Assembly of the pre-replicative complex | 5 | 113 | 4 | 118 | 0.007358 | 0.029471787 | 0.705065 |
| R-HSA-9035968 | Defective GGT1 in aflatoxin detoxification causes GLUTH | 1 | 4 | 0 | 0 | 2.60E-04 | 0.031725439 | 0.705065 |
| R-HSA-9823730 | Formation of definitive endoderm | 3 | 25 | 3 | 62 | 0.001628 | 0.032053967 | 0.705065 |
| R-HSA-69306 | DNA Replication | 6 | 168 | 4 | 159 | 0.01094 | 0.034264908 | 0.705065 |
| R-HSA-5579006 | Defective GSS causes GSS deficiency | 1 | 5 | 0 | 0 | 3.26E-04 | 0.039499121 | 0.705065 |
| R-HSA-8874177 | ATF6B (ATF6-beta) activates chaperones | 1 | 4 | 0 | 1 | 2.60E-04 | 0.039499121 | 0.705065 |
| R-HSA-9013424 | RHOV GTPase cycle | 2 | 39 | 0 | 0 | 0.00254 | 0.040033282 | 0.705065 |
| R-HSA-71406 | Pyruvate metabolism and Citric Acid (TCA) cycle | 4 | 100 | 1 | 65 | 0.006512 | 0.041456616 | 0.705065 |
| R-HSA-9013404 | RAC2 GTPase cycle | 3 | 92 | 2 | 7 | 0.005991 | 0.045596343 | 0.705065 |
| R-HSA-9630747 | Diseases of Cellular Senescence | 2 | 4 | 2 | 40 | 2.60E-04 | 0.047669703 | 0.705065 |
| R-HSA-9675132 | Diseases of cellular response to stress | 2 | 4 | 2 | 40 | 2.60E-04 | 0.047669703 | 0.705065 |
| R-HSA-174577 | Activation of C3 and C5 | 2 | 7 | 2 | 36 | 4.56E-04 | 0.047669703 | 0.705065 |

**Supplementary table 6.** Enriched REACTOME pathways for down-accumulated proteins of the group treated with Agarikon Plus and Agarikon.1

| Pathway identifier | Pathway name | #Entities found | #Entities total | #Interactors found | #Interactors total | Entities ratio | Entities pValue | Entities FDR |
| --- | --- | --- | --- | --- | --- | --- | --- | --- |
| R-HSA-397014 | Muscle contraction | 11 | 232 | 2 | 188 | 0.015107 | 6.76E-09 | 2.25E-06 |
| R-HSA-416572 | Sema4D induced cell migration and growth-cone collapse | 5 | 25 | 0 | 0 | 0.001628 | 7.06E-09 | 2.25E-06 |
| R-HSA-400685 | Sema4D in semaphorin signaling | 5 | 31 | 0 | 0 | 0.002019 | 2.05E-08 | 4.34E-06 |
| R-HSA-445355 | Smooth Muscle Contraction | 7 | 61 | 1 | 83 | 0.003972 | 8.51E-08 | 1.35E-05 |
| R-HSA-3928663 | EPHA-mediated growth cone collapse | 5 | 33 | 0 | 11 | 0.002149 | 1.15E-07 | 1.37E-05 |
| R-HSA-444473 | Formyl peptide receptors bind formyl peptides and many other ligands | 4 | 11 | 0 | 7 | 7.16E-04 | 1.29E-07 | 1.37E-05 |
| R-HSA-390522 | Striated Muscle Contraction | 5 | 40 | 0 | 11 | 0.002605 | 2.37E-07 | 2.16E-05 |
| R-HSA-5625900 | RHO GTPases activate CIT | 5 | 23 | 0 | 64 | 0.001498 | 3.02E-06 | 2.39E-04 |
| R-HSA-5627117 | RHO GTPases Activate ROCKs | 5 | 24 | 1 | 70 | 0.001563 | 4.41E-06 | 3.09E-04 |
| R-HSA-5627123 | RHO GTPases activate PAKs | 5 | 27 | 0 | 130 | 0.001758 | 4.58E-05 | 0.002888 |
| R-HSA-373755 | Semaphorin interactions | 5 | 71 | 2 | 123 | 0.004623 | 1.04E-04 | 0.005914 |
| R-HSA-2682334 | EPH-Ephrin signaling | 8 | 102 | 3 | 623 | 0.006642 | 3.99E-04 | 0.021125 |
| R-HSA-9662361 | Sensory processing of sound by outer hair cells of the cochlea | 3 | 64 | 0 | 11 | 0.004167 | 9.71E-04 | 0.045381 |
| R-HSA-196025 | Formation of annular gap junctions | 2 | 11 | 0 | 7 | 7.16E-04 | 0.001008476 | 0.045381 |
| R-HSA-5625740 | RHO GTPases activate PKNs | 5 | 79 | 0 | 252 | 0.005144 | 0.001426804 | 0.059926 |
| R-HSA-190873 | Gap junction degradation | 2 | 12 | 0 | 14 | 7.81E-04 | 0.001775311 | 0.069237 |
| R-HSA-611105 | Respiratory electron transport | 5 | 118 | 1 | 276 | 0.007684 | 0.002023307 | 0.074862 |
| R-HSA-163200 | Respiratory electron transport, ATP synthesis by chemiosmotic coupling, and heat production by uncoupling proteins. | 5 | 153 | 1 | 276 | 0.009963 | 0.003045907 | 0.106607 |
| R-HSA-445095 | Interaction between L1 and Ankyrins | 2 | 33 | 0 | 0 | 0.002149 | 0.00330739 | 0.109144 |
| R-HSA-9662360 | Sensory processing of sound by inner hair cells of the cochlea | 3 | 76 | 0 | 49 | 0.004949 | 0.004106943 | 0.127315 |
| R-HSA-9659379 | Sensory processing of sound | 3 | 87 | 0 | 49 | 0.005665 | 0.005185961 | 0.155579 |
| R-HSA-6802948 | Signaling by high-kinase activity BRAF mutants | 2 | 44 | 0 | 0 | 0.002865 | 0.005775123 | 0.161703 |
| R-HSA-5626467 | RHO GTPases activate IQGAPs | 2 | 36 | 1 | 11 | 0.002344 | 0.006291507 | 0.163579 |
| R-HSA-9035034 | RHOF GTPase cycle | 2 | 46 | 0 | 0 | 0.002995 | 0.006291507 | 0.163579 |
| R-HSA-5674135 | MAP2K and MAPK activation | 2 | 49 | 0 | 0 | 0.003191 | 0.007104064 | 0.170498 |
| R-HSA-9656223 | Signaling by RAF1 mutants | 2 | 49 | 0 | 0 | 0.003191 | 0.007104064 | 0.170498 |
| R-HSA-194315 | Signaling by Rho GTPases | 9 | 708 | 2 | 742 | 0.046103 | 0.007687127 | 0.176804 |
| R-HSA-9716542 | Signaling by Rho GTPases, Miro GTPases and RHOBTB3 | 9 | 724 | 2 | 754 | 0.047145 | 0.008646539 | 0.190224 |
| R-HSA-195258 | RHO GTPase Effectors | 7 | 325 | 2 | 655 | 0.021163 | 0.0095315 | 0.200161 |
| R-HSA-3656535 | TGFBR1 LBD Mutants in Cancer | 1 | 4 | 0 | 0 | 2.60E-04 | 0.010165987 | 0.204985 |
| R-HSA-1428517 | The citric acid (TCA) cycle and respiratory electron transport | 5 | 238 | 1 | 333 | 0.015498 | 0.010724083 | 0.204985 |
| R-HSA-109606 | Intrinsic Pathway for Apoptosis | 4 | 64 | 3 | 305 | 0.004167 | 0.010788711 | 0.204985 |
| R-HSA-111453 | BH3-only proteins associate with and inactivate anti-apoptotic BCL-2 members | 2 | 11 | 1 | 62 | 7.16E-04 | 0.012179542 | 0.231411 |
| R-HSA-6785807 | Interleukin-4 and Interleukin-13 signaling | 4 | 211 | 1 | 165 | 0.01374 | 0.013974176 | 0.236402 |
| R-HSA-6802955 | Paradoxical activation of RAF signaling by kinase inactive BRAF | 2 | 54 | 0 | 18 | 0.003516 | 0.014775125 | 0.236402 |
| R-HSA-9649948 | Signaling downstream of RAS mutants | 2 | 54 | 0 | 18 | 0.003516 | 0.014775125 | 0.236402 |
| R-HSA-6802946 | Signaling by moderate kinase activity BRAF mutants | 2 | 54 | 0 | 18 | 0.003516 | 0.014775125 | 0.236402 |
| R-HSA-6802949 | Signaling by RAS mutants | 2 | 54 | 0 | 18 | 0.003516 | 0.014775125 | 0.236402 |
| R-HSA-6802952 | Signaling by BRAF and RAF1 fusions | 2 | 73 | 0 | 0 | 0.004754 | 0.015163762 | 0.24262 |
| R-HSA-933543 | NF-kB activation through FADD/RIP-1 pathway mediated by caspase-8 and -10 | 2 | 14 | 4 | 67 | 9.12E-04 | 0.016355899 | 0.245338 |
| R-HSA-1445148 | Translocation of SLC2A4 (GLUT4) to the plasma membrane | 3 | 81 | 1 | 139 | 0.005274 | 0.017575454 | 0.248133 |
| R-HSA-844455 | The NLRP1 inflammasome | 1 | 5 | 1 | 4 | 3.26E-04 | 0.017723759 | 0.248133 |
| R-HSA-3656534 | Loss of Function of TGFBR1 in Cancer | 1 | 7 | 0 | 0 | 4.56E-04 | 0.017723759 | 0.248133 |
| R-HSA-111464 | SMAC(DIABLO)-mediated dissociation of IAP:caspase complexes | 1 | 7 | 0 | 0 | 4.56E-04 | 0.017723759 | 0.248133 |
| R-HSA-75158 | TRAIL signaling | 2 | 8 | 3 | 80 | 5.21E-04 | 0.018428776 | 0.258003 |
| R-HSA-9664417 | Leishmania phagocytosis | 3 | 157 | 0 | 78 | 0.010223 | 0.020031448 | 0.260409 |
| R-HSA-9664422 | FCGR3A-mediated phagocytosis | 3 | 157 | 0 | 78 | 0.010223 | 0.020031448 | 0.260409 |
| R-HSA-9664407 | Parasite infection | 3 | 157 | 0 | 78 | 0.010223 | 0.020031448 | 0.260409 |
| R-HSA-9636249 | Inhibition of nitric oxide production | 0 | 5 | 1 | 3 | 3.26E-04 | 0.020230391 | 0.262995 |
| R-HSA-5663213 | RHO GTPases Activate WASPs and WAVEs | 2 | 41 | 0 | 57 | 0.00267 | 0.022422404 | 0.269069 |
| R-HSA-75157 | FasL/ CD95L signaling | 2 | 5 | 3 | 91 | 3.26E-04 | 0.022886392 | 0.274637 |
| R-HSA-3928665 | EPH-ephrin mediated repulsion of cells | 2 | 55 | 0 | 39 | 0.003581 | 0.023826246 | 0.285915 |
| R-HSA-111448 | Activation of NOXA and translocation to mitochondria | 0 | 6 | 2 | 5 | 3.91E-04 | 0.02771263 | 0.332552 |
| R-HSA-5218900 | CASP8 activity is inhibited | 1 | 12 | 2 | 3 | 7.81E-04 | 0.032669563 | 0.359365 |
| R-HSA-6802957 | Oncogenic MAPK signaling | 2 | 93 | 0 | 18 | 0.006056 | 0.032969748 | 0.362667 |
| R-HSA-6798695 | Neutrophil degranulation | 4 | 478 | 0 | 0 | 0.031126 | 0.033923058 | 0.373154 |
| R-HSA-139915 | Activation of PUMA and translocation to mitochondria | 0 | 10 | 1 | 5 | 6.51E-04 | 0.037601658 | 0.376017 |
| R-HSA-9636667 | Manipulation of host energy metabolism | 0 | 3 | 1 | 12 | 1.95E-04 | 0.037601658 | 0.376017 |
| R-HSA-437239 | Recycling pathway of L1 | 2 | 55 | 1 | 77 | 0.003581 | 0.04146927 | 0.414693 |
| R-HSA-140342 | Apoptosis induced DNA fragmentation | 1 | 13 | 0 | 6 | 8.47E-04 | 0.042509037 | 0.42509 |
| R-HSA-375276 | Peptide ligand-binding receptors | 4 | 203 | 0 | 344 | 0.013219 | 0.04268428 | 0.426843 |
| R-HSA-9818030 | NFE2L2 regulating tumorigenic genes | 2 | 17 | 1 | 119 | 0.001107 | 0.045076243 | 0.450762 |
| R-HSA-2029482 | Regulation of actin dynamics for phagocytic cup formation | 3 | 158 | 1 | 173 | 0.010288 | 0.047237075 | 0.460056 |

**Supplementary table 7.** Enriched REACTOME pathways for up-accumulated proteins of the 5-fluorouracil treated group

| Pathway identifier | Pathway name | #Entities found | #Entities total | #Interactors found | #Interactors total | Entities ratio | Entities pValue | Entities FDR |
| --- | --- | --- | --- | --- | --- | --- | --- | --- |
| R-HSA-192823 | Viral mRNA Translation | 6 | 114 | 0 | 23 | 0.007423 | 4.01E-06 | 0.00222 |
| R-HSA-1989781 | PPARA activates gene expression | 10 | 175 | 2 | 408 | 0.011395 | 8.86E-06 | 0.00222 |
| R-HSA-400206 | Regulation of lipid metabolism by PPARalpha | 10 | 177 | 3 | 443 | 0.011526 | 1.36E-05 | 0.00222 |
| R-HSA-156902 | Peptide chain elongation | 5 | 97 | 0 | 7 | 0.006316 | 1.77E-05 | 0.00222 |
| R-HSA-8957275 | Post-translational protein phosphorylation | 5 | 109 | 0 | 0 | 0.007098 | 2.22E-05 | 0.00222 |
| R-HSA-72764 | Eukaryotic Translation Termination | 5 | 106 | 0 | 4 | 0.006902 | 2.22E-05 | 0.00222 |
| R-HSA-72689 | Formation of a pool of free 40S subunits | 5 | 106 | 0 | 7 | 0.006902 | 2.42E-05 | 0.00222 |
| R-HSA-2408557 | Selenocysteine synthesis | 5 | 112 | 0 | 3 | 0.007293 | 2.86E-05 | 0.00222 |
| R-HSA-168273 | Influenza Viral RNA Transcription and Replication | 7 | 173 | 1 | 127 | 0.011265 | 2.99E-05 | 0.00222 |
| R-HSA-1799339 | SRP-dependent cotranslational protein targeting to membrane | 5 | 119 | 0 | 0 | 0.007749 | 3.36E-05 | 0.00222 |
| R-HSA-975956 | Nonsense Mediated Decay (NMD) independent of the Exon Junction Complex (EJC) | 5 | 101 | 0 | 24 | 0.006577 | 3.64E-05 | 0.00222 |
| R-HSA-168255 | Influenza Infection | 9 | 198 | 4 | 362 | 0.012893 | 3.64E-05 | 0.00222 |
| R-HSA-156827 | L13a-mediated translational silencing of Ceruloplasmin expression | 5 | 120 | 0 | 5 | 0.007814 | 4.24E-05 | 0.002374 |
| R-HSA-5660526 | Response to metal ions | 4 | 21 | 0 | 52 | 0.001367 | 7.03E-05 | 0.003653 |
| R-HSA-156842 | Eukaryotic Translation Elongation | 5 | 102 | 0 | 60 | 0.006642 | 1.27E-04 | 0.006203 |
| R-HSA-72706 | GTP hydrolysis and joining of the 60S ribosomal subunit | 5 | 120 | 0 | 61 | 0.007814 | 1.73E-04 | 0.007946 |
| R-HSA-2408522 | Selenoamino acid metabolism | 5 | 180 | 0 | 11 | 0.011721 | 3.03E-04 | 0.013011 |
| R-HSA-927802 | Nonsense-Mediated Decay (NMD) | 5 | 124 | 0 | 100 | 0.008074 | 4.45E-04 | 0.016926 |
| R-HSA-975957 | Nonsense Mediated Decay (NMD) enhanced by the Exon Junction Complex (EJC) | 5 | 124 | 0 | 100 | 0.008074 | 4.45E-04 | 0.016926 |
| R-HSA-72737 | Cap-dependent Translation Initiation | 5 | 130 | 0 | 106 | 0.008465 | 5.97E-04 | 0.021495 |
| R-HSA-72613 | Eukaryotic Translation Initiation | 5 | 130 | 0 | 109 | 0.008465 | 6.34E-04 | 0.022193 |
| R-HSA-5661231 | Metallothioneins bind metals | 3 | 16 | 0 | 43 | 0.001042 | 7.32E-04 | 0.024163 |
| R-HSA-381426 | Regulation of Insulin-like Growth Factor (IGF) transport and uptake by Insulin-like Growth Factor Binding Proteins (IGFBPs) | 5 | 127 | 0 | 134 | 0.00827 | 0.001144291 | 0.036617 |
| R-HSA-9010553 | Regulation of expression of SLITs and ROBOs | 5 | 183 | 0 | 100 | 0.011916 | 0.001515113 | 0.045453 |
| R-HSA-9633012 | Response of EIF2AK4 (GCN2) to amino acid deficiency | 5 | 115 | 0 | 195 | 0.007488 | 0.002375609 | 0.068893 |
| R-HSA-381183 | ATF6 (ATF6-alpha) activates chaperone genes | 4 | 15 | 4 | 210 | 9.77E-04 | 0.004618931 | 0.128726 |
| R-HSA-381033 | ATF6 (ATF6-alpha) activates chaperones | 4 | 17 | 4 | 210 | 0.001107 | 0.004767629 | 0.128726 |
| R-HSA-9711097 | Cellular response to starvation | 5 | 176 | 0 | 204 | 0.011461 | 0.005691894 | 0.147989 |
| R-HSA-8953897 | Cellular responses to stimuli | 20 | 1051 | 13 | 3011 | 0.068438 | 0.007865371 | 0.196634 |
| R-HSA-3371568 | Attenuation phase | 2 | 47 | 0 | 0 | 0.00306 | 0.009167589 | 0.220022 |
| R-HSA-3000480 | Scavenging by Class A Receptors | 2 | 49 | 0 | 0 | 0.003191 | 0.00992569 | 0.228291 |
| R-HSA-3371571 | HSF1-dependent transactivation | 2 | 59 | 0 | 0 | 0.003842 | 0.014113329 | 0.324607 |
| R-HSA-8874177 | ATF6B (ATF6-beta) activates chaperones | 1 | 4 | 0 | 1 | 2.60E-04 | 0.015080361 | 0.325386 |
| R-HSA-8963888 | Chylomicron assembly | 2 | 14 | 1 | 51 | 9.12E-04 | 0.015494571 | 0.325386 |
| R-HSA-5682113 | Defective ABCA1 causes TGD | 1 | 5 | 0 | 3 | 3.26E-04 | 0.024020764 | 0.479709 |
| R-HSA-9636249 | Inhibition of nitric oxide production | 1 | 5 | 0 | 3 | 3.26E-04 | 0.024020764 | 0.479709 |
| R-HSA-8963901 | Chylomicron remodeling | 2 | 17 | 1 | 66 | 0.001107 | 0.026073883 | 0.479709 |
| R-HSA-381340 | Transcriptional regulation of white adipocyte differentiation | 5 | 109 | 1 | 471 | 0.007098 | 0.026807805 | 0.479709 |
| R-HSA-6791226 | Major pathway of rRNA processing in the nucleolus and cytosol | 5 | 189 | 2 | 410 | 0.012307 | 0.029411272 | 0.479709 |
| R-HSA-168276 | NS1 Mediated Effects on Host Pathways | 2 | 46 | 2 | 45 | 0.002995 | 0.030928375 | 0.479709 |
| R-HSA-8868773 | rRNA processing in the nucleus and cytosol | 5 | 207 | 2 | 412 | 0.013479 | 0.032987837 | 0.479709 |
| R-HSA-376176 | Signaling by ROBO receptors | 5 | 235 | 0 | 413 | 0.015302 | 0.04164237 | 0.479709 |
| R-HSA-3371453 | Regulation of HSF1-mediated heat shock response | 3 | 113 | 0 | 155 | 0.007358 | 0.045014976 | 0.479709 |
| R-HSA-5660489 | MTF1 activates gene expression | 1 | 6 | 0 | 10 | 3.91E-04 | 0.047472695 | 0.479709 |
| R-HSA-8963898 | Plasma lipoprotein assembly | 2 | 30 | 1 | 89 | 0.001954 | 0.048127266 | 0.479709 |

**Supplementary table 8.** Enriched REACTOME pathways for down-accumulated proteins of the 5-fluorouracil treated group

| Pathway identifier | Pathway name | #Entities found | #Entities total | #Interactors found | #Interactors total | Entities ratio | Entities pValue | Entities FDR |
| --- | --- | --- | --- | --- | --- | --- | --- | --- |
| R-HSA-5603041 | IRAK4 deficiency (TLR2/4) | 3 | 27 | 0 | 11 | 0.001758 | 3.19E-06 | 0.00116 |
| R-HSA-5602498 | MyD88 deficiency (TLR2/4) | 3 | 26 | 1 | 24 | 0.001693 | 6.63E-06 | 0.0012 |
| R-HSA-5686938 | Regulation of TLR by endogenous ligand | 3 | 36 | 1 | 25 | 0.002344 | 1.46E-05 | 0.001768 |
| R-HSA-109606 | Intrinsic Pathway for Apoptosis | 4 | 64 | 3 | 305 | 0.004167 | 1.24E-04 | 0.00933 |
| R-HSA-6799990 | Metal sequestration by antimicrobial proteins | 2 | 13 | 0 | 9 | 8.47E-04 | 1.30E-04 | 0.00933 |
| R-HSA-5260271 | Diseases of Immune System | 3 | 42 | 1 | 163 | 0.002735 | 4.49E-04 | 0.020199 |
| R-HSA-5602358 | Diseases associated with the TLR signaling cascade | 3 | 42 | 1 | 163 | 0.002735 | 4.49E-04 | 0.020199 |
| R-HSA-140534 | Caspase activation via Death Receptors in the presence of ligand | 2 | 20 | 2 | 32 | 0.001302 | 4.89E-04 | 0.020199 |
| R-HSA-5357769 | Caspase activation via extrinsic apoptotic signalling pathway | 3 | 32 | 2 | 182 | 0.002084 | 5.05E-04 | 0.020199 |
| R-HSA-166058 | MyD88:MAL(TIRAP) cascade initiated on plasma membrane | 4 | 133 | 1 | 494 | 0.008661 | 9.59E-04 | 0.023606 |
| R-HSA-168179 | Toll Like Receptor TLR1:TLR2 Cascade | 4 | 136 | 1 | 495 | 0.008856 | 9.83E-04 | 0.023606 |
| R-HSA-168188 | Toll Like Receptor TLR6:TLR2 Cascade | 4 | 133 | 1 | 499 | 0.008661 | 9.90E-04 | 0.023606 |
| R-HSA-181438 | Toll Like Receptor 2 (TLR2) Cascade | 4 | 136 | 1 | 499 | 0.008856 | 0.001008847 | 0.023606 |
| R-HSA-2562578 | TRIF-mediated programmed cell death | 2 | 10 | 3 | 55 | 6.51E-04 | 0.001072993 | 0.023606 |
| R-HSA-111453 | BH3-only proteins associate with and inactivate anti-apoptotic BCL-2 members | 2 | 11 | 1 | 62 | 7.16E-04 | 0.001216043 | 0.025537 |
| R-HSA-1236974 | ER-Phagosome pathway | 3 | 106 | 0 | 197 | 0.006902 | 0.001516197 | 0.030324 |
| R-HSA-933543 | NF-kB activation through FADD/RIP-1 pathway mediated by caspase-8 and -10 | 2 | 14 | 3 | 67 | 9.12E-04 | 0.001653901 | 0.031424 |
| R-HSA-75158 | TRAIL signaling | 2 | 8 | 3 | 80 | 5.21E-04 | 0.00187428 | 0.031968 |
| R-HSA-1236975 | Antigen processing-Cross presentation | 3 | 128 | 0 | 198 | 0.008335 | 0.001880457 | 0.031968 |
| R-HSA-166016 | Toll Like Receptor 4 (TLR4) Cascade | 4 | 165 | 4 | 636 | 0.010744 | 0.002321418 | 0.035319 |
| R-HSA-75157 | FasL/ CD95L signaling | 2 | 5 | 3 | 91 | 3.26E-04 | 0.002354571 | 0.035319 |
| R-HSA-109581 | Apoptosis | 5 | 193 | 5 | 1149 | 0.012568 | 0.00249186 | 0.037378 |
| R-HSA-168898 | Toll-like Receptor Cascades | 4 | 202 | 4 | 715 | 0.013154 | 0.003874183 | 0.054239 |
| R-HSA-9818030 | NFE2L2 regulating tumorigenic genes | 2 | 17 | 1 | 119 | 0.001107 | 0.004860008 | 0.06318 |
| R-HSA-844455 | The NLRP1 inflammasome | 1 | 5 | 1 | 4 | 3.26E-04 | 0.005534433 | 0.066413 |
| R-HSA-111464 | SMAC(DIABLO)-mediated dissociation of IAP:caspase complexes | 1 | 7 | 0 | 0 | 4.56E-04 | 0.005534433 | 0.066413 |
| R-HSA-9636249 | Inhibition of nitric oxide production | 0 | 5 | 2 | 3 | 3.26E-04 | 0.006322701 | 0.075872 |
| R-HSA-6803157 | Antimicrobial peptides | 2 | 123 | 0 | 51 | 0.008009 | 0.007829687 | 0.093956 |
| R-HSA-5357801 | Programmed Cell Death | 5 | 238 | 6 | 1549 | 0.015498 | 0.008620327 | 0.094824 |
| R-HSA-111448 | Activation of NOXA and translocation to mitochondria | 0 | 6 | 2 | 5 | 3.91E-04 | 0.008683965 | 0.095524 |
| R-HSA-5218900 | CASP8 activity is inhibited | 1 | 12 | 2 | 3 | 7.81E-04 | 0.010255196 | 0.102982 |
| R-HSA-5668599 | RHO GTPases Activate NADPH Oxidases | 2 | 38 | 0 | 157 | 0.002474 | 0.010298212 | 0.102982 |
| R-HSA-139915 | Activation of PUMA and translocation to mitochondria | 0 | 10 | 1 | 5 | 6.51E-04 | 0.011824075 | 0.118241 |
| R-HSA-140342 | Apoptosis induced DNA fragmentation | 1 | 13 | 0 | 6 | 8.47E-04 | 0.013390605 | 0.132147 |
| R-HSA-983169 | Class I MHC mediated antigen processing & presentation | 3 | 412 | 0 | 273 | 0.026828 | 0.014682995 | 0.132147 |
| R-HSA-9634638 | Estrogen-dependent nuclear events downstream of ESR-membrane signaling | 2 | 29 | 1 | 220 | 0.001888 | 0.015395634 | 0.138561 |
| R-HSA-75108 | Activation, myristolyation of BID and translocation to mitochondria | 1 | 4 | 1 | 18 | 2.60E-04 | 0.017296675 | 0.15567 |
| R-HSA-211736 | Stimulation of the cell death response by PAK-2p34 | 1 | 4 | 0 | 25 | 2.60E-04 | 0.02196468 | 0.167942 |
| R-HSA-5660668 | CLEC7A/inflammasome pathway | 1 | 8 | 1 | 25 | 5.21E-04 | 0.023516022 | 0.167942 |
| R-HSA-3371378 | Regulation by c-FLIP | 1 | 11 | 2 | 32 | 7.16E-04 | 0.02506504 | 0.167942 |
| R-HSA-69416 | Dimerization of procaspase-8 | 1 | 11 | 1 | 31 | 7.16E-04 | 0.02506504 | 0.167942 |
| R-HSA-114294 | Activation, translocation and oligomerization of BAX | 0 | 2 | 1 | 32 | 1.30E-04 | 0.02506504 | 0.167942 |
| R-HSA-174490 | Membrane binding and targetting of GAG proteins | 0 | 17 | 1 | 15 | 0.001107 | 0.02506504 | 0.167942 |
| R-HSA-174495 | Synthesis And Processing Of GAG, GAGPOL Polyproteins | 0 | 18 | 1 | 15 | 0.001172 | 0.025838678 | 0.167942 |
| R-HSA-111452 | Activation and oligomerization of BAK protein | 0 | 2 | 1 | 37 | 1.30E-04 | 0.028927438 | 0.167942 |
| R-HSA-205025 | NADE modulates death signalling | 1 | 6 | 0 | 32 | 3.91E-04 | 0.028927438 | 0.167942 |
| R-HSA-168249 | Innate Immune System | 6 | 1341 | 6 | 2092 | 0.087322 | 0.029591513 | 0.167942 |
| R-HSA-111447 | Activation of BAD and translocation to mitochondria | 1 | 19 | 1 | 27 | 0.001237 | 0.029698182 | 0.167942 |
| R-HSA-168927 | TICAM1, RIP1-mediated IKK complex recruitment | 0 | 19 | 1 | 26 | 0.001237 | 0.031237936 | 0.167942 |
| R-HSA-6803211 | TP53 Regulates Transcription of Death Receptors and Ligands | 0 | 18 | 1 | 25 | 0.001172 | 0.031237936 | 0.167942 |
| R-HSA-111446 | Activation of BIM and translocation to mitochondria | 0 | 5 | 1 | 37 | 3.26E-04 | 0.032006947 | 0.167942 |
| R-HSA-1810476 | RIP-mediated NFkB activation via ZBP1 | 0 | 19 | 1 | 26 | 0.001237 | 0.032775382 | 0.167942 |
| R-HSA-6785807 | Interleukin-4 and Interleukin-13 signaling | 2 | 211 | 1 | 165 | 0.01374 | 0.032998565 | 0.167942 |
| R-HSA-937041 | IKK complex recruitment mediated by RIP1 | 1 | 24 | 1 | 26 | 0.001563 | 0.035077231 | 0.167942 |
| R-HSA-264870 | Caspase-mediated cleavage of cytoskeletal proteins | 2 | 12 | 0 | 379 | 7.81E-04 | 0.037090185 | 0.167942 |
| R-HSA-9603505 | NTRK3 as a dependence receptor | 0 | 3 | 1 | 47 | 1.95E-04 | 0.038138316 | 0.167942 |
| R-HSA-8952158 | RUNX3 regulates BCL2L11 (BIM) transcription | 0 | 6 | 1 | 47 | 3.91E-04 | 0.038902152 | 0.167942 |
| R-HSA-198693 | AKT phosphorylates targets in the nucleus | 0 | 12 | 1 | 39 | 7.81E-04 | 0.038902152 | 0.167942 |
| R-HSA-3134963 | DEx/H-box helicases activate type I IFN and inflammatory cytokines production | 0 | 7 | 1 | 45 | 4.56E-04 | 0.038902152 | 0.167942 |
| R-HSA-9013957 | TLR3-mediated TICAM1-dependent programmed cell death | 1 | 6 | 3 | 55 | 3.91E-04 | 0.044232992 | 0.167942 |
| R-HSA-111463 | SMAC (DIABLO) binds to IAPs | 1 | 7 | 1 | 59 | 4.56E-04 | 0.047266635 | 0.167942 |
| R-HSA-111469 | SMAC, XIAP-regulated apoptotic response | 1 | 8 | 1 | 59 | 5.21E-04 | 0.048023624 | 0.167942 |
| R-HSA-9693928 | Defective RIPK1-mediated regulated necrosis | 1 | 13 | 1 | 56 | 8.47E-04 | 0.048023624 | 0.167942 |
| R-HSA-9686347 | Microbial modulation of RIPK1-mediated regulated necrosis | 1 | 10 | 1 | 56 | 6.51E-04 | 0.048023624 | 0.167942 |
| R-HSA-5676934 | Protein repair | 0 | 19 | 1 | 43 | 0.001237 | 0.048023624 | 0.167942 |
| R-HSA-111459 | Activation of caspases through apoptosome-mediated cleavage | 1 | 8 | 1 | 59 | 5.21E-04 | 0.048780045 | 0.167942 |
