# Supplementary material for: Proteomic study of medicinal mushroom extracts reveals antitumor mechanisms in an advanced colon cancer animal model via ribosomal biogenesis, translation, and metabolic pathways
Source: Front Pharmacol. 2024 Oct 18;15:1475102. doi: 10.3389/fphar.2024.1475102 (PMC11528127; doi:10.3389/fphar.2024.1475102)
Supplement: Supplementary file 2 [file DataSheet1.docx]

| 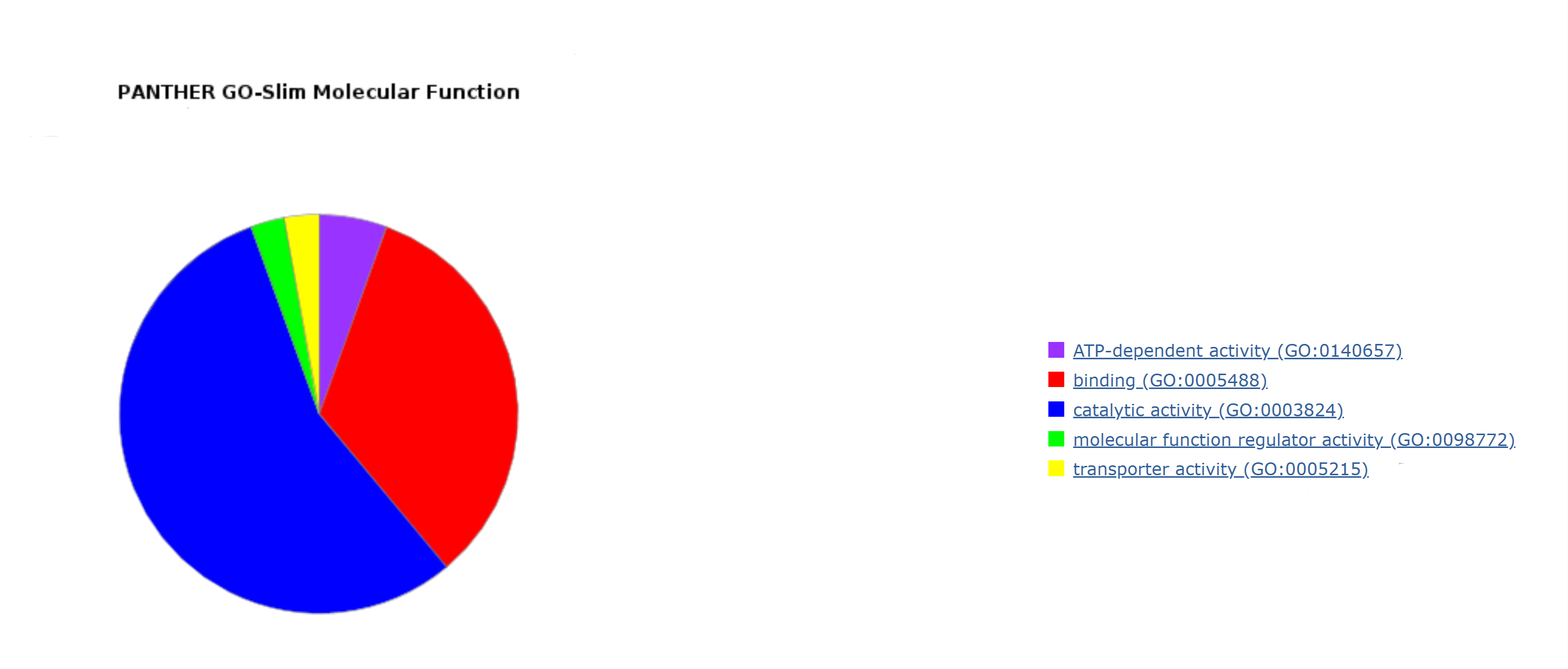 | 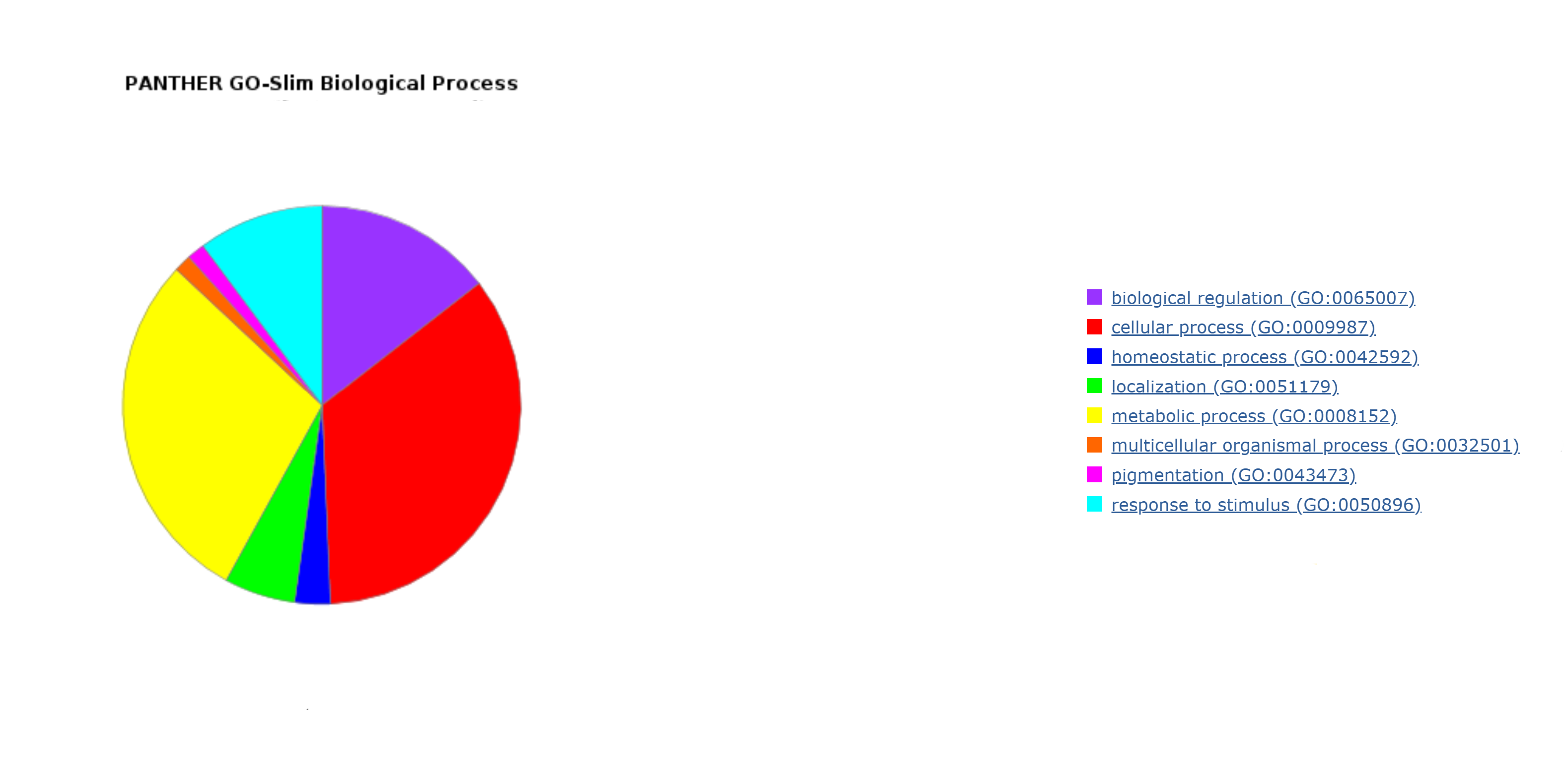 | |
| --- | --- | --- |
| (**a**) | | |
| 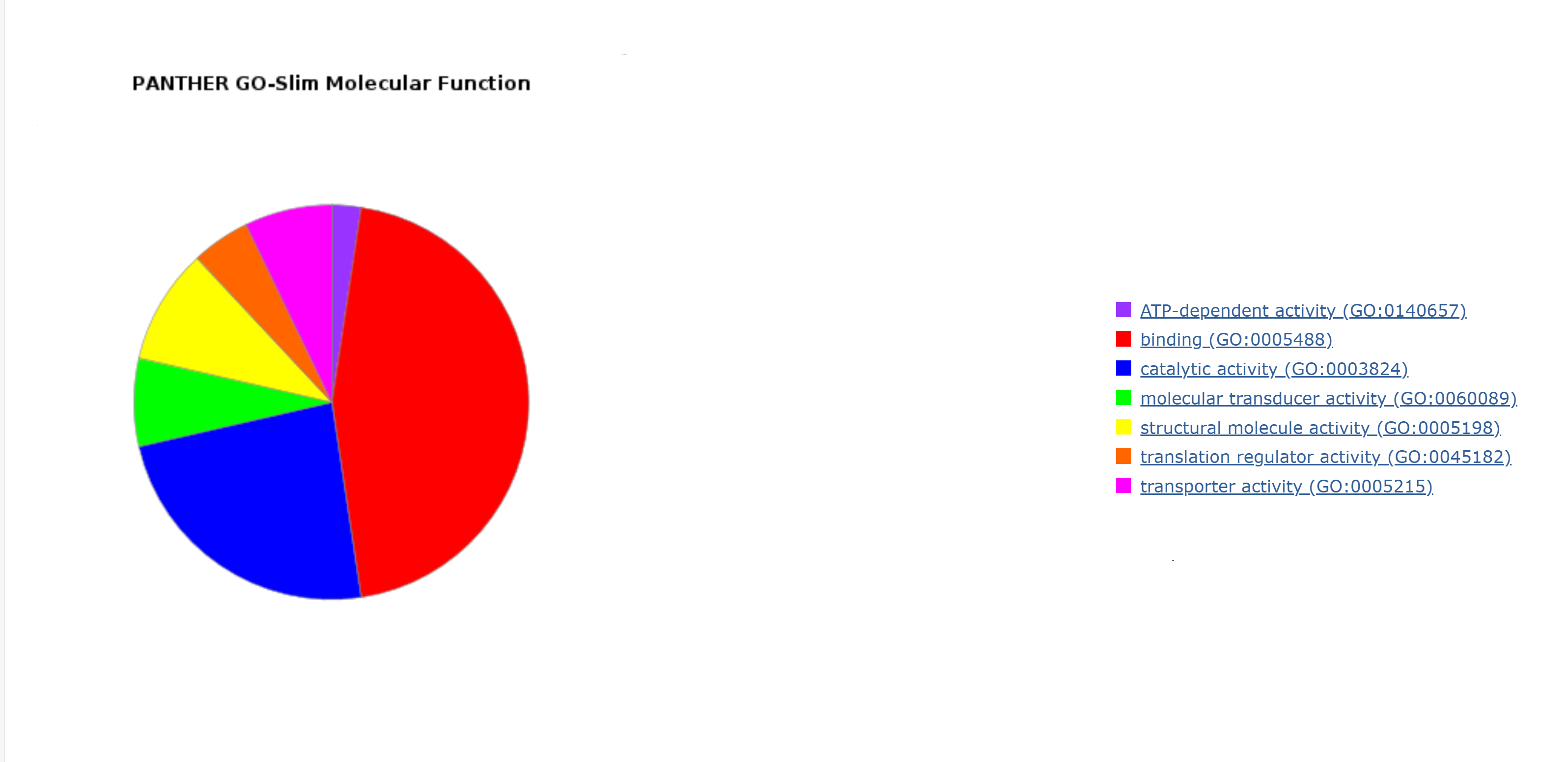 | 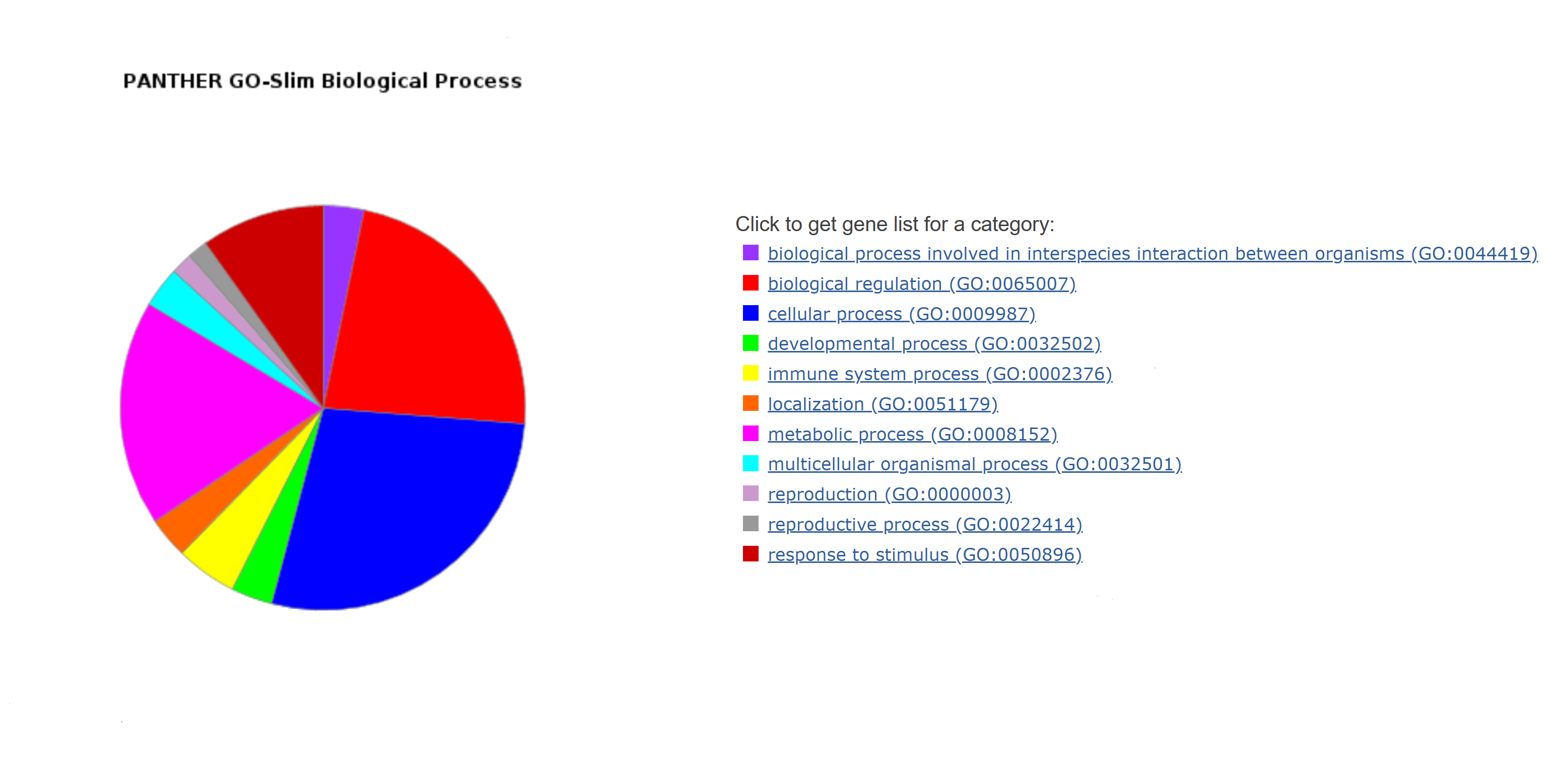 | |
| (**b**) | | |
| 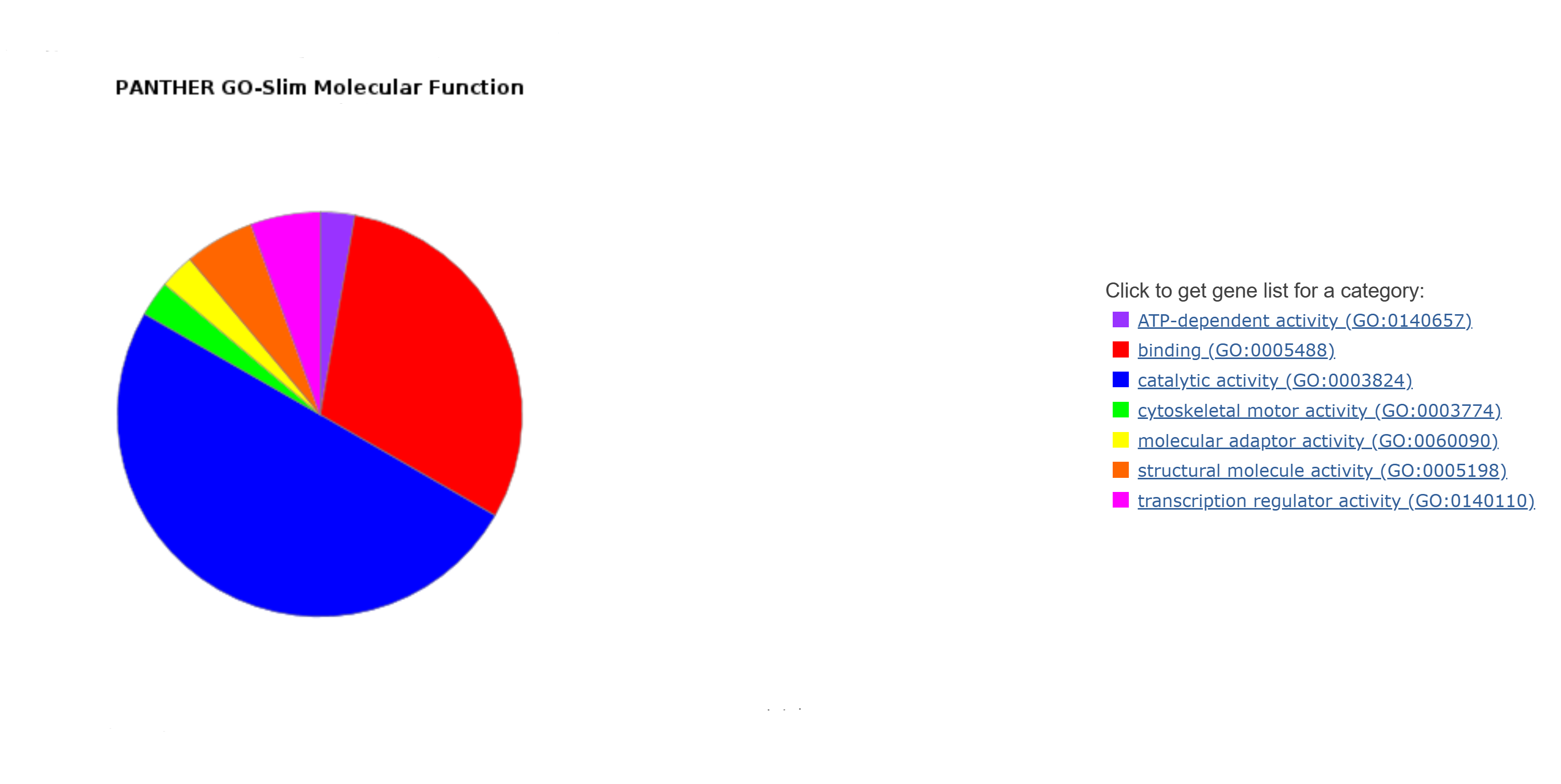 | 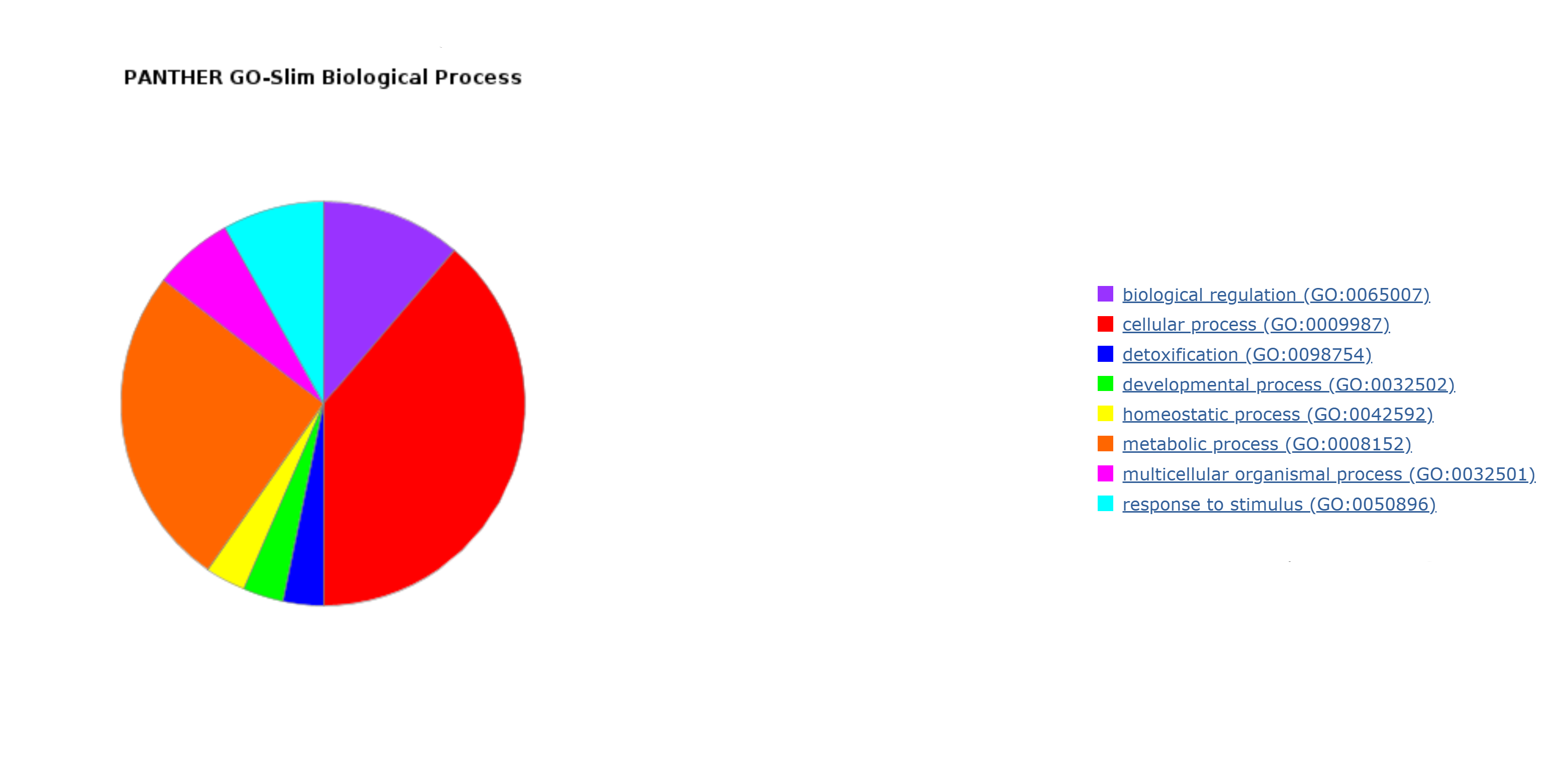 | |
| (**c**) | | |
| 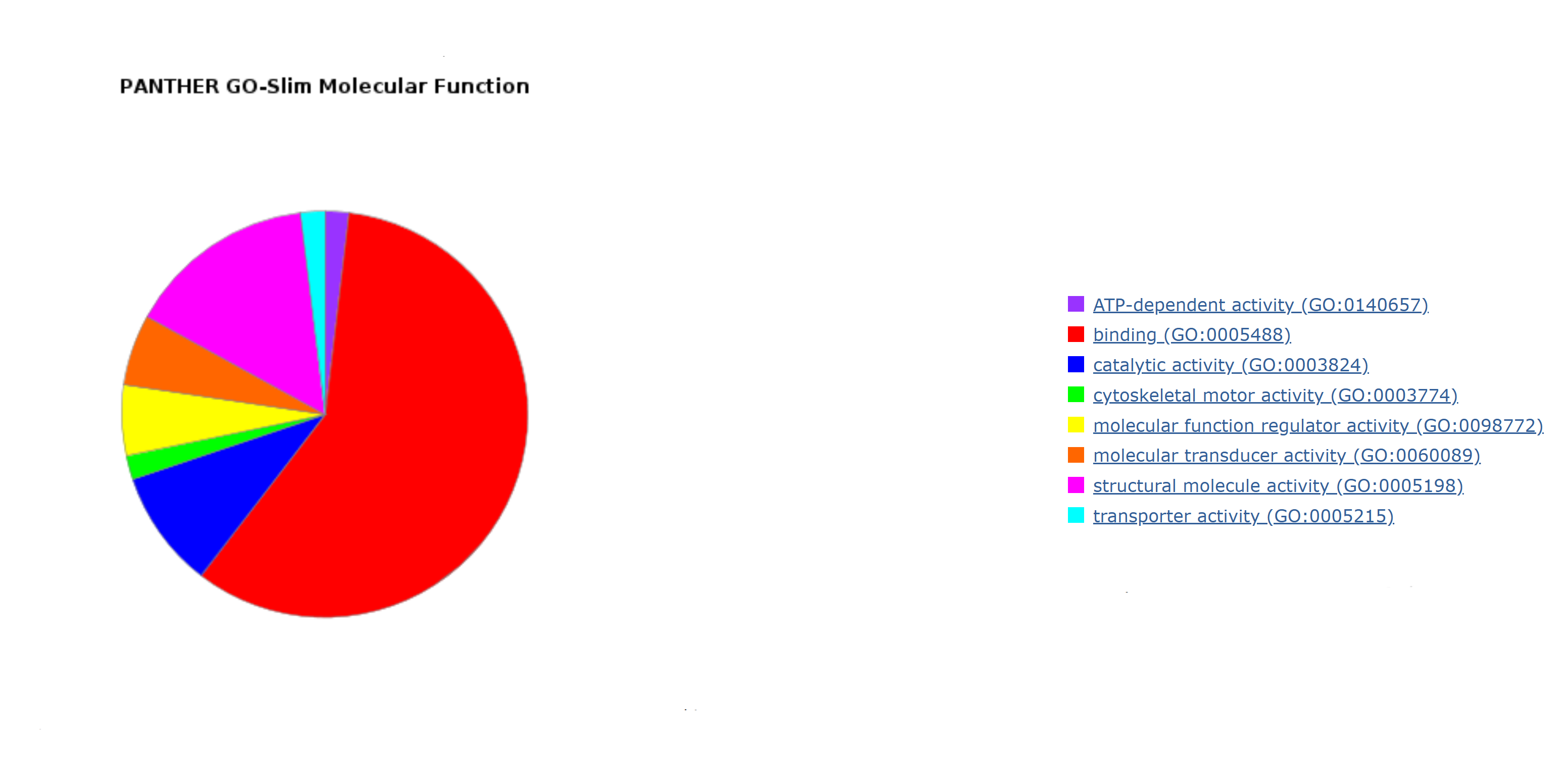 | 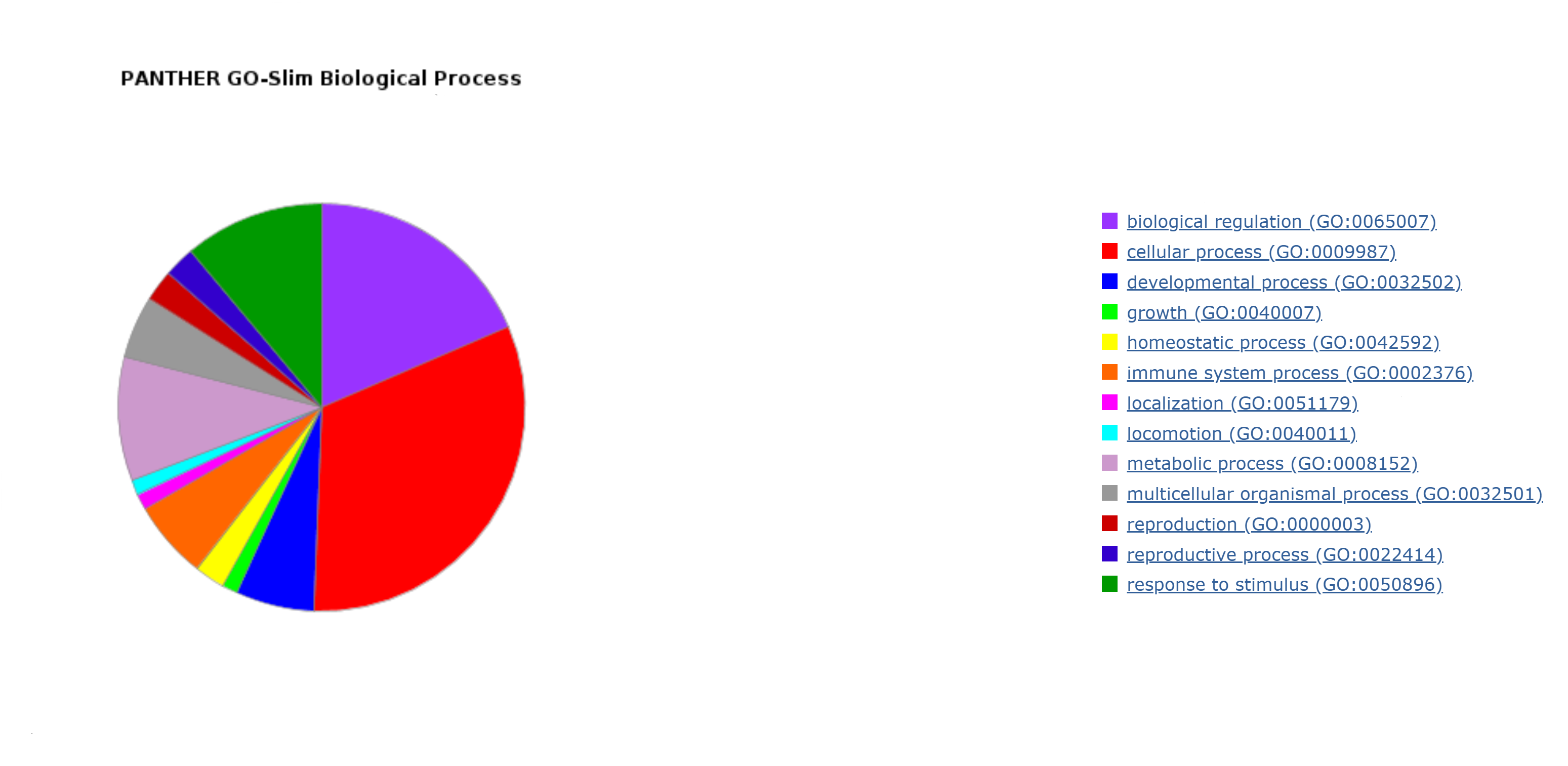 | |
| (**d**) | | |
| 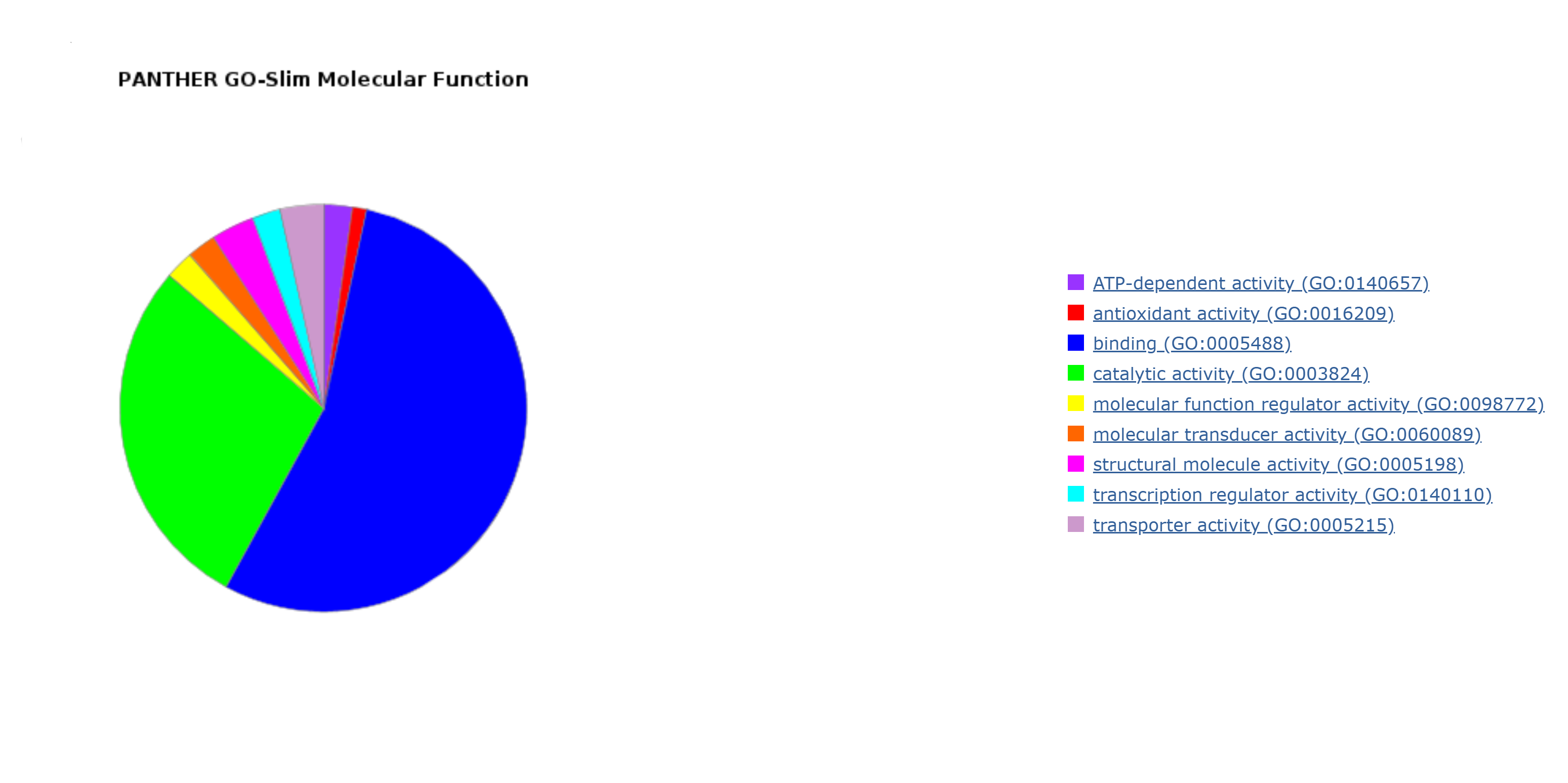 | 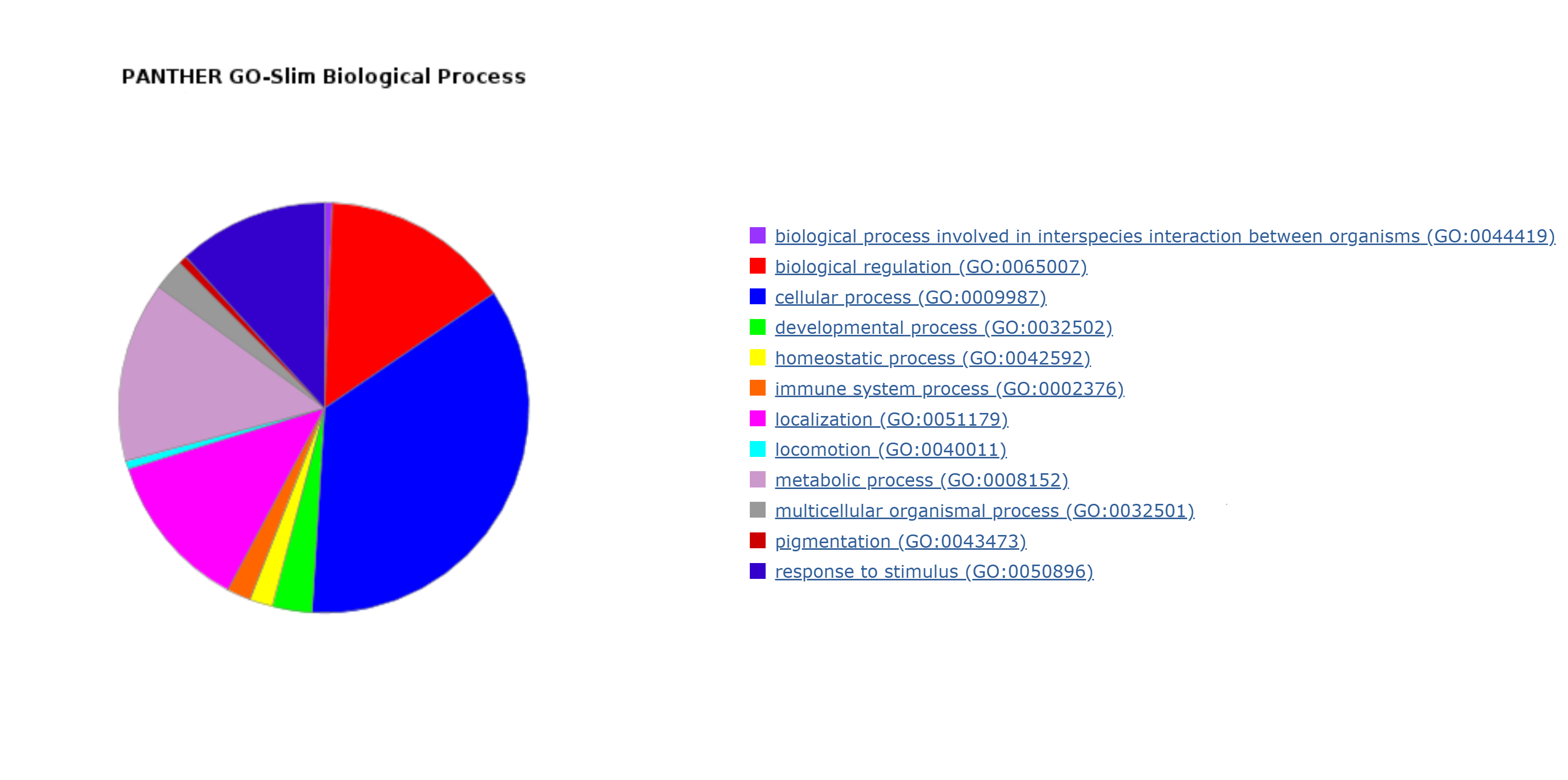 | |
| (**e**) | | |
| 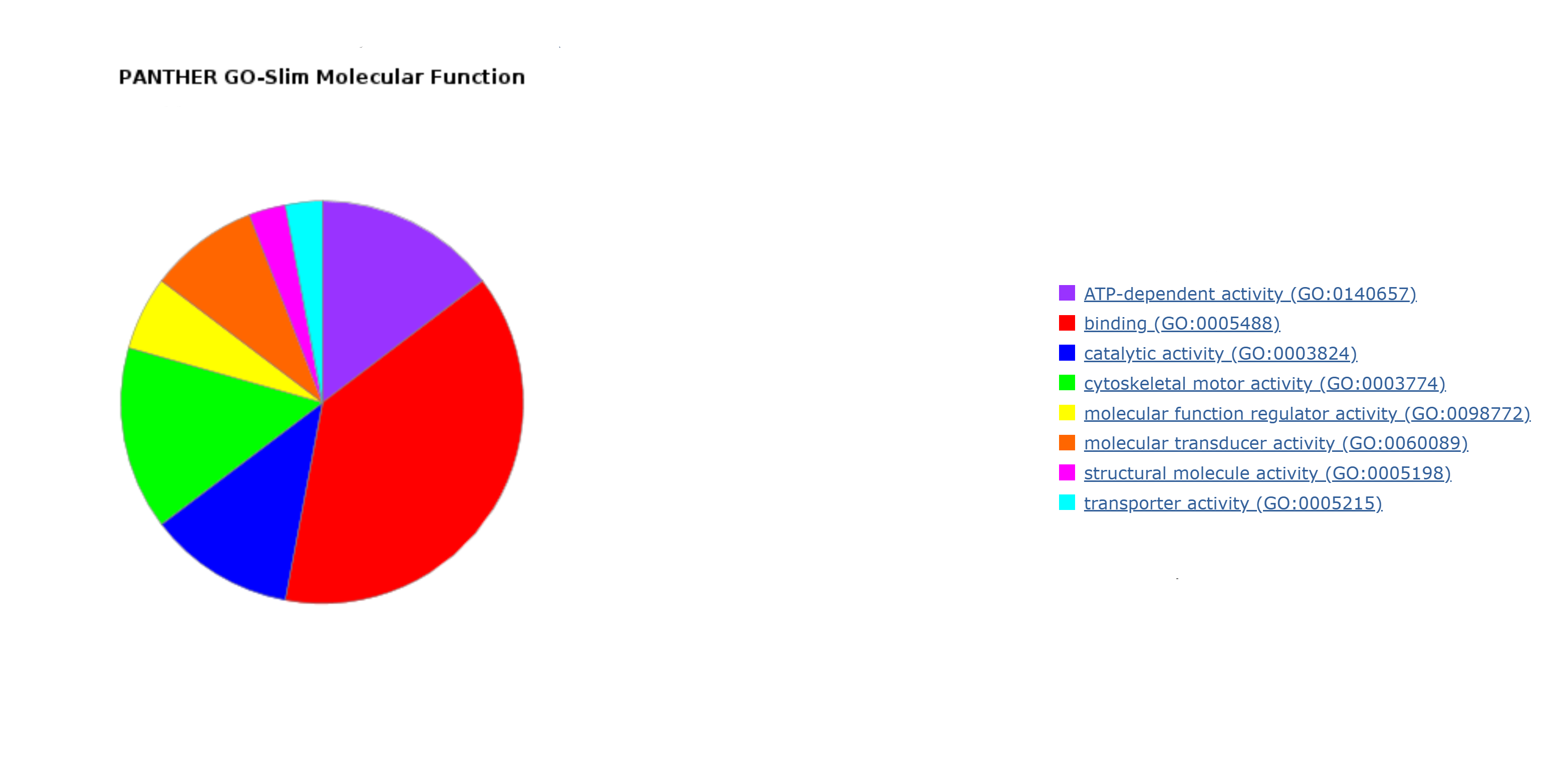 | 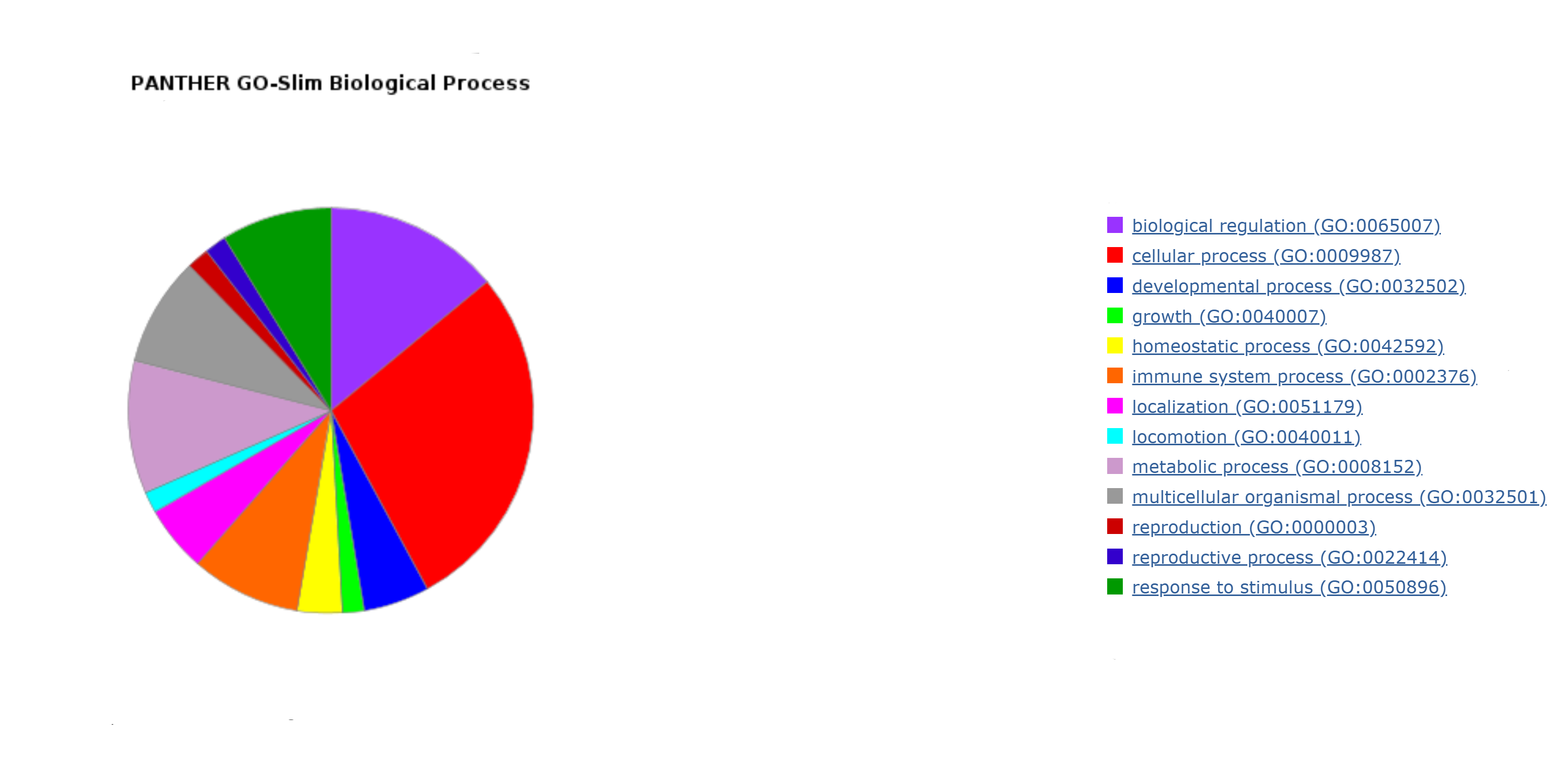 | |
| (**f**) | | |
| 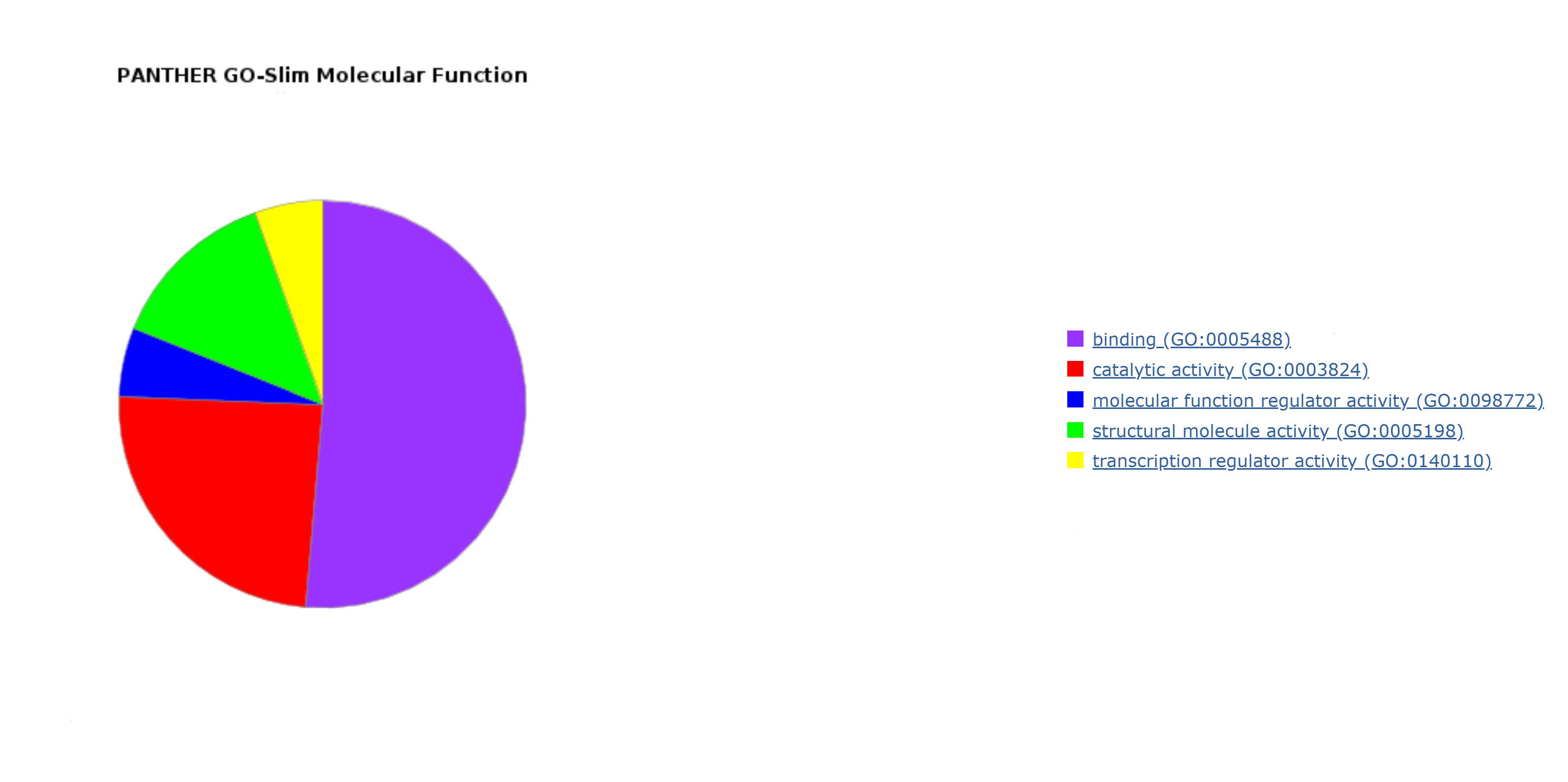 | | 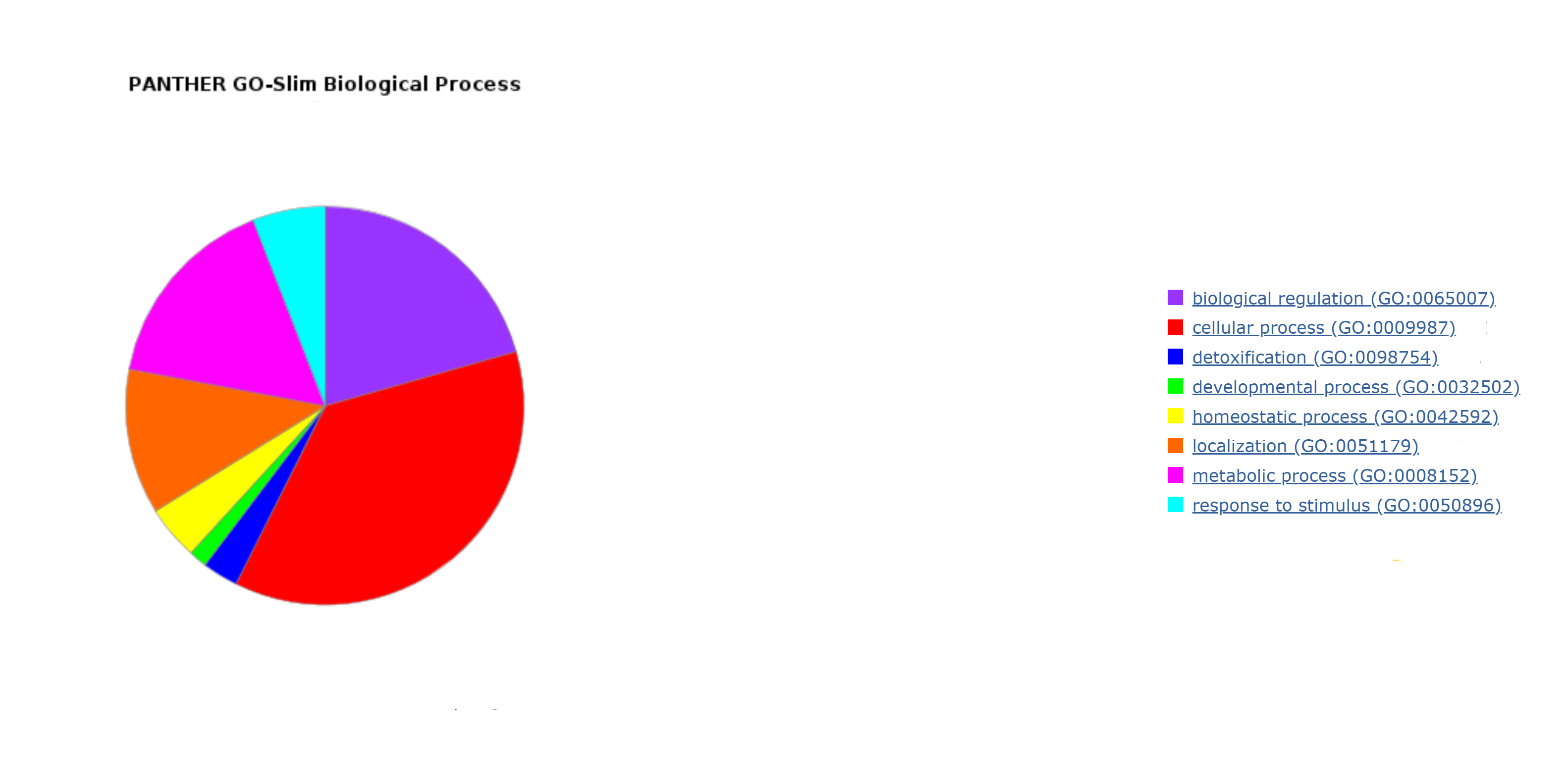 |
| **(g)** | | |
| 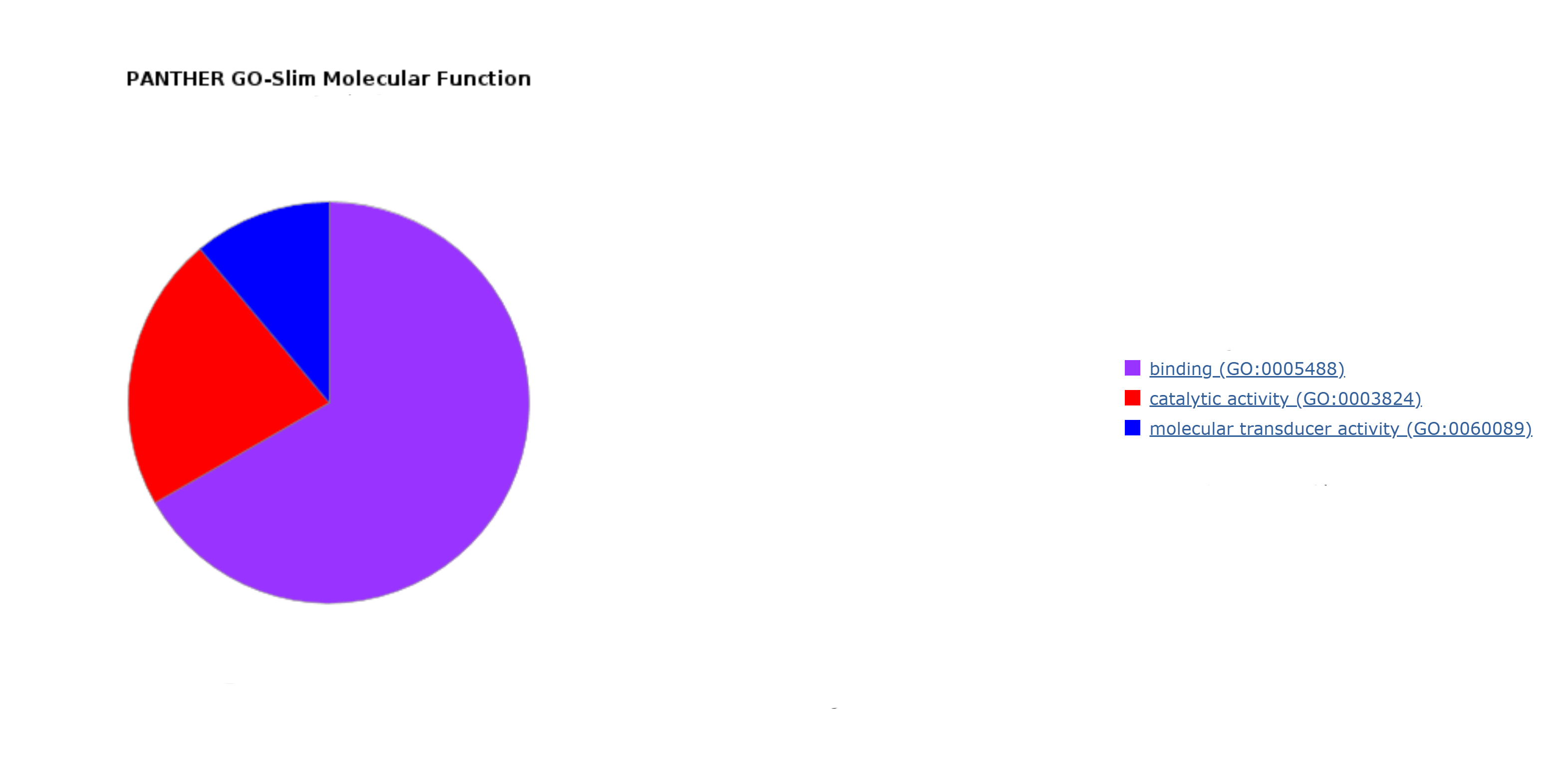 | | 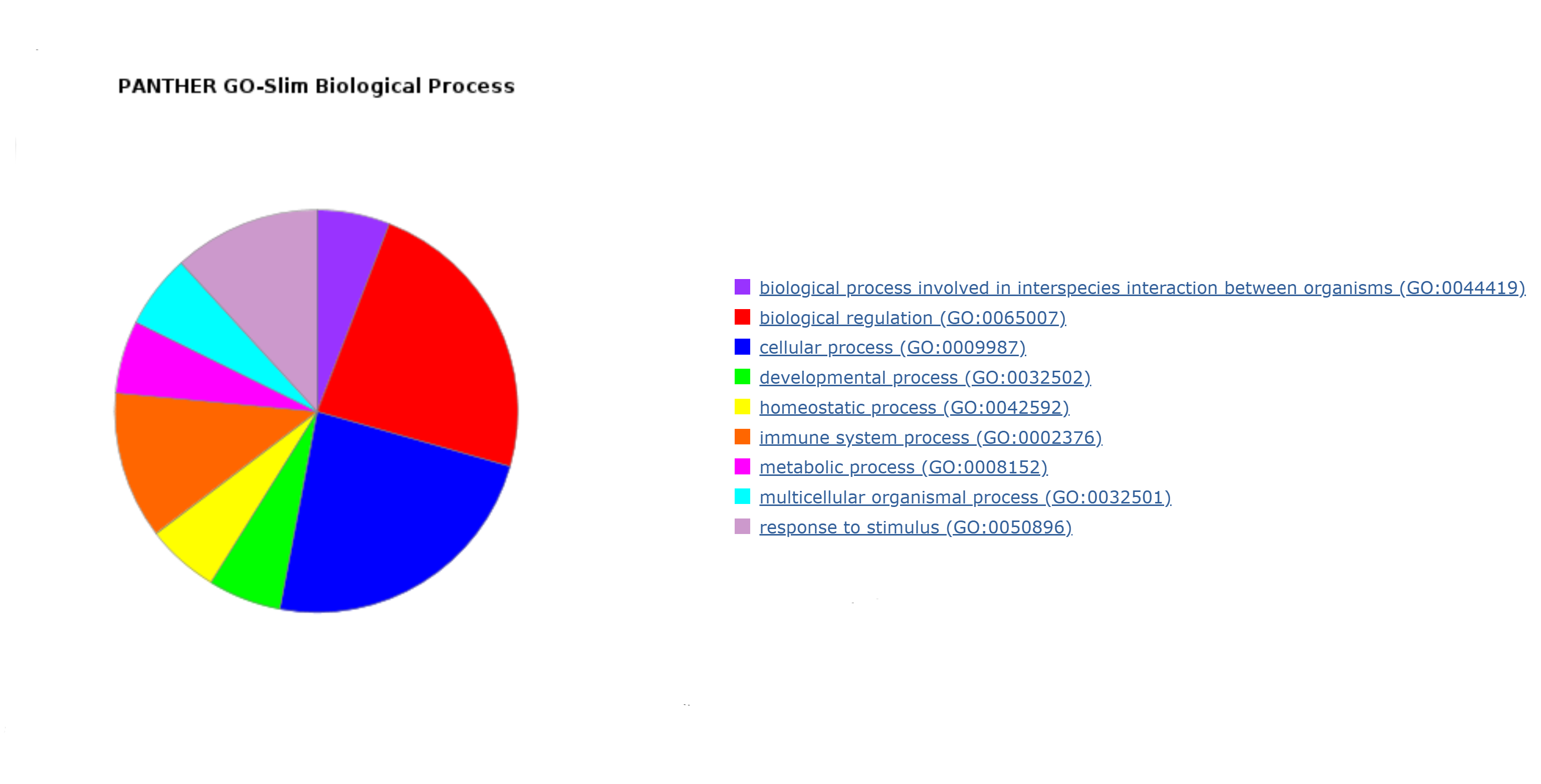 |
| **(h)** | | |
| **Supplementary figure 1**. GO analysis of molecular function and biological process of the up- and down-accumulated proteins in treatment groups; (a) Agarikon Plus group up-accumulated proteins, (b) Agarikon Plus group down-accumulated proteins, (c) combinatorial group (Agarikon Plus with 5-fluorouracil) up-accumulated proteins, (d) combinatorial group (Agarikon Plus with 5-fluorouracil) down-accumulated proteins, (e) combinatorial group (Agarikon Plus with Agarikon.1) up-accumulated proteins, (f) combinatorial group (Agarikon Plus with Agarikon.1) down-accumulated proteins, (g) 5-fluorouracil group up-accumulated proteins, (h) 5-fluorouracil group down-accumulated proteins. | | |


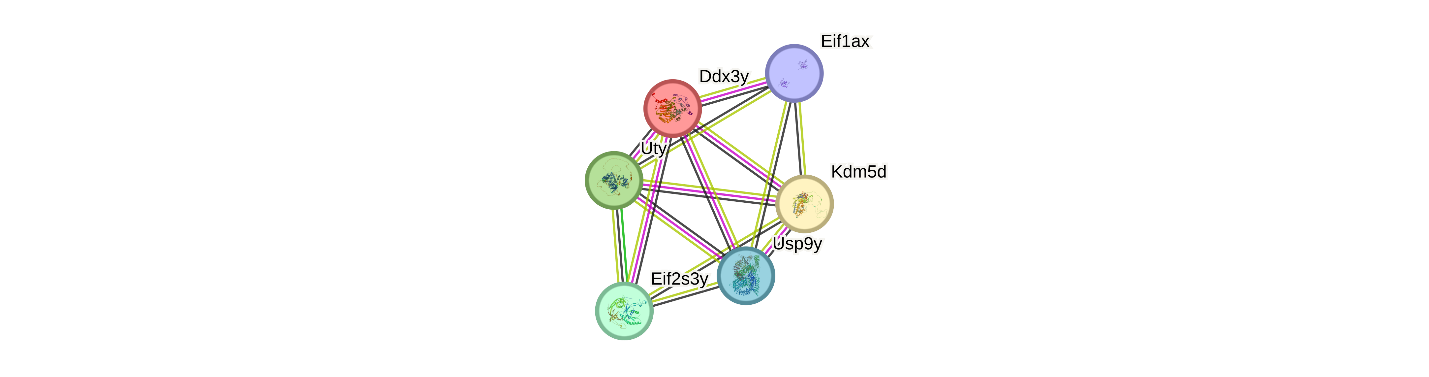


**Supplementary figure 2.** STRING enriched analysis of DDX3Y (ATP-dependent RNA helicase DDX3Y) protein interactions in the Agarikon Plus down-accumulated group.


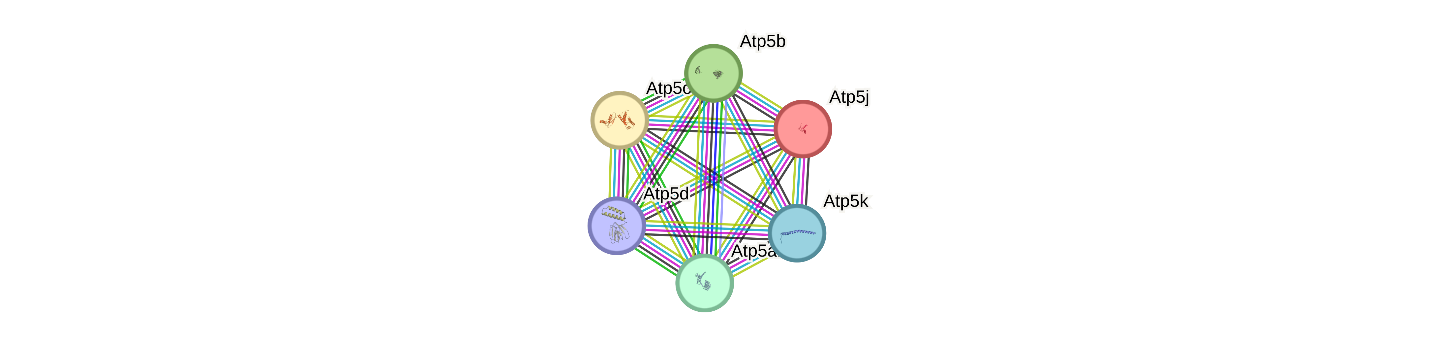


**Supplementary figure 3.** STRING enriched analysis of ATP5j (ATP synthase-coupling factor 6, mitochondrial) protein interactions in the Agarikon Plus down-accumulated group.


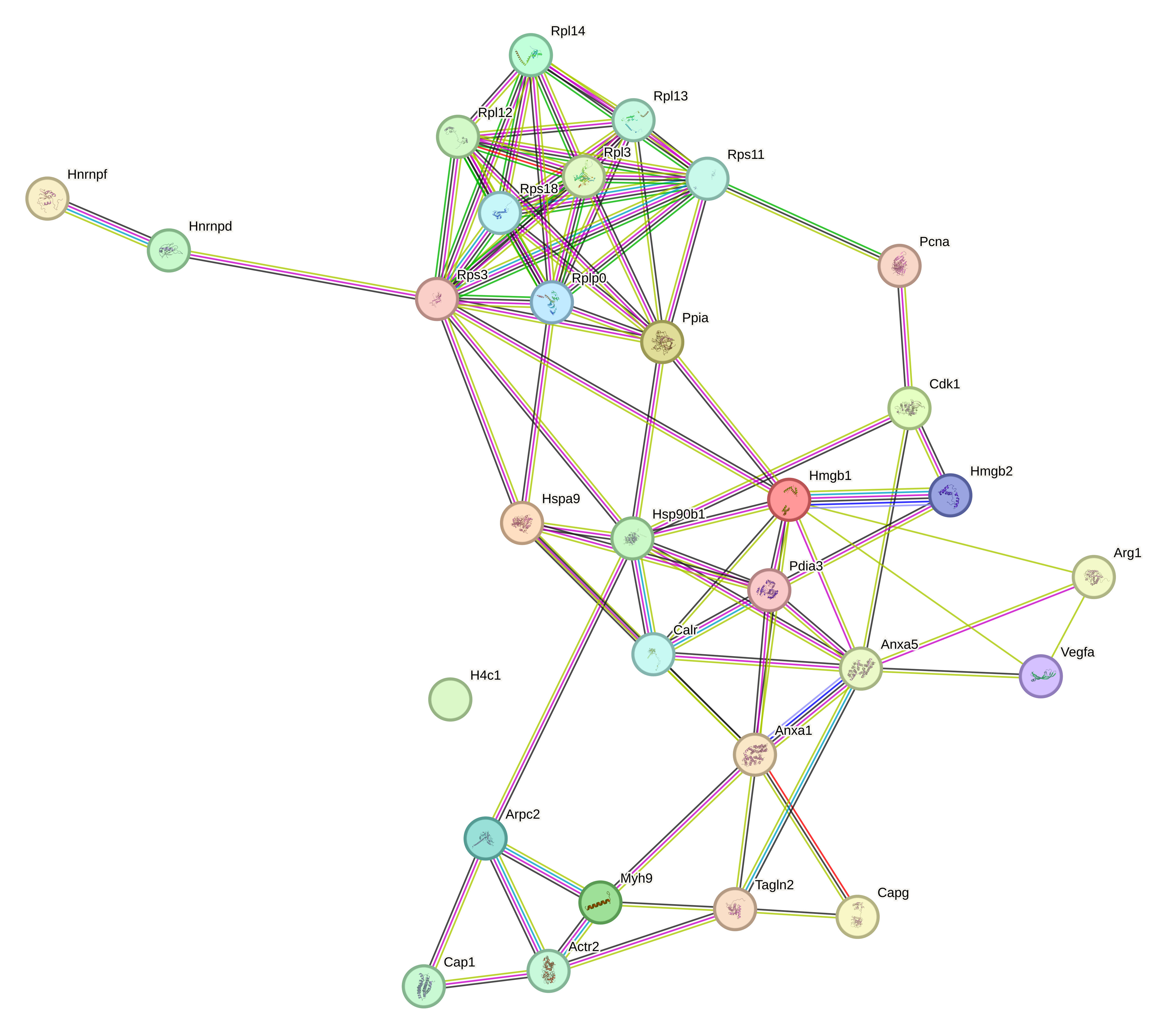


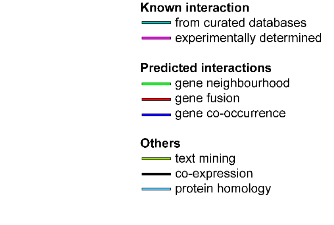


**Supplementary figure 4.** Protein-protein interaction networks identified using STRING for differential proteins in abundance for combinatorial group (AP with 5-FU) down-accumulated proteins, with Arg1 (arginase) and Vegfa (vascular endothelial growth factor A) added on the basis of earlier experiments (Jakopovic et al., 2020b).
